# Supplementary material for: Hidden Tenants: Microbiota of the Rhizosphere and Phyllosphere of Cordia dodecandra Trees in Mayan Forests and Homegardens
Source: Plants (Basel). 2022 Nov 15;11(22):3098. doi: 10.3390/plants11223098 (PMC9699097; doi:10.3390/plants11223098)
Supplement: Supplementary file 1 [file plants-11-03098-s001.zip › plants-1975968-Supplementary material File S1.pdf]

Hidden Tenants: Microbiota of the Rhizosphere and Phyllosphere of *Cordia dodecandra* Trees in Mayan Forests and Homegardens

Carla G. May-Mutul, Miguel A. López-Garrido, Aileen O'Connor-Sánchez, Yuri J. Peña-Ramírez, Natalia Y. Labrín-Sotomayor, Héctor Estrada-Medina and Miriam M. Ferrer

Supplementary material File S1. Pipelines for the bioinformatic analyses in Qiime2 2022.2 of Bacteria (16S) and Fungi (ITS)

```
### PIPELINE FOR BACTERIA METAGENOMIC ANALYSES 16S
### Qiime2 activation
conda activate qiime2-2022.2
### Fastqc análisis
fastqc /*.fastq .
### Multifastqc análisis
multiqc .
### Cut adapters using cut_adapt_loop_bash
bash cut_adapt_loop.bash.sh
READS=…/raw_sequences
CUT_READS=…/cutreads
SAMPLE_NAMES=…/samples.txt
CUT_READ1_suf="_1_cut.fastq.gz"
CUT_READ2_suf="_2_cut.fastq.gz"
IN_READ1_suf="_R1.fastq"
IN_READ2_suf="_R2.fastq"
ls $READS
while read line
do
    sample=$line
    cutadapt --quality-cutoff 20,15 --cores 11 \
    -u 9 \
    -U 9 \
    --adapter TTACTTCCTCTAAATGACCAAG -A
GCATCGATGAAGAACGCAGC\
    --front CTTGGTCATTTAGAGGAAGTAA  -G
GCTGCGTTCTTCATCGATGC\
    -o $CUT_READS/$sample$CUT_READ1_suf \
    -p $CUT_READS/$sample$CUT_READ2_suf \
    $READS/$sample$IN_READ1_suf $READS/$sample$IN_READ2_suf >
$CUT_READS/$sample"_report.txt"
done < $SAMPLE_NAMES
### Import archive in Fastq format
qiime tools import \
```

```
  --type SampleData[PairedEndSequencesWithQuality] \
  --input-format PairedEndFastqManifestPhred33\
  --input-path /*.txt \
  --output-path sequences.qza
### Demuxing
qiime demux summarize \
  --i-data sequences.qza \
  --o-visualization /*.qzv
### Quality control of sequences (denoising) in DADA2
qiime dada2 denoise-paired \
  --i-demultiplexed-seqs trimmed.qza \
  --p-trim-left-f 13 \
  --p-trim-left-r 13 \
  --p-trunc-len-f 220 \
  --p-trunc-len-r 200 \
  --output-dir /
### Create freature tables
qiime feature-table summarize \
  --i-table dada2out/table.qza \
  --o-visualization /*.qzv
qiime metadata tabulate \
  --m-input-file dada2out/denoising_stats.qza \
  --o-visualization /*.qzv
qiime feature-table tabulate-seqs \
  --i-data dada2out/representative_sequences.qza \
  --o-visualization /*.qzv
### Taxonomy classification
# Run a clasifier pre-trained
qiime tools import \
  --type 'FeatureData[Sequence]' \
  --input-path /*.fasta \
  --output-path /*.qza
qiime tools import \
  --type 'FeatureData[Taxonomy]' \
  --input-format HeaderlessTSVTaxonomyFormat \
  --input-path /*.txt \
  --output-path /*.qza
qiime feature-classifier extract-reads \
  --i-sequences *.qza \
  --p-f-primer AGAGTTTGATCCTGGCTCAG \
  --p-r-primer GCTGCCTCCCGTAGGAGT \
  --p-trunc-len 120 \
  --p-min-length 100 \
  --p-max-length 400 \
  --o-reads . ref-seqs.qza\-classifier.qza
qiime feature-classifier fit-classifier-naive-bayes \
  --i-reference-reads ref-seqs.qza \
```

```
  --i-reference-taxonomy ref-taxonomy.qza \
  --o-classifier classifier.qza
### Taxonomy assignament
qiime feature-classifier classify-sklearn \
  --i-classifier ../*.qza \
  --i-reads ../*.qza \
  --o-classification *.qza
qiime metadata tabulate \
  --m-input-file /*.qza \
  --o-visualization /*.qzv
qiime taxa collapse \
  --i-table /*.qza \
  --i-taxonomy /*.qza \
  --p-level 1-7 \
  --o-collapsed-table /*.qza
##Filter host plant's genomes (chloroplast and mitochondria)
qiime taxa filter-table \
  --i-table pe-table.qza \
  --i-taxonomy pe-taxonomy.qza \
  --p-include p__ \
  --p-exclude mitochondria,Mitochondria,Chloroplast,chloroplast \
  --o-filtered-table /*.qza
qiime metadata tabulate \
  --m-input-file /*.qza \
  --o-visualization /*.qzv
### Taxonomy bar chart
qiime taxa barplot \
  --i-table table.qza \
  --i-taxonomy taxonomy.qza \
  --m-metadata-file sample-metadata.tsv \
  --o-visualization /*.qzv
###PIPELINE FOR FUNGI METAGENOMIC ANALYSES ITS
### Qiime2 Activation
conda activate qiime2-2022.2
### Installing bioconda ITSxpress
conda install -c bioconda itsxpress
pip install q2-itsxpress
### fastqc análisis
fastqc ./*.fastq
### multiqc análisis
multiqc .
### Cut adapters using cut_adapt_loop_bash
bash cutadapt_loopITS.bash
READS=…/raw_sequences
CUT_READS=…/cutreads
SAMPLE_NAMES=…/samples.txt
CUT_READ1_suf="_1_cut.fastq.gz"
```

```
CUT_READ2_suf="_2_cut.fastq.gz"
IN_READ1_suf="_R1.fastq"
IN_READ2_suf="_R2.fastq"
ls $READS
while read line
do
    sample=$line
    cutadapt --quality-cutoff 20,15 --cores 11 \
    -u 9 \
    -U 9 \
    --adapter GCATCGATGAAGAACGCAGC -A
TTACTTCCTCTAAATGACCAAG \
    --front CTTGGTCATTTAGAGGAAGTAA  -G
GCTGCGTTCTTCATCGATGC \
    -o $CUT_READS/$sample$CUT_READ1_suf \
    -p $CUT_READS/$sample$CUT_READ2_suf \
    $READS/$sample$IN_READ1_suf $READS/$sample$IN_READ2_suf >
$CUT_READS/$sample"_report.txt"
done < $SAMPLE_NAMES
### Import archive in Fastq format and visualization
qiime tools import \
  --type SampleData[PairedEndSequencesWithQuality] \
  --input-format PairedEndFastqManifestPhred33\
  --input-path manifest.txt \
  --output-path sequences.qza
  qiime demux summarize \
  --i-data sequences.qza \
  --o-visualization sequences.qzv
### Run itsxpress, specifyng the sequenced region
qiime itsxpress trim-pair-output-unmerged \
  --i-per-sample-sequences sequences.qza \
  --p-region ITS1-2 \
  --p-taxa ALL \
  --p-threads 6 \
  --verbose \
  --o-trimmed trimmed.qza
### Quality control of sequences(denoise)in DADA2
qiime dada2 denoise-paired \
  --i-demultiplexed-seqs trimmed.qza \
  --p-trim-left-f 13 \
  --p-trim-left-r 13 \
  --p-trunc-len-f 240 \
  --p-trunc-len-r 200 \
  --output-dir dada2out
### Sequences table visualization
  qiime feature-table summarize \
  --i-table dada2out/table.qza \
```

```
  --o-visualization dada2_tableviz.qzv
### Create freature table
  qiime metadata tabulate \
  --m-input-file dada2out/denoising_stats.qza \
  --o-visualization dada2_stats.qzv
### Ver las secuencias representativas
  qiime feature-table tabulate-seqs \
  --i-data dada2out/representative_sequences.qza \
  --o-visualization dada2_rep-seqs.qzv
### Taxomony classification
### Unzip and import the database with the script ITS_processing_full_dataset.bash
### Download and decompress the database for taxonomy analysis in the database directory
### 8.0    2018-11-18    All eukaryotes    9 409 (RefS)    54 013 (RepS)
### Current    https://doi.org/10.15156/BIO/786335 6DA3F3DDFBACB7D3031FE12EF14C4F5464235C73230C217D14E8C0EAB1042E86.zip
### Import in  qiime2
qiime tools import \
  --type 'FeatureData[Sequence]' \
  --input-path …/sh_refs_qiime_ver8_dynamic_all_02.02.2019.fasta \
  --output-path unite.qza
### Taxonomy  Import (UNITE)
qiime tools import \
  --type 'FeatureData[Taxonomy]' \
  --input-format HeaderlessTSVTaxonomyFormat \
  --input-path …/sh_taxonomy_qiime_ver8_dynamic_all_02.02.2019.txt
  --output-path unite-taxonomy.qza
### Training classifier as: classifier.qza
qiime feature-classifier fit-classifier-naive-bayes \
  --i-reference-reads unite.qza \
  --i-reference-taxonomy unite-taxonomy.qza \
  --o-classifier classifier.qza
### Taxonomy assignament
qiime feature-classifier classify-sklearn \
  --i-classifier ../qiime2-ITSxpress-full-dataset/classifier.qza \
  --i-reads dada2out/representative_sequences.qza \
  --o-classification taxonomy.qza
### metadata tabulate and results visualization
qiime metadata tabulate \
  --m-input-file taxonomy.qza \
  --o-visualization taxonomy.qzv
### Crate metadata file
ITS_metadata.txt
### Taxonomy bar chart (taxa bar-plot)
qiime taxa barplot \
  --i-table dada2out/table.qza  \
```

```
--i-taxonomy taxonomy.qza \
--m-metadata-file sample_metadata.txt \
--o-visualization taxa-bar-plots.qzv
```
