# Supplementary material for: Hidden Tenants: Microbiota of the Rhizosphere and Phyllosphere of Cordia dodecandra Trees in Mayan Forests and Homegardens
Source: Plants (Basel). 2022 Nov 15;11(22):3098. doi: 10.3390/plants11223098 (PMC9699097; doi:10.3390/plants11223098)
Supplement: Supplementary file 1 [file plants-11-03098-s001.zip › plants-1975968-Supplementary material File S2.pdf]

Hidden Tenants: Microbiota of the Rhizosphere and Phyllosphere of *Cordia dodecandra* Trees in Mayan Forests and Homegardens

Carla G. May-Mutul, Miguel A. López-Garrido, Aileen O'Connor-Sánchez, Yuri J. Peña-Ramírez, Natalia Y. Labrín-Sotomayor, Héctor Estrada-Medina and Miriam M. Ferrer

Supplementary material File S2. Pipeline for the bioinformatic analyses in phyloseq for the alpha and beta diversity analysis of Bacteria and Fungi microbiota

```
###THE PIPELINE PRESENTED WAS MODIFIED FROM
https://www.yanh.org/2021/01/01/microbiome-r/ AND WAS USED FOR THE
BACTERIAL MICROBIOTA,
###A SIMILAR ONE WAS USED TO PERFORM THE FUNGAL MICROBIOTA
ANALYSES
#load needed libraries
library(tidyverse)
library(vegan)
library(BiocManager)
library(phyloseq)
library(ANCOMBC)
library(DESeq2)
library(ComplexHeatmap)
##read the feature tables and taxonomy (exported from bioformat to tsv in R)
otu <- read.table(file = "feature-table.tsv", sep = "\t", header = T, row.names = 1,
        skip = 1, comment.char = "")
taxonomy <- read.table(file = "taxonomy.tsv", sep = "\t", header = T ,row.names =
1)
# Clean the taxonomy, Greengenes format
tax <- taxonomy %>%
  select(Taxon) %>%
  separate(Taxon, c("Kingdom", "Phylum", "Class", "Order", "Family", "Genus",
"Species"), ";")
tax.clean <- data.frame(row.names = row.names(tax),
            Kingdom = str_replace(tax[,1], "k__",""),
            Phylum = str_replace(tax[,2], "p__",""),
            Class = str_replace(tax[,3], "c__",""),
            Order = str_replace(tax[,4], "o__",""),
            Family = str_replace(tax[,5], "f__",""),
            Genus = str_replace(tax[,6], "g__",""),
            Species = str_replace(tax[,7], "s__",""),
            stringsAsFactors = FALSE)
tax.clean[is.na(tax.clean)] <- ""
tax.clean[tax.clean=="__"] <- ""
```

```
for (i in 1:nrow(tax.clean)){
  if (tax.clean[i,7] != ""){
    tax.clean$Species[i] <- paste(tax.clean$Genus[i], tax.clean$Species[i], sep = " ")
  } else if (tax.clean[i,2] == ""){
    kingdom <- paste("Unclassified", tax.clean[i,1], sep = " ")
    tax.clean[i, 2:7] <- kingdom
  } else if (tax.clean[i,3] == ""){
    Phylum <- paste("Unclassified", tax.clean[i,2], sep = " ")
    tax.clean[i, 3:7] <- Phylum
  } else if (tax.clean[i,4] == ""){
    class <- paste("Unclassified", tax.clean[i,3], sep = " ")
    tax.clean[i, 4:7] <- class
  } else if (tax.clean[i,5] == ""){
    order <- paste("Unclassified", tax.clean[i,4], sep = " ")
    tax.clean[i, 5:7] <- order
  } else if (tax.clean[i,6] == ""){
    family <- paste("Unclassified", tax.clean[i,5], sep = " ")
    tax.clean[i, 6:7] <- family
  } else if (tax.clean[i,7] == ""){
    tax.clean$Species[i] <- paste("Unclassified ",tax.clean$Genus[i], sep = " ")}}
### Create a phyloseq object (feature table, taxonomy and sample metadata --
should be in qiime2 tsv format)
metadata <- read.table(file = "sample-metadata.tsv", sep = "\t", header = T,
row.names = 1)
OTU = otu_table(as.matrix(otu), taxa_are_rows = TRUE)
TAX = tax_table(as.matrix(tax.clean))
SAMPLE <- sample_data(metadata)
# merge the data
ps <- phyloseq(OTU, TAX, SAMPLE)
## Rarefaction curves
psdata <- ps
psdata
sample_sums(psdata)
set.seed(42)
#Rarefaction estimates
calculate_rarefaction_curves <- function(psdata, measures, depths) {
  require('plyr') # ldply
  require('reshape2') # melt
#Alpha diversity estimates
  estimate_rarified_richness <- function(psdata, measures, depth) {
    if(max(sample_sums(psdata)) < depth) return()
    psdata <- prune_samples(sample_sums(psdata) >= depth, psdata)
#Normalization of data
    rarified_psdata <- rarefy_even_depth(psdata, depth, verbose = FALSE)
    alpha_diversity <- estimate_richness(rarified_psdata, measures = measures)
#as.matrix forces the use of melt.array, which includes the Sample names
(rownames)
```

```
            molten_alpha_diversity <- melt(as.matrix(alpha_diversity), varnames =
c('Sample', 'Measure'), value.name = 'Alpha_diversity')
            molten_alpha_diversity}
          names(depths) <- depths # this enables automatic addition of the Depth to the
output by ldply
          rarefaction_curve_data <- ldply(depths, estimate_rarified_richness, psdata =
psdata, measures = measures, .id = 'Depth', .progress = ifelse(interactive(), 'text', 'none'))
        # convert Depth from factor to numeric
          rarefaction_curve_data$Depth <-
as.numeric(levels(rarefaction_curve_data$Depth))[rarefaction_curve_data$Depth]
          rarefaction_curve_data}
        rarefaction_curve_data <- calculate_rarefaction_curves(psdata, c('Observed'),
rep(c(1, 10, 100, 1000, 1:100 * 10000), each = 10))
        summary(rarefaction_curve_data)
        rarefaction_curve_data_summary <- ddply(rarefaction_curve_data, c('Depth',
'Sample', 'Measure'),
            summarise, Alpha_diversity_mean = mean(Alpha_diversity), Alpha_diversity_sd
= sd(Alpha_diversity))
        rarefaction_curve_data_summary_verbose <-
merge(rarefaction_curve_data_summary,
             data.frame(sample_data(psdata)), by.x = 'Sample', by.y = 'row.names')
        ##Plot rarefaction curves
        ggplot(data = rarefaction_curve_data_summary_verbose,
            mapping = aes(x = Depth,
            y = Alpha_diversity_mean,
            ymin = Alpha_diversity_mean - Alpha_diversity_sd,
            ymax = Alpha_diversity_mean + Alpha_diversity_sd,
            colour = Compartment, shape = Population, ##groups
            group = Sample)) +
          ggtitle('Bacteria') + ylab('Observed') + ##modify the title and labels
          geom_line() +
          geom_pointrange() +
          facet_wrap(facets = ~ Compartment, scales = 'free_y') +
          theme_classic() +
          guides(color = FALSE) +
          theme(axis.text.x.bottom = element_text(angle = 0), legend.position = "bottom") +
          scale_x_continuous(trans='log10') +
          scale_y_continuous(trans='log10') +
          scale_color_manual(values = c("cyan4","chocolate4"))
        ##ALPHA DIVERSITY ESTIMATES AND WILCOXON COMPARISONS
        ##rarefy the estimates (sample size is included to established the cut, otherwise the
minimal sample size is used)
        set.seed(111) # keep result reproductive
        ps.rarefied = rarefy_even_depth(ps, rngseed=1, sample.size=1000, replace=F)
        #Alpha diversity estimates'plot per compartment
        plot_richness(ps.rarefied, x="Compartment", color = "Compartment",
measures=c("Observed", "Chao1", "Shannon")) +
```

```
  geom_violin() +
  geom_boxplot(width=0.1, fill="white") +
  stat_summary(fun.y=median, geom="point", size=2, color = "black") +
  theme_classic() +
  theme(axis.text.x.bottom = element_blank(), legend.position = "bottom",
  axis.title.x = element_blank()) +
  scale_color_manual(values = c("cyan4","chocolate4"))
##Wilcoxon test was used to compare the two medians (phyllosphere and
rhizosphere) for each alpha diversity measure obtaining a table
rich = estimate_richness(ps.rarefied, measures = c("Observed", "Chao1",
"Shannon"))
wilcox.observed <- pairwise.wilcox.test(rich$Observed,
                                         sample_data(ps.rarefied)$Compartment,
                                         p.adjust.method = "BH")
tab.observed <- wilcox.observed$p.value %>%
  as.data.frame() %>%
  tibble::rownames_to_column(var = "group1") %>%
  gather(key="group2", value="p.adj", -group1) %>%
  na.omit()
tab.observed
wilcox.chao1 <- pairwise.wilcox.test(rich$Chao1,
                                      sample_data(ps.rarefied)$Compartment,
                                      p.adjust.method = "BH")
tab.chao1 <- wilcox.chao1$p.value %>%
  as.data.frame() %>%
  tibble::rownames_to_column(var = "group1") %>%
  gather(key="group2", value="p.adj", -group1) %>%
  na.omit()
tab.chao1
wilcox.shannon <- pairwise.wilcox.test(rich$Shannon,
                                      sample_data(ps.rarefied)$Compartment,
                                      p.adjust.method = "BH")
tab.shannon <- wilcox.shannon$p.value %>%
  as.data.frame() %>%
  tibble::rownames_to_column(var = "group1") %>%
  gather(key="group2", value="p.adj", -group1) %>%
  na.omit()
tab.shannon
## Repeat for all factors
##Beta diversity evaluation
#Estimation of Bray distances
dist = phyloseq::distance(ps.rarefied, method="bray")
ordination = ordinate(ps.rarefied, method="PCoA", distance=dist)
dist = phyloseq::distance(ps.rarefied, method="bray")
ordination = ordinate(ps.rarefied, method="PCoA", distance=dist)
#Plot principal coordinates (two axis)
plot_ordination(ps.rarefied, ordination, type="samples", title = 'Bacteria',
```

```
        shape = "Population", color = "Compartment") +
    scale_color_manual(values = c("cyan4","chocolate4")) +
    geom_vline(aes(xintercept = 0), color ="gray") +
    geom_hline(aes(yintercept = 0), color ="gray") +
    theme_classic() +
    theme(panel.border = element_rect(color ="black", fill=NA),
    axis.line.x = element_blank(), axis.line.y = element_blank(), strip.background =
element_blank())
##Species barplot in two rows (Compartment and Population)
ps.rel = transform_sample_counts(ps, function(x) x/sum(x)*100)
# agglomerate taxa
glom <- tax_glom(ps.rel, taxrank = 'Species', NArm = FALSE)
ps.melt <- psmelt(glom)
# change to character for easy-adjusted level
ps.melt$Species <- as.character(ps.melt$Species)
ps.melt <- ps.melt %>%
  group_by(Compartment, Population, Species) %>%
  mutate(median=median(Abundance))
# select group mean > 1
keep <- unique(ps.melt$Species[ps.melt$median > 2.5])
ps.melt$Species[!(ps.melt$Species %in% keep)] <- "< 2.5%"
#to get the same rows together
ps.melt_sum <- ps.melt %>%
  group_by(Sample,Compartment,Population,Species) %>%
  summarise(Abundance=sum(Abundance))
##Relative abundance analyses at Species level (Repeat for each taxa level, replace
Species by Genus for instance)
ps.rel = transform_sample_counts(ps, function(x) x/sum(x)*100)
# agglomerate taxa
glom <- tax_glom(ps.rel, taxrank = 'Species', NArm = FALSE)
ps.melt <- psmelt(glom)
# change to character for easy-adjusted level
ps.melt$Species <- as.character(ps.melt$Species)
ps.melt <- ps.melt %>%
  group_by(Compartment, Population, Species) %>%
  mutate(median=median(Abundance))
# select group mean > 1
keep <- unique(ps.melt$Species[ps.melt$median > 2.5])
ps.melt$Species[!(ps.melt$Species %in% keep)] <- "< 2.5%"
#to get the same rows together
ps.melt_sum <- ps.melt %>%
  group_by(Sample,Compartment,Population,Species) %>%
  summarise(Abundance=sum(Abundance))
#Plot for relative abundance in two rows (comparisons for two factors)
ggplot(ps.melt_sum, aes(x = Sample, y = Abundance, fill = Species)) +
  geom_bar(stat = "identity", aes(fill=Species)) +
  labs(x="", y="%") +
```

```
  facet_wrap(~Compartment + Population, scales= "free_x", nrow=2) +
  theme_classic() +
  theme(legend.position = "right",
        strip.background = element_blank(),
        axis.text.x.bottom = element_text(angle = 0))
##Differential sequence analyses and heatmap
sample_data(ps)$Compartment <- as.factor(sample_data(ps)$Compartment) #
factorize for DESeq2
ps.taxa <- tax_glom(ps, taxrank = 'Species', NArm = FALSE)
ps.taxa.pse <- ps.taxa
otu_table(ps.taxa.pse) <- otu_table(ps.taxa) + 1
# pairwise comparison between Phyllosphere and Rhizosphere
ps.taxa.pse.sub <- subset_samples(ps.taxa.pse, Compartment %in%
c("phyllosphere", "rhizosphere"))
ds = phyloseq_to_deseq2(ps.taxa.pse.sub, ~ Compartment)
ds = DESeq(ds, test="Wald", fitType="parametric")
alpha = 0.05
res = results(ds, alpha=alpha)
res = res[order(res$padj, na.last=NA), ]
taxa_sig = rownames(res[1:20, ]) # select bottom 20 with lowest p.adj values
ps.taxa.rel <- transform_sample_counts(ps, function(x) x/sum(x)*100)
ps.taxa.rel.sig <- prune_taxa(taxa_sig, ps.taxa.rel)
# Only keep Phyllosphere and Rhizosphere samples
ps.taxa.rel.sig <- prune_samples(colnames(otu_table(ps.taxa.pse.sub)),
ps.taxa.rel.sig)
matrix <- as.matrix(data.frame(otu_table(ps.taxa.rel.sig)))
rownames(matrix) <- as.character(tax_table(ps.taxa.rel.sig)[, "Species"])
metadata_sub <- data.frame(sample_data(ps.taxa.rel.sig))
# Define the annotation color for columns and rows
annotation_col = data.frame(
   `Population` = as.factor(metadata_sub$Population),
   `Compartment` = as.factor(metadata_sub$Compartment),
   check.names = FALSE)
rownames(annotation_col) = rownames(metadata_sub)
annotation_row = data.frame(
   Phylum = as.factor(tax_table(ps.taxa.rel.sig)[, "Phylum"]))
rownames(annotation_row) = rownames(matrix)
# ann_color should be named vectors
Phylum_col = RColorBrewer::brewer.pal(length(levels(annotation_row$Phylum)),
"Paired")
names(Phylum_col) = levels(annotation_row$Phylum)
ann_colors = list(
   `Population` = c(`Homegarden` = "darkgoldenrod", `Forest` = "darkgreen"),
   `Compartment` = c(`phyllosphere` = "cyan4", `rhizosphere` = "chocolate4"),
   Phylum = Phylum_col)
###ANCOMBC
# pairwise comparison between phyllosphere and rhizosphere
```

```
ps.taxa.sub <- subset_samples(ps.taxa, Compartment %in% c("phyllosphere",
"rhizosphere"))
# ancombc
out <- ancombc(phyloseq = ps.taxa.sub, formula = "Compartment",
        p_adj_method = "holm", zero_cut = 0.90, lib_cut = 1000,
        group = "Compartment", struc_zero = TRUE, neg_lb = TRUE, tol = 1e-5,
        max_iter = 100, conserve = TRUE, alpha = 0.05, global = TRUE)
res <- out$res
# select the bottom 20 with lowest p values
res.or_p <-
rownames(res$q_val[,"Compartmentphyllosphere"])[base::order(res$q_val[,"Comparmentr
hizosphere"])]
taxa_sig <- res.or_p[1:20]
ps.taxa.rel.sig <- prune_taxa(taxa_sig, ps.taxa.rel)
# Only keep phyllosphere and rhizosphere samples
ps.taxa.rel.sig <- prune_samples(colnames(otu_table(ps.taxa.sub)), ps.taxa.rel.sig)
#construct heatmap with complexheatmap
matrix <- as.matrix(data.frame(otu_table(ps.taxa.rel.sig)))
rownames(matrix) <- as.character(tax_table(ps.taxa.rel.sig)[, "Species"])
metadata_sub <- data.frame(sample_data(ps.taxa.rel.sig))
# Define the annotation color for columns and rows
annotation_col = data.frame(
   `Population`= as.factor(metadata_sub$Population),
  `Compartment` = as.factor(metadata_sub$Compartment),
  check.names = FALSE)
rownames(annotation_col) = rownames(metadata_sub)
annotation_row = data.frame(
  Phylum = as.factor(tax_table(ps.taxa.rel.sig)[, "Phylum"]))
rownames(annotation_row) = rownames(matrix)
# ann_color should be named vectors
Phylum_col = RColorBrewer::brewer.pal(length(levels(annotation_row$Phylum)),
"Paired")
names(Phylum_col) = levels(annotation_row$Phylum)
ann_colors = list(
  `Population` = c(`Homegarden` = "darkgoldenrod", `Forest` = "darkgreen"),
  `Compartment` = c(`phyllosphere` = "cyan4", `rhizosphere` = "chocolate4"),
  Phylum = Phylum_col)
ComplexHeatmap::pheatmap(matrix, scale= "row",
           annotation_col = annotation_col,
           annotation_row = annotation_row,
           annotation_colors = ann_colors)
```
