# Supplementary material for: Hidden Tenants: Microbiota of the Rhizosphere and Phyllosphere of Cordia dodecandra Trees in Mayan Forests and Homegardens
Source: Plants (Basel). 2022 Nov 15;11(22):3098. doi: 10.3390/plants11223098 (PMC9699097; doi:10.3390/plants11223098)
Supplement: Supplementary file 1 [file plants-11-03098-s001.zip › plants-1975968-Supplementary material Table S1.pdf]

# Hidden Tenants: Microbiota of the Rhizosphere and Phyllosphere of *Cordia dodecandra* Trees in Mayan Forests and Homegardens

**Carla G. May-Mutul, Miguel A. López-Garrido, Aileen O'Connor-Sánchez, Yuri J. Peña-Ramírez, Natalia Y. Labrín-Sotomayor, Héctor Estrada-Medina and Miriam M. Ferrer**

**Supplementary material Table S1.** Taxonomic classification assigned to 618 bacteria OTUs from phyllosphere (phy) and rhizosphere (rhi) *Cordia dodecandra* microbiota in Yucatán´s forest (for) and homegarden (hom) populations of the Northeast (NE) and Southwest (SW) region. The last 8 columns present the percent (relative frequency) per sample coded by region, population, and compartment.

| Class | Order | Family | Genus | Species | SW hom phy | NE hom phy | SW for phy | NE for phy | SW hom rhi | NE hom rhi | SW for rhi | NE for rhi |
|---|---|---|---|---|---|---|---|---|---|---|---|---|
| **Acidobacteriota** | | | | | | | | | | | | |
| Acidobacteriae | Acidobacteriae | Acidobacteriae | *Paludibaculum* | metagenome | | | | | | | | 0.031 |
| Acidobacteriae | Acidobacteriales | Acidobacteriaceae (Subgroup_1) | *Edaphobacter* | unidentified | | | | | 0.035 | | | |
| Acidobacteriae | Acidobacteriales | Koribacteraceae | *Candidatus_Koribacter* | unidentified | | | | | | | | 0.157 |
| Acidobacteriae | Acidobacteriales | uncultured | uncultured | unidentified | | | | | 0.165 | | | |
| Acidobacteriae | Bryobacterales | Bryobacteraceae | *Bryobacter* | uncultured *Acidobacteriaceae* | | | | | 0.045 | | | |
| Acidobacteriae | Bryobacterales | Bryobacteraceae | *Bryobacter* | unidentified | | | | | 0.229 | 0.148 | 0.276 | 0.159 |
| Acidobacteriae | PAUC26f | PAUC26f | PAUC26f | uncultured_*Solibacteraceae* | | | | | | | 0.023 | |
| Acidobacteriae | Solibacterales | Solibacteraceae | *Candidatus_Solibacter* | uncultured_*Acidobacteriales* | | | | | | | 0.032 | |
| Acidobacteriae | Solibacterales | Solibacteraceae | *Candidatus_Solibacter* | unidentified | | | | | | | | 0.128 |
| Acidobacteriae | Subgroup_2 | Subgroup_2 | Subgroup_2 | unidentified | | | | | 0.045 | | | |
| Acidobacteriae | unidentified | unidentified | unidentified | unidentified | | | | | 0.055 | | 0.051 | |

| | | | | | | | | | | | | |
|---|---|---|---|---|---|---|---|---|---|---|---|---|
| Blastocatellia | 45597 | 45597 | nov-24 | uncultured_bacterium | | | | | 0.588 | | 0.839 | 0.335 |
| Blastocatellia | 45597 | 45597 | nov-24 | unidentified | | | | | 0.13 | | 0.097 | 0.084 |
| Blastocatellia | Blastocatellales | Blastocatellaceae | *Blastocatella* | uncultured_bacterium | | 0.182 | | | | | | |
| Blastocatellia | Blastocatellales | Blastocatellaceae | *Blastocatella* | unidentified | | | | | 0.055 | | 0.074 | |
| Blastocatellia | Blastocatellales | Blastocatellaceae | uncultured | unidentified | | | | | 0.095 | | | 0.172 |
| Blastocatellia | Blastocatellales | Blastocatellaceae | unidentified | unidentified | | | | | 0.115 | | 0.29 | |
| Blastocatellia | DS-100 | DS-100 | DS-100 | uncultured_*Acidobacteria* | | | | | 0.06 | | | 0.031 |
| Blastocatellia | DS-100 | DS-100 | DS-100 | unidentified | | | | | | | | 0.09 |
| Blastocatellia | Elev-16S-573 | Elev-16S-573 | Elev-16S-573 | uncultured_*Acidobacteria* | | | | | 0.389 | | 0.585 | 1.593 |
| Blastocatellia | Pyrinomonadales | Pyrinomonadaceae | RB41 | *Arenimicrobium luteum* | | | | | 0.324 | | | |
| Blastocatellia | Pyrinomonadales | Pyrinomonadaceae | RB41 | uncultured_*Acidobacteria* | | | | | | | 0.129 | 0.15 |
| Blastocatellia | Pyrinomonadales | Pyrinomonadaceae | RB41 | uncultured_*Acidobacteriaceae* | | | | | 0.189 | | 0.272 | |
| Blastocatellia | Pyrinomonadales | Pyrinomonadaceae | RB41 | uncultured_*Acidobacteriales* | | | | | 0.284 | | | |
| Blastocatellia | Pyrinomonadales | Pyrinomonadaceae | RB41 | uncultured_*Acidobacterium* | | | | | 0.055 | | | |
| Blastocatellia | Pyrinomonadales | Pyrinomonadaceae | RB41 | unidentified | | | | | 2.268 | 0.445 | 1.484 | 0.655 |
| Holophagae | Subgroup_7 | Subgroup_7 | Subgroup_7 | metagenome | | | | | | | | 0.088 |
| Holophagae | Subgroup_7 | Subgroup_7 | Subgroup_7 | uncultured_*Acidobacteria* | | | | | | | | 0.046 |
| Holophagae | Subgroup_7 | Subgroup_7 | Subgroup_7 | uncultured_candidate | | | | | | | 0.055 | 0.015 |
| Holophagae | Subgroup_7 | Subgroup_7 | Subgroup_7 | uncultured_*Desulfovirga* | | | | | 0.174 | | 0.189 | |
| Holophagae | Subgroup_7 | Subgroup_7 | Subgroup_7 | unidentified | | | | | 0.499 | | 0.161 | 0.071 |
| Subgroup_11 | Subgroup_11 | Subgroup_11 | Subgroup_11 | uncultured_bacterium | | | | | | | 0.203 | 0.126 |

| | | | | | | | | | | | | |
|---|---|---|---|---|---|---|---|---|---|---|---|---|
| Subgroup_18 | Subgroup_18 | Subgroup_18 | Subgroup_18 | uncultured_bacterium | | | | | 0.115 | | 0.336 | 0.66 |
| Subgroup_22 | Subgroup_22 | Subgroup_22 | Subgroup_22 | uncultured_proteobacterium | | | | | | | | 0.042 |
| Subgroup_22 | Subgroup_22 | Subgroup_22 | Subgroup_22 | unidentified | | | | | | | 0.074 | 0.064 |
| Subgroup_25 | Subgroup_25 | Subgroup_25 | Subgroup_25 | uncultured_bacterium | | | | | 0.035 | | 0.041 | 0.384 |
| Subgroup_25 | Subgroup_25 | Subgroup_25 | Subgroup_25 | uncultured_soil | | | | | 0.105 | | 0.065 | 2.622 |
| Subgroup_25 | Subgroup_25 | Subgroup_25 | Subgroup_25 | unidentified | | | | | | | | 0.097 |
| Subgroup_5 | Subgroup_5 | Subgroup_5 | Subgroup_5 | uncultured_*Acidobacteria* | | | | | | | 0.083 | |
| Subgroup_5 | Subgroup_5 | Subgroup_5 | Subgroup_5 | uncultured_bacterium | | | | | 0.229 | 0.148 | 0.765 | 0.057 |
| Subgroup_5 | Subgroup_5 | Subgroup_5 | Subgroup_5 | unidentified | | | | | | | 0.088 | |
| Thermoanaerobaculia | Thermoanaerobaculales | Thermoanaerobaculaceae | Subgroup_10 | *Acidobacteria_bacterium* | | | | | 0.115 | | | |
| Thermoanaerobaculia | Thermoanaerobaculales | Thermoanaerobaculaceae | Subgroup_10 | uncultured_*Alphaproteobacteria* | | | | | 0.15 | | | |
| Thermoanaerobaculia | Thermoanaerobaculales | Thermoanaerobaculaceae | Subgroup_10 | uncultured_*Bacteroidetes* | | | | | | | | 0.035 |
| Thermoanaerobaculia | Thermoanaerobaculales | Thermoanaerobaculaceae | Subgroup_10 | uncultured_*Holophaga* | | | | | | | | 0.075 |
| Thermoanaerobaculia | Thermoanaerobaculales | Thermoanaerobaculaceae | Subgroup_10 | unidentified | | | | | | | 0.083 | 0.34 |
| unidentified | unidentified | unidentified | unidentified | unidentified | | | | | | | | 1.772 |
| Vicinamibacteria | Subgroup_17 | Subgroup_17 | Subgroup_17 | uncultured_bacterium | | | | | | | | 0.06 |
| Vicinamibacteria | Subgroup_17 | Subgroup_17 | Subgroup_17 | unidentified | | | | | | | 0.166 | 0.287 |
| Vicinamibacteria | Vicinamibacterales | uncultured | uncultured | uncultured_*Acidobacteriales* | | | | | | | | 0.7 |
| Vicinamibacteria | Vicinamibacterales | uncultured | uncultured | unidentified | | | | | 0.254 | | 0.926 | 3.416 |
| Vicinamibacteria | Vicinamibacterales | unidentified | unidentified | unidentified | | | | | 0.668 | | 1.078 | 3.467 |

| | | | | | | | | | | | | |
|---|---|---|---|---|---|---|---|---|---|---|---|---|
| Vicinamibacteria | Vicinamibacterales | Vicinamibacteraceae | *Luteitalea* | unidentified | | | | | | | | 0.166 |
| Vicinamibacteria | Vicinamibacterales | Vicinamibacteraceae | uncultured | uncultured_*Acidobacteria* | | | | | | | 0.005 | |
| Vicinamibacteria | Vicinamibacterales | Vicinamibacteraceae | uncultured | uncultured_bacterium | | | | | | | | 0.386 |
| Vicinamibacteria | Vicinamibacterales | Vicinamibacteraceae | uncultured | unidentified | | | | | | | 0.129 | |
| Vicinamibacteria | Vicinamibacterales | Vicinamibacteraceae | unidentified | unidentified | | | | | | | 0.009 | 0.556 |
| Vicinamibacteria | Vicinamibacterales | Vicinamibacteraceae | *Vicinamibacter* | unidentified | | | | | 0.135 | | 0.18 | |
| Vicinamibacteria | Vicinamibacterales | Vicinamibacteraceae | *Vicinamibacteraceae* | uncultured_*Saccharibacillus* | | | | | | | 0.129 | |
| Vicinamibacteria | Vicinamibacterales | Vicinamibacteraceae | *Vicinamibacteraceae* | unidentified | | | | | 1.61 | 0.371 | 3.023 | 4.996 |
| **Actinobacteriota** | | | | | | | | | | | | |
| Acidimicrobiia | Actinomarinales | uncultured | uncultured | bacterium_YC-ZSS-LKJ173 | | | | | | | 0.124 | |
| Acidimicrobiia | IMCC26256 | IMCC26256 | IMCC26256 | metagenome | | | | | 0.135 | | 0.161 | 0.108 |
| Acidimicrobiia | IMCC26256 | IMCC26256 | IMCC26256 | uncultured_*Acidimicrobiia* | | | | | | | 0.078 | |
| Acidimicrobiia | IMCC26256 | IMCC26256 | IMCC26256 | uncultured_bacterium | | | | | 0.145 | | 0.401 | 0.113 |
| Acidimicrobiia | IMCC26256 | IMCC26256 | IMCC26256 | unidentified | | | | | 0.085 | | 0.106 | 0.285 |
| Acidimicrobiia | Microtrichales | Iamiaceae | *Iamia* | unidentified | | | | | | | | 0.062 |
| Acidimicrobiia | Microtrichales | Ilumatobacteraceae | uncultured | uncultured_*Acidimicrobiaceae* | | | | | | | 0.041 | 0.119 |
| Acidimicrobiia | Microtrichales | Ilumatobacteraceae | uncultured | uncultured_bacterium | | | | | 0.06 | | | 0.342 |
| Acidimicrobiia | Microtrichales | Ilumatobacteraceae | uncultured | uncultured_soil | | | | | | | | 0.095 |
| Acidimicrobiia | Microtrichales | Ilumatobacteraceae | uncultured | unidentified | | | | | | | 0.028 | 0.225 |

| | | | | | | | | | | | | |
|---|---|---|---|---|---|---|---|---|---|---|---|---|
| Acidimicrobiia | Microtrichales | Microtrichaceae | uncultured | uncultured_*Actinomycetales* | | | | | | | | 0.046 |
| Acidimicrobiia | Microtrichales | uncultured | uncultured | uncultured_*Acidimicrobiia* | | | | | | | | 0.068 |
| Acidimicrobiia | Microtrichales | uncultured | uncultured | uncultured_bacterium | | | | | 0.518 | 0.698 | 0.327 | 0.382 |
| Acidimicrobiia | Microtrichales | uncultured | uncultured | unidentified | | | | | | | 1.313 | 0.216 |
| Acidimicrobiia | Microtrichales | unidentified | unidentified | unidentified | | | | | | | | 0.071 |
| Acidimicrobiia | uncultured | uncultured | uncultured | unidentified | | | | | | | | 0.132 |
| Acidimicrobiia | unidentified | unidentified | unidentified | unidentified | | | | | 0.115 | 0.193 | 0.276 | 0.492 |
| Actinobacteria | 0319-7L14 | 0319-7L14 | 0319-7L14 | uncultured_actinobacterium | | | | | | | 0.018 | 0.06 |
| Actinobacteria | 0319-7L14 | 0319-7L14 | 0319-7L14 | uncultured_bacterium | | | | | | | | 0.022 |
| Actinobacteria | 0319-7L14 | 0319-7L14 | 0319-7L14 | unidentified | | | | | | | | 0.015 |
| Actinobacteria | Actinomycetales | Actinomycetaceae | *Actinomyces* | *Actinomyces dentalis* | | | | | | | | 0.031 |
| Actinobacteria | Corynebacteriales | Corynebacteriaceae | *Corynebacterium* | *Corynebacterium durum* | | | | | | | | 0.095 |
| Actinobacteria | Corynebacteriales | Corynebacteriaceae | *Corynebacterium* | *Corynebacterium kroppenstedtii* | | | | | | | | 0.102 |
| Actinobacteria | Corynebacteriales | Corynebacteriaceae | *Corynebacterium* | *Corynebacterium tuberculostearicum* | | | | | | | | 0.183 |
| Actinobacteria | Corynebacteriales | Corynebacteriaceae | *Corynebacterium* | unidentified | | | | | | | | 0.095 |
| Actinobacteria | Corynebacteriales | Mycobacteriaceae | *Mycobacterium* | unidentified | 1.342 | 1.852 | 0.615 | | 1.301 | 3.206 | 1.143 | 0.393 |
| Actinobacteria | Corynebacteriales | Nocardiaceae | *Rhodococcus* | unidentified | | | | | | | 0.06 | |
| Actinobacteria | Euzebyales | Euzebyaceae | uncultured | uncultured_bacterium | | | | | | | | 0.068 |
| Actinobacteria | Frankiales | Geodermatophilaceae | *Blastococcus* | unidentified | | | | | | | 0.286 | 0.06 |
| Actinobacteria | Frankiales | Geodermatophilaceae | *Geodermatophilaceae* | *Blastococcus* sp. | | | | | 0.254 | | | |

| | | | | | | | | | | | | |
|---|---|---|---|---|---|---|---|---|---|---|---|---|
| Actinobacteria | Frankiales | Geodermatophilaceae | *Geodermatophilus* | unidentified | | | | | | | | 0.079 |
| Actinobacteria | Frankiales | Geodermatophilaceae | *Klenkia* | *Klenkia brasiliensis* | | 0.182 | 0.36 | | | | | |
| Actinobacteria | Frankiales | Geodermatophilaceae | *Klenkia* | unidentified | | 0.364 | | | | | | |
| Actinobacteria | Frankiales | Geodermatophilaceae | unidentified | unidentified | | | | | 0.279 | | | |
| Actinobacteria | Frankiales | Sporichthyaceae | uncultured | uncultured_bacterium | | | | | | | | 0.024 |
| Actinobacteria | Kineosporiales | Kineosporiaceae | *Pseudokineococcus* | *Ornithinimicrobium* sp. | | | 0.276 | | | | | |
| Actinobacteria | Kineosporiales | Kineosporiaceae | *Pseudokineococcus* | *Pseudokineococcus lusitanus* | | 0.281 | | | | | | |
| Actinobacteria | Kineosporiales | Kineosporiaceae | *Quadrisphaera* | *Kineosporiaceae bacterium* | | | 0.085 | | | | | |
| Actinobacteria | Kineosporiales | Kineosporiaceae | *Quadrisphaera* | *Quadrisphaera granulorum* | | 0.562 | 0.657 | 0.688 | | | | |
| Actinobacteria | Kineosporiales | Kineosporiaceae | *Quadrisphaera* | uncultured_*Kineococcus* | | 0.165 | | | | | | |
| Actinobacteria | Kineosporiales | Kineosporiaceae | uncultured | uncultured_bacterium | | | | | | | 0.065 | |
| Actinobacteria | Kineosporiales | Kineosporiaceae | unidentified | unidentified | | | | | | | | 0.088 |
| Actinobacteria | Micrococcales | Brevibacteriaceae | *Brevibacterium* | *Brevibacterium paucivorans* | | | | | | | | 0.042 |
| Actinobacteria | Micrococcales | Intrasporangiaceae | *Lapillicoccus* | unidentified | | | | | 0.06 | | | |
| Actinobacteria | Micrococcales | Microbacteriaceae | *Agrococcus* | *Agrococcus terreus* | | | | | | | 0.051 | |
| Actinobacteria | Micrococcales | Microbacteriaceae | *Agromyces* | unidentified | | | | | | 0.134 | 0.203 | |
| Actinobacteria | Micrococcales | Microbacteriaceae | *Curtobacterium* | unidentified | | 0.132 | | | | | | |
| Actinobacteria | Micrococcales | Microbacteriaceae | *Microbacterium* | unidentified | | 0.876 | | | | | | 0.232 |
| Actinobacteria | Micrococcales | Micrococcaceae | *Pseudarthrobacter* | unidentified | | | | | 0.209 | | | |
| Actinobacteria | Micrococcales | Micrococcaceae | *Sinomonas* | uncultured_bacterium | | | | | | | 0.051 | |
| Actinobacteria | Micrococcales | Micrococcaceae | unidentified | unidentified | | | | | 1.226 | | 0.189 | |

| | | | | | | | | | | | | |
|---|---|---|---|---|---|---|---|---|---|---|---|---|
| Actinobacteria | Micromonosporales | Micromonosporaceae | *Actinoplanes* | unidentified | | 0.347 | | | | | | 0.077 |
| Actinobacteria | Micromonosporales | Micromonosporaceae | *Catenuloplanes* | unidentified | | | | | | | | 0.068 |
| Actinobacteria | Micromonosporales | Micromonosporaceae | *Dactylosporangium* | unidentified | | | | | 0.145 | | | |
| Actinobacteria | Micromonosporales | Micromonosporaceae | *Micromonospora* | unidentified | | | | | 0.1 | | | 0.102 |
| Actinobacteria | Micromonosporales | Micromonosporaceae | *Rhizocola* | unidentified | | | | | | | | 0.066 |
| Actinobacteria | Micromonosporales | Micromonosporaceae | unidentified | unidentified | | 0.513 | | | 0.553 | 0.49 | 0.604 | 0.572 |
| Actinobacteria | Micromonosporales | Micromonosporaceae | *Virgisporangium* | unidentified | | | | | | | | 0.214 |
| Actinobacteria | Propionibacteriales | Nocardioidaceae | *Actinopolymorpha* | uncultured_bacterium | | | | | | 0.163 | | |
| Actinobacteria | Propionibacteriales | Nocardioidaceae | *Kribbella* | unidentified | | | | | 0.125 | 0.742 | 0.152 | 0.077 |
| Actinobacteria | Propionibacteriales | Nocardioidaceae | *Marmoricola* | unidentified | | 0.099 | | | | | | 0.04 |
| Actinobacteria | Propionibacteriales | Nocardioidaceae | *Nocardioides* | *Nocardioides kandeliae* | | | | | 0.11 | | 0.092 | |
| Actinobacteria | Propionibacteriales | Nocardioidaceae | *Nocardioides* | *Nocardioides marinquilinus* | | 0.265 | | | | | | |
| Actinobacteria | Propionibacteriales | Nocardioidaceae | *Nocardioides* | unidentified | | 1.918 | 1.06 | | 0.12 | | 0.152 | 0.587 |
| Actinobacteria | Propionibacteriales | Nocardioidaceae | unidentified | unidentified | | | | | | 0.327 | 0.442 | 0.031 |
| Actinobacteria | Propionibacteriales | Propionibacteriaceae | *Cutibacterium* | unidentified | | | | | | | | 0.911 |
| Actinobacteria | Propionibacteriales | Propionibacteriaceae | *Friedmanniella* | unidentified | | | | 0.129 | | | | |
| Actinobacteria | Propionibacteriales | Propionibacteriaceae | *Microlunatus* | uncultured_actinobacterium | | | | | 1.506 | | 4.041 | 0.675 |
| Actinobacteria | Propionibacteriales | Propionibacteriaceae | *Microlunatus* | unidentified | | | | | 1.341 | 0.193 | 1.77 | 0.313 |

| | | | | | | | | | | | | |
|---|---|---|---|---|---|---|---|---|---|---|---|---|
| Actinobacteria | Pseudonocardiales | Pseudonocardiaceae | *Actinomycetospora* | uncultured_bacterium | 6.711 | 3.043 | 4.388 | | | | | |
| Actinobacteria | Pseudonocardiales | Pseudonocardiaceae | *Actinomycetospora* | unidentified | 13.231 | 4.829 | 12.317 | 7.134 | | | | |
| Actinobacteria | Pseudonocardiales | Pseudonocardiaceae | *Actinophytocola* | unidentified | | | | | | 0.371 | 0.175 | |
| Actinobacteria | Pseudonocardiales | Pseudonocardiaceae | *Amycolatopsis* | unidentified | | | | | | | | 0.046 |
| Actinobacteria | Pseudonocardiales | Pseudonocardiaceae | *Crossiella* | unidentified | | | | | 0.194 | 0.653 | | 0.154 |
| Actinobacteria | Pseudonocardiales | Pseudonocardiaceae | *Kibdelosporangium* | *Kibdelosporangium* sp. | | | | | 0.145 | | | |
| Actinobacteria | Pseudonocardiales | Pseudonocardiaceae | *Kibdelosporangium* | unidentified | | | | | | | 0.194 | |
| Actinobacteria | Pseudonocardiales | Pseudonocardiaceae | *Lechevalieria* | unidentified | | | | | | | | 0.068 |
| Actinobacteria | Pseudonocardiales | Pseudonocardiaceae | *Pseudonocardia* | unidentified | | 2.001 | 2.65 | 1.418 | 0.005 | | 0.544 | 0.166 |
| Actinobacteria | Pseudonocardiales | Pseudonocardiaceae | *Saccharopolyspora* | *Saccharopolyspora cebuensis* | | | | | | | | 0.06 |
| Actinobacteria | Pseudonocardiales | Pseudonocardiaceae | unidentified | unidentified | | | | | 0.244 | | | 0.06 |
| Actinobacteria | Streptomycetales | Streptomycetaceae | *Streptomyces* | *Streptomyces fractus* | | | | | | 0.401 | | |
| Actinobacteria | Streptomycetales | Streptomycetaceae | *Streptomyces* | unidentified | | | | | 0.528 | 4.141 | 0.378 | 0.563 |
| Actinobacteria | Streptosporangiales | Streptosporangiaceae | *Nonomuraea* | unidentified | | | | | 0.11 | | | |
| Actinobacteria | Streptosporangiales | Streptosporangiaceae | unidentified | unidentified | | | | | | | 0.074 | |
| Actinobacteria | Streptosporangiales | Thermomonosporaceae | *Actinoallomurus* | unidentified | | | | | | | | 0.024 |
| Actinobacteria | Streptosporangiales | unidentified | unidentified | unidentified | | | | | | | | 0.046 |
| Actinobacteria | unidentified | unidentified | unidentified | unidentified | | | | | 0.259 | 0.163 | 1.295 | 0.474 |
| MB-A2-108 | MB-A2-108 | MB-A2-108 | MB-A2-108 | uncultured_bacterium | | | | | | 0.015 | 0.419 | 0.15 |

| | | | | | | | | | | | | |
|---|---|---|---|---|---|---|---|---|---|---|---|---|
| MB-A2-108 | MB-A2-108 | MB-A2-108 | MB-A2-108 | unidentified | | | | | 0.723 | 0.237 | 1.369 | 0.331 |
| Rubrobacteria | Rubrobacterales | Rubrobacteriaceae | *Rubrobacter* | metagenome | | | | | 0.957 | 0.312 | 2.493 | 0.64 |
| Rubrobacteria | Rubrobacterales | Rubrobacteriaceae | *Rubrobacter* | uncultured_actinobacterium | | | | | | | | 0.044 |
| Rubrobacteria | Rubrobacterales | Rubrobacteriaceae | *Rubrobacter* | uncultured_*Actinomycetales* | | | | | 0.917 | | 0.641 | 0.327 |
| Rubrobacteria | Rubrobacterales | Rubrobacteriaceae | *Rubrobacter* | uncultured_*Rubrobacter* | | | | | 0.289 | | | |
| Rubrobacteria | Rubrobacterales | Rubrobacteriaceae | *Rubrobacter* | unidentified | | | | | 1.391 | | 1.29 | 0.291 |
| Thermoleophilia | Gaiellales | Gaiellaceae | *Gaiella* | uncultured_*Acidimicrobiia* | | | | | 0.16 | | 0.078 | 0.04 |
| Thermoleophilia | Gaiellales | Gaiellaceae | *Gaiella* | uncultured_bacterium | | | | | 0.508 | | 0.3 | 0.777 |
| Thermoleophilia | Gaiellales | Gaiellaceae | *Gaiella* | uncultured_organism | | | | | | | 0.032 | 0.121 |
| Thermoleophilia | Gaiellales | Gaiellaceae | *Gaiella* | uncultured_*Rubrobacteria* | | | | | 0.209 | | | 0.084 |
| Thermoleophilia | Gaiellales | Gaiellaceae | *Gaiella* | unidentified | | | | | | | 0.493 | 1.479 |
| Thermoleophilia | Gaiellales | uncultured | uncultured | uncultured_actinobacterium | | | | | 0.08 | | 0.336 | 1.675 |
| Thermoleophilia | Gaiellales | uncultured | uncultured | uncultured_bacterium | | | | | | | 0.336 | 0.342 |
| Thermoleophilia | Gaiellales | uncultured | uncultured | uncultured_organism | | | | | | | 0.041 | |
| Thermoleophilia | Gaiellales | uncultured | uncultured | uncultured_*Rubrobacterales* | | | | | 0.264 | 0.178 | | 0.031 |
| Thermoleophilia | Gaiellales | uncultured | uncultured | unidentified | | | | | 1.535 | | 2.355 | 6.298 |
| Thermoleophilia | Gaiellales | unidentified | unidentified | unidentified | | | | | 0.339 | | 0.438 | 2.377 |
| Thermoleophilia | Solirubrobacterales | 67-14 | 67-14 | uncultured_bacterium | | | | | 0.065 | | | 1.461 |
| Thermoleophilia | Solirubrobacterales | 67-14 | 67-14 | uncultured_*Conexibacteraceae* | | | | | | | | 0.026 |

| | | | | | | | | | | | | |
|---|---|---|---|---|---|---|---|---|---|---|---|---|
| Thermoleophilia | Solirubrobacterales | 67-14 | 67-14 | uncultured_*Rubrobacteraceae* | | | | | 0.12 | 0.505 | | |
| Thermoleophilia | Solirubrobacterales | 67-14 | 67-14 | uncultured_*Rubrobacteria* | | | | | | | 0.069 | |
| Thermoleophilia | Solirubrobacterales | 67-14 | 67-14 | unidentified | | | | | 2.014 | 0.119 | 1.76 | 2.357 |
| Thermoleophilia | Solirubrobacterales | Solirubrobacteraceae | *Conexibacter* | unidentified | | | | | | | | 0.064 |
| Thermoleophilia | Solirubrobacterales | Solirubrobacteraceae | *Solirubrobacter* | uncultured_bacterium | | | | | 0.409 | 0.401 | 0.272 | 0.265 |
| Thermoleophilia | Solirubrobacterales | Solirubrobacteraceae | *Solirubrobacter* | unidentified | | | | | 0.309 | | 0.332 | 0.093 |
| Thermoleophilia | Solirubrobacterales | Solirubrobacteraceae | unidentified | unidentified | | 0.628 | 0.466 | 1.031 | | | 0.249 | |
| Thermoleophilia | Solirubrobacterales | unidentified | unidentified | unidentified | | | | | 0.773 | 1.187 | 0.581 | 0.225 |
| Thermoleophilia | uncultured | uncultured | uncultured | uncultured_bacterium | | | | | | | | 0.075 |
| Thermoleophilia | unidentified | unidentified | unidentified | unidentified | | | | | 0.005 | | 0.065 | 0.09 |
| unidentified | unidentified | unidentified | unidentified | unidentified | | | | | 0.633 | | 0.124 | 0.088 |
| **Armatimonadota** | | | | | | | | | | | | |
| Armatimonadia | Armatimonadales | Armatimonadales | *Armatimonadales* | unidentified | | 0.033 | | | | | | |
| **Bacteroidota** | | | | | | | | | | | | |
| Bacteroidia | Bacteroidales | Bacteroidaceae | *Bacteroides* | unidentified | | | | | | | | 0.02 |
| Bacteroidia | Bacteroidales | Bacteroidetes_vadinHA17 | *Bacteroidetes_vadinHA17* | unidentified | | | | | | | | 0.024 |
| Bacteroidia | Chitinophagales | Chitinophagaceae | *Aurantisolimonas* | uncultured_bacterium | | 0.165 | | | | | | |
| Bacteroidia | Chitinophagales | Chitinophagaceae | *Chitinophaga* | *Chitinophaga filiformis* | | | | | | | 0.083 | |
| Bacteroidia | Chitinophagales | Chitinophagaceae | *Chitinophaga* | *Chitinophaga ginsengihumi* | | | | | | | 0.078 | |

| | | | | | | | | | | | | |
|---|---|---|---|---|---|---|---|---|---|---|---|---|
| Bacteroidia | Chitinophagales | Chitinophagaceae | *Cnuella* | uncultured_bacterium | | | | | | | | 0.035 |
| Bacteroidia | Chitinophagales | Chitinophagaceae | *Dinghuibacter* | uncultured_bacterium | | | | | | | 0.032 | |
| Bacteroidia | Chitinophagales | Chitinophagaceae | *Edaphobaculum* | uncultured_bacterium | | 0.099 | | | | | | |
| Bacteroidia | Chitinophagales | Chitinophagaceae | *Edaphobaculum* | uncultured_*Chitinophaga* | | 0.116 | 0.085 | | | | | |
| Bacteroidia | Chitinophagales | Chitinophagaceae | *Edaphobaculum* | unidentified | | | | | | | | 0.024 |
| Bacteroidia | Chitinophagales | Chitinophagaceae | *Flavisolibacter* | *Flavisolibacter swuensis* | | | | | | | | 0.042 |
| Bacteroidia | Chitinophagales | Chitinophagaceae | *Flavisolibacter* | *Flavisolibacter tropicus* | | | | | 0.06 | | | |
| Bacteroidia | Chitinophagales | Chitinophagaceae | *Flavisolibacter* | unidentified | | 0.232 | 0.254 | | | | | 0.232 |
| Bacteroidia | Chitinophagales | Chitinophagaceae | *Niastella* | uncultured_soil | | | | | | | 0.083 | |
| Bacteroidia | Chitinophagales | Chitinophagaceae | *Niastella* | unidentified | | | | | 0.184 | | 0.249 | |
| Bacteroidia | Chitinophagales | Chitinophagaceae | *Puia* | unidentified | | | | | | | 0.065 | |
| Bacteroidia | Chitinophagales | Chitinophagaceae | *Terrimonas* | unidentified | | | | | 0.13 | | 0.175 | |
| Bacteroidia | Chitinophagales | Chitinophagaceae | uncultured | *Panacibacter ginsenosidivorans* | | | | | 0.035 | | | |
| Bacteroidia | Chitinophagales | Chitinophagaceae | uncultured | uncultured_*Flavisolibacter* | | | | | 0.05 | | | |
| Bacteroidia | Chitinophagales | Chitinophagaceae | uncultured | uncultured_*Niastella* | | | | | | | | 0.068 |
| Bacteroidia | Chitinophagales | Chitinophagaceae | uncultured | unidentified | | | | | 0.045 | | 0.198 | 0.042 |
| Bacteroidia | Chitinophagales | Chitinophagaceae | unidentified | unidentified | | | | | | | 0.014 | 0.099 |
| Bacteroidia | Chitinophagales | Saprospiraceae | uncultured | unidentified | | | | | 0.015 | | | |
| Bacteroidia | Chitinophagales | unidentified | unidentified | unidentified | | | | | | | | 0.024 |
| Bacteroidia | Cytophagales | Cyclobacteriaceae | uncultured | unidentified | | | | | | | | 0.035 |
| Bacteroidia | Cytophagales | Hymenobacteraceae | *Hymenobacter* | unidentified | | 0.083 | 0.106 | | | | | 0.132 |
| Bacteroidia | Cytophagales | Microscillaceae | *Chryseolinea* | uncultured_bacterium | | | | | | | 0.06 | |

| | | | | | | | | | | | | |
|---|---|---|---|---|---|---|---|---|---|---|---|---|
| Bacteroidia | Cytophagales | Microscillaceae | *Chryseolinea* | unidentified | | | | | | | 0.078 | |
| Bacteroidia | Cytophagales | Microscillaceae | *Flexibacter* | unidentified | | | | | | | 0.023 | |
| Bacteroidia | Cytophagales | Microscillaceae | *Ohtaekwangia* | unidentified | | | | | | | | 0.022 |
| Bacteroidia | Cytophagales | Microscillaceae | uncultured | uncultured_soil | | | | | | | | 0.108 |
| Bacteroidia | Cytophagales | Microscillaceae | uncultured | unidentified | | | | | | | 0.134 | |
| Bacteroidia | Cytophagales | Microscillaceae | unidentified | unidentified | | | | | | | 0.06 | |
| Bacteroidia | Cytophagales | Spirosomaceae | *Dyadobacter* | unidentified | | 0.248 | | | | | 0.078 | |
| Bacteroidia | Cytophagales | Spirosomaceae | *Larkinella* | *Larkinella harenae* | | 0.298 | 0.254 | | | | | |
| Bacteroidia | Cytophagales | Spirosomaceae | *Larkinella* | *Larkinella insperata* | | | 0.148 | | | | | |
| Bacteroidia | Cytophagales | Spirosomaceae | *Larkinella* | *Larkinella ripae* | | 0.083 | 0.106 | | | | 0.014 | |
| Bacteroidia | Cytophagales | Spirosomaceae | *Rudanella* | uncultured_bacterium | | 0.165 | | 0.387 | | | | |
| Bacteroidia | Cytophagales | Spirosomaceae | *Spirosoma* | *Spirosoma oryzae* | | 0.48 | | 0.43 | | | | |
| Bacteroidia | Cytophagales | Spirosomaceae | *Spirosoma* | unidentified | | 0.232 | 0.466 | | | | | |
| Bacteroidia | Flavobacteriales | Flavobacteriaceae | *Flavobacterium* | unidentified | | | | | | | 0.041 | |
| Bacteroidia | Flavobacteriales | NS9_marine_group | NS9_marine_group | metagenome | | | | | 0.015 | | | |
| Bacteroidia | Flavobacteriales | unidentified | unidentified | unidentified | | | | | | | | 0.066 |
| Bacteroidia | Flavobacteriales | Weeksellaceae | *Chryseobacterium* | unidentified | | 0.099 | | | | | | |
| Bacteroidia | Sphingobacteriales | AKYH767 | AKYH767 | uncultured_bacterium | | | | | | | 0.014 | |
| Bacteroidia | Sphingobacteriales | env.OPS_17 | env.OPS_17 | unidentified | | | | | 0.035 | | | 0.057 |
| Bacteroidia | Sphingobacteriales | Sphingobacteriaceae | *Mucilaginibacter* | unidentified | | | | | | | 0.032 | |
| Bacteroidia | Sphingobacteriales | unidentified | unidentified | unidentified | | | | | | | | 0.009 |
| Kapabacteria | Kapabacteriales | Kapabacteriales | *Kapabacteriales* | unidentified | | | | | | | 0.028 | |
| SJA-28 | SJA-28 | SJA-28 | SJA-28 | metagenome | | | | | 0.02 | | | |
| SJA-28 | SJA-28 | SJA-28 | SJA-28 | uncultured_bacterium | | | | | | | | 0.093 |

| | | | | | | | | | | | | |
|---|---|---|---|---|---|---|---|---|---|---|---|---|
| **Bdellovibrionota** | | | | | | | | | | | | |
| Bdellovibrionia | Bdellovibrionales | Bdellovibrionaceae | *Bdellovibrio* | uncultured_bacterium | | | | | | | | 0.051 |
| Bdellovibrionia | Bdellovibrionales | Bdellovibrionaceae | OM27_clade | uncultured_*Myxococcales* | | | | | | | 0.092 | |
| Bdellovibrionia | Bdellovibrionales | Bdellovibrionaceae | OM27_clade | unidentified | | | | | | | | 0.11 |
| Oligoflexia | Oligoflexales | uncultured | uncultured | unidentified | | | | | | | | 0.04 |
| **Chloroflexi** | | | | | | | | | | | | |
| Anaerolineae | Anaerolineales | Anaerolineaceae | uncultured | unidentified | | | | | 0.035 | | | 0.042 |
| Anaerolineae | Anaerolineales | Anaerolineaceae | UTCFX1 | uncultured_bacterium | | | | | | | | 0.042 |
| Anaerolineae | Ardenticatenales | Ardenticatenaceae | uncultured | unidentified | | | | | | 0.148 | | |
| Anaerolineae | Ardenticatenales | uncultured | uncultured | uncultured_bacterium | | | | | 0.02 | | 0.051 | |
| Anaerolineae | Ardenticatenales | uncultured | uncultured | uncultured_*Litorilinea* | | | | | | | 0.069 | |
| Anaerolineae | Ardenticatenales | uncultured | uncultured | unidentified | | | | | | | 0.014 | |
| Anaerolineae | Caldilineales | Caldilineaceae | *Litorilinea* | uncultured_bacterium | | | | | | | | 0.057 |
| Anaerolineae | Caldilineales | Caldilineaceae | uncultured | uncultured_bacterium | | | | | | | | 0.071 |
| Anaerolineae | Caldilineales | Caldilineaceae | uncultured | uncultured_soil | | | | | 0.05 | | | |
| Anaerolineae | Caldilineales | Caldilineaceae | uncultured | unidentified | | | | | | | 0.032 | |
| Anaerolineae | RBG-13-54-9 | RBG-13-54-9 | RBG-13-54-9 | unidentified | | | | | | | 0.014 | |
| Anaerolineae | SBR1031 | A4b | A4b | uncultured_bacterium | | | | | 0.055 | | 0.253 | 0.426 |
| Anaerolineae | SBR1031 | A4b | A4b | uncultured_*Caldilineales* | | | | | | | | 0.095 |
| Anaerolineae | SBR1031 | A4b | A4b | uncultured_soil | | | | | | | 0.046 | |
| Anaerolineae | SBR1031 | A4b | A4b | unidentified | | | | | 0.065 | 0.134 | 0.028 | 0.247 |
| Anaerolineae | SBR1031 | SBR1031 | SBR1031 | uncultured_bacterium | | | | | | | | 0.055 |

| | | | | | | | | | | | | |
|---|---|---|---|---|---|---|---|---|---|---|---|---|
| Anaerolineae | SBR1031 | SBR1031 | SBR1031 | uncultured_*Caldilineaceae* | | | | | | | 0.014 | |
| Anaerolineae | SBR1031 | SBR1031 | SBR1031 | uncultured_soil | | | | | | | | 0.082 |
| Anaerolineae | SBR1031 | SBR1031 | SBR1031 | unidentified | | | | | | | | 0.106 |
| Anaerolineae | SBR1031 | unidentified | unidentified | unidentified | | | | | | | 0.051 | 0.174 |
| Chloroflexia | Chloroflexales | Roseiflexaceae | uncultured | uncultured_bacterium | 0.288 | 0.281 | 0.996 | 0.43 | 0.314 | | 0.152 | 0.185 |
| Chloroflexia | Chloroflexales | Roseiflexaceae | uncultured | uncultured_*Chloroflexi* | | | | | | | 0.129 | |
| Chloroflexia | Thermomicrobiales | JG30-KF-CM45 | JG30-KF-CM45 | uncultured_bacterium | 0.959 | 1.472 | 1.208 | 0.258 | 0.025 | | | 0.177 |
| Chloroflexia | Thermomicrobiales | JG30-KF-CM45 | JG30-KF-CM45 | unidentified | | | | | | | | 0.073 |
| Chloroflexia | Thermomicrobiales | Thermomicrobiaceae | *Nitrolancea* | *Nitrolancea hollandica* | | | | | | | | 0.053 |
| Dehalococcoidia | S085 | S085 | S085 | metagenome | | | | | | | | 0.038 |
| Dehalococcoidia | S085 | S085 | S085 | uncultured_bacterium | | | | | 0.055 | | 0.392 | 0.108 |
| Dehalococcoidia | S085 | S085 | S085 | uncultured_*Chloroflexi* | | | | | | | | 0.053 |
| Dehalococcoidia | S085 | S085 | S085 | unidentified | | | | | | | 0.069 | 0.373 |
| Dehalococcoidia | SAR202_clade | SAR202_clade | SAR202_clade | uncultured_soil | | | | | | | 0.023 | |
| Gitt-GS-136 | Gitt-GS-136 | Gitt-GS-136 | Gitt-GS-136 | uncultured_bacterium | | | | | 0.065 | | 0.484 | 0.135 |
| Gitt-GS-136 | Gitt-GS-136 | Gitt-GS-136 | Gitt-GS-136 | unidentified | | | | | | | 0.17 | |
| JG30-KF-CM66 | JG30-KF-CM66 | JG30-KF-CM66 | JG30-KF-CM66 | uncultured_bacterium | | | | | | | | 0.06 |
| KD4-96 | KD4-96 | KD4-96 | KD4-96 | metagenome | | | | | | 0.341 | | 0.104 |
| KD4-96 | KD4-96 | KD4-96 | KD4-96 | uncultured_bacterium | | | | | | | 0.465 | 0.113 |
| KD4-96 | KD4-96 | KD4-96 | KD4-96 | uncultured_*Chloroflexi* | | | | | | 0.579 | 0.06 | |

| | | | | | | | | | | | | |
|---|---|---|---|---|---|---|---|---|---|---|---|---|
| KD4-96 | KD4-96 | KD4-96 | KD4-96 | uncultured_*Levilinea* | | | | | | | | 0.108 |
| KD4-96 | KD4-96 | KD4-96 | KD4-96 | unidentified | | | | | 0.454 | 0.267 | 0.576 | 0.185 |
| Ktedonobacteria | C0119 | C0119 | C0119 | uncultured_bacterium | | | | | | | 0.023 | |
| Ktedonobacteria | Ktedonobacterales | Ktedonobacteraceae | uncultured | unidentified | | | | | | | 0.037 | |
| OLB14 | OLB14 | OLB14 | OLB14 | uncultured_bacterium | | | | | | | 0.051 | |
| P2-11E | P2-11E | P2-11E | P2-11E | uncultured_bacterium | | | | | | | 0.152 | 0.018 |
| TK10 | TK10 | TK10 | TK10 | bacterium_Ellin | | | | | | 0.208 | | |
| TK10 | TK10 | TK10 | TK10 | uncultured_bacterium | | | | | 0.13 | | 0.097 | 0.117 |
| TK10 | TK10 | TK10 | TK10 | uncultured_*Chloroflexi* | | | | | 0.065 | | | 0.035 |
| TK10 | TK10 | TK10 | TK10 | uncultured *Firmicutes* | | | | | 0.14 | | | |
| TK10 | TK10 | TK10 | TK10 | unidentified | | | | | 0.135 | | 0.244 | 0.161 |
| **Cyanobacteria** | | | | | | | | | | | | |
| Cyanobacteriia | Cyanobacteriales | Chroococcidiopsaceae | unidentified | unidentified | | | | | | | | 0.227 |
| Cyanobacteriia | Cyanobacteriales | Nostocaceae | *Scytonema_VB-61278* | unidentified | 1.726 | | 0.339 | | | | | |
| Cyanobacteriia | Cyanobacteriales | unidentified | unidentified | unidentified | | | | | | | | 0.141 |
| Sericytochromatia | Sericytochromatia | Sericytochromatia | *Sericytochromatia* | uncultured bacterium | | | | | | | | 0.009 |
| Vampirivibrionia | Obscuribacterales | Obscuribacteraceae | *Obscuribacteraceae* | bioreactor metagenome | | | | | | | 0.032 | |
| Vampirivibrionia | Obscuribacterales | Obscuribacteraceae | *Obscuribacteraceae* | uncultured_bacterium | | | | | | | | 0.238 |
| Vampirivibrionia | Vampirovibrionales | Vampirovibrionales | *Vampirovibrionales* | uncultured_bacterium | | | | | | | | 0.093 |
| **Deinococcota** | | | | | | | | | | | | |

| | | | | | | | | | | | | |
|---|---|---|---|---|---|---|---|---|---|---|---|---|
| Deinococci | Deinococcales | Deinococcaceae | *Deinococcus* | *Deinococcus cellulosilyticus* | | | | | | | | 0.053 |
| Deinococci | Deinococcales | Deinococcaceae | *Deinococcus* | unidentified | | | | | | | | 0.115 |
| Deinococci | Deinococcales | Trueperaceae | *Truepera* | uncultured_*Deinococcus* | | | 0.276 | | | | | |
| Deinococci | Deinococcales | Trueperaceae | *Truepera* | unidentified | | 0.281 | 0.339 | | | | | |
| **Dependentiae** | | | | | | | | | | | | |
| Babeliae | Babeliales | unidentified | unidentified | unidentified | | | | | | | | 0.068 |
| Babeliae | Babeliales | Vermiphilaceae | *Vermiphilaceae* | uncultured_bacterium | | | | | | | | 0.053 |
| Babeliae | Babeliales | Vermiphilaceae | *Vermiphilaceae* | unidentified | | | | | | | | 0.024 |
| **Desulfobacterota** | | | | | | | | | | | | |
| Desulfuromonadia | Geobacterales | Geobacteraceae | *Citrifermentans* | unidentified | | | | | | | | 0.046 |
| Desulfuromonadia | Geobacterales | Geobacteraceae | *Geobacter* | unidentified | | | | | | | | 0.013 |
| Desulfuromonadia | Geobacterales | Geobacteraceae | unidentified | unidentified | | | | | | | | 0.084 |
| Syntrophobacteria | Syntrophobacterales | Syntrophobacteraceae | *Syntrophobacter* | unidentified | | | | | | | | 0.031 |
| uncultured | uncultured | uncultured | uncultured | uncultured_*Dongia* | | | | | | | | 0.051 |
| uncultured | uncultured | uncultured | uncultured | unidentified | | | | | 0.12 | | 0.152 | 0.159 |
| **Elusimicrobiota** | | | | | | | | | | | | |
| Elusimicrobia | MVP-88 | MVP-88 | MVP-88 | uncultured_bacterium | | | | | | | | 0.11 |
| Lineage_IIb | Lineage_IIb | Lineage_IIb | *Lineage_IIb* | uncultured bacterium | | | | | 0.015 | | | |
| **Entotheonellaeota** | | | | | | | | | | | | |
| Entotheonellia | Entotheonellales | Entotheonellaceae | *Entotheonellaceae* | uncultured bacterium | | | | | 0.065 | | 0.221 | 0.132 |
| Entotheonellia | Entotheonellales | Entotheonellaceae | *Entotheonellaceae* | unidentified | | | | | 0.753 | | | 0.055 |

| | | | | | | | | | | | | |
|---|---|---|---|---|---|---|---|---|---|---|---|---|
| **Firmicutes** | | | | | | | | | | | | |
| Bacilli | Bacillales | Bacillaceae | *Bacillus* | *Bacillus acidiceler* | | | | | 1.092 | 1.633 | | |
| Bacilli | Bacillales | Bacillaceae | *Bacillus* | *Bacillus arbutinivorans* | | | | | 1.396 | 5.387 | | |
| Bacilli | Bacillales | Bacillaceae | *Bacillus* | *Bacillus asahii* | | | | | 0.045 | 1.306 | | 0.071 |
| Bacilli | Bacillales | Bacillaceae | *Bacillus* | *Bacillus fastidiosus* | | | | | | | 0.023 | |
| Bacilli | Bacillales | Bacillaceae | *Bacillus* | *Bacillus fengqiuensis* | | | | | 0.508 | | 0.723 | 0.104 |
| Bacilli | Bacillales | Bacillaceae | *Bacillus* | *Bacillus funiculus* | | | | | 2.258 | | 0.309 | |
| Bacilli | Bacillales | Bacillaceae | *Bacillus* | *Bacillus idriensis* | | | | | | 0.89 | | |
| Bacilli | Bacillales | Bacillaceae | *Bacillus* | *Bacillus korlensis* | | | | | 0.08 | | | |
| Bacilli | Bacillales | Bacillaceae | *Bacillus* | *Bacillus malikii* | | | | | 0.309 | | | |
| Bacilli | Bacillales | Bacillaceae | *Bacillus* | *Bacillus nealsonii* | | | | | 0.623 | 1.469 | | |
| Bacilli | Bacillales | Bacillaceae | *Bacillus* | *Bacillus niacini* | | | | | 1.226 | | | |
| Bacilli | Bacillales | Bacillaceae | *Bacillus* | uncultured_organism | | | | | | | 0.065 | 0.104 |
| Bacilli | Bacillales | Bacillaceae | *Bacillus* | unidentified | | | | | 24.892 | 28.317 | 2.696 | 0.653 |
| Bacilli | Bacillales | Bacillaceae | *Fictibacillus* | unidentified | | | | | | 1.232 | | |
| Bacilli | Bacillales | Bacillaceae | unidentified | unidentified | | | | | | | | 0.055 |
| Bacilli | Bacillales | Bacillaceae | *Virgibacillus* | uncultured_bacterium | | | | | | | | 0.04 |
| Bacilli | Bacillales | Planococcaceae | *Chungangia* | unidentified | | | | | 0.015 | | | |
| Bacilli | Bacillales | Planococcaceae | *Domibacillus* | uncultured_*Bacillus* | | | | | 1.665 | 1.187 | | |
| Bacilli | Bacillales | Planococcaceae | *Domibacillus* | uncultured_bacterium | | | | | 0.055 | | | |
| Bacilli | Bacillales | Planococcaceae | *Domibacillus* | unidentified | | | | | 0.862 | 0.505 | 0.032 | |
| Bacilli | Bacillales | Planococcaceae | *Lysinibacillus* | *Lysinibacillus composti* | | | | | 0.075 | | | |
| Bacilli | Bacillales | Planococcaceae | *Lysinibacillus* | uncultured_bacterium | | | | | | | 0.037 | |
| Bacilli | Bacillales | unidentified | unidentified | unidentified | | | | | 0.165 | 0.564 | | 0.119 |

| | | | | | | | | | | | | |
|---|---|---|---|---|---|---|---|---|---|---|---|---|
| Bacilli | Brevibacillales | Brevibacillaceae | *Brevibacillus* | unidentified | | | | | 0.045 | | | 0.115 |
| Bacilli | Lactobacillales | Streptococcaceae | *Streptococcus* | *Streptococcus agalactiae* | | | | | | | | 1.079 |
| Bacilli | Lactobacillales | Streptococcaceae | *Streptococcus* | uncultured_organism | | | | | | | | 0.108 |
| Bacilli | Paenibacillales | Paenibacillaceae | *Ammoniphilus* | unidentified | | | | | 0.803 | | 0.166 | |
| Bacilli | Paenibacillales | Paenibacillaceae | *Paenibacillus* | *Paenibacillus cellulosilyticus* | | | | | | | | 0.071 |
| Bacilli | Paenibacillales | Paenibacillaceae | *Paenibacillus* | *Paenibacillus pocheonensis* | | | | | 0.095 | | | |
| Bacilli | Paenibacillales | Paenibacillaceae | *Paenibacillus* | *Paenibacillus zeae* | | | | | | | | 0.062 |
| Bacilli | Paenibacillales | Paenibacillaceae | *Paenibacillus* | unidentified | | | | | 0.319 | 0.519 | | 0.031 |
| Bacilli | Staphylococcales | Staphylococcaceae | *Staphylococcus* | unidentified | | | | | | | | 0.647 |
| Clostridia | Peptostreptococcales-Tissierellales | Peptostreptococcales-Tissierellales | *Finegoldia* | *Finegoldia magna* | | | | | | | | 0.075 |
| **GAL15** | | | | | | | | | | | | |
| GAL15 | GAL15 | GAL15 | GAL15 | uncultured_bacterium | | | | | 0.025 | | | |
| **Gemmatimonadota** | | | | | | | | | | | | |
| AKAU4049 | AKAU4049 | AKAU4049 | AKAU4049 | uncultured_bacterium | | | | | | | 0.041 | |
| AKAU4049 | AKAU4049 | AKAU4049 | AKAU4049 | uncultured_soil | | | | | | | 0.065 | 0.026 |
| BD2-11_terrestrial_group | BD2-11_terrestrial_group | BD2-11_terrestrial_group | BD2-11_terrestrial_group | metagenome | | | | | | | 0.088 | |
| BD2-11_terrestrial_group | BD2-11_terrestrial_group | BD2-11_terrestrial_group | BD2-11_terrestrial_group | uncultured_soil | | | | | | | | 0.15 |
| Gemmatimonadetes | Gemmatimonadales | Gemmatimonadaceae | *Gemmatimonas* | unidentified | | | | | 0.065 | | | 0.238 |
| Gemmatimonadetes | Gemmatimonadales | Gemmatimonadaceae | *Roseisolibacter* | uncultured_bacterium | | | | | | | | 0.084 |

| | | | | | | | | | | | | |
|---|---|---|---|---|---|---|---|---|---|---|---|---|
| Gemmatimonadetes | Gemmatimonadales | Gemmatimonadaceae | *Roseisolibacter* | unidentified | | | | | 0.075 | | | |
| Gemmatimonadetes | Gemmatimonadales | Gemmatimonadaceae | uncultured | uncultured_proteobacterium | | | | | 0.234 | | | 0.066 |
| Gemmatimonadetes | Gemmatimonadales | Gemmatimonadaceae | uncultured | unidentified | | | | | 0.269 | | 1.106 | 0.863 |
| Gemmatimonadetes | Gemmatimonadales | Gemmatimonadaceae | unidentified | unidentified | | | | | 0.494 | | 0.65 | 0.353 |
| Longimicrobia | Longimicrobiales | Longimicrobiaceae | *Longimicrobiaceae* | uncultured_bacterium | | | | | | | | 0.13 |
| Longimicrobia | Longimicrobiales | Longimicrobiaceae | *Longimicrobiaceae* | uncultured_soil | | | | | | | | 0.055 |
| Longimicrobia | Longimicrobiales | Longimicrobiaceae | *Longimicrobium* | uncultured_bacterium | | | 0.064 | | | | | |
| Longimicrobia | Longimicrobiales | Longimicrobiaceae | YC-ZSS-LKJ147 | uncultured_bacterium | | | | | | | | 0.026 |
| Longimicrobia | Longimicrobiales | Longimicrobiaceae | YC-ZSS-LKJ147 | unidentified | | | | | | | | 0.02 |
| S0134_terrestrial_group | S0134_terrestrial_group | S0134_terrestrial_group | S0134_terrestrial_group | uncultured_bacterium | | | | | 0.025 | | 0.005 | 0.064 |
| S0134_terrestrial_group | S0134_terrestrial_group | S0134_terrestrial_group | S0134_terrestrial_group | uncultured_*Gemmatimonadales* | | | | | 0.055 | | | 0.053 |
| S0134_terrestrial_group | S0134_terrestrial_group | S0134_terrestrial_group | S0134_terrestrial_group | uncultured_*Gemmatimonadetes* | | | | | | | 0.134 | 0.062 |
| S0134_terrestrial_group | S0134_terrestrial_group | S0134_terrestrial_group | S0134_terrestrial_group | unidentified | | | | | 0.095 | | 0.092 | 0.04 |
| **Latescibacterota** | | | | | | | | | | | | |
| Latescibacteria | Latescibacterales | Latescibacteraceae | *Latescibacteraceae* | uncultured_bacterium | | | | | | | 0.046 | |
| Latescibacterota | Latescibacterota | Latescibacterota | *Latescibacterota* | uncultured_bacterium | | | | | | | 0.134 | |
| Latescibacterota | Latescibacterota | Latescibacterota | *Latescibacterota* | uncultured_soil | | | | | | | 0.138 | |
| Latescibacterota | Latescibacterota | Latescibacterota | *Latescibacterota* | unidentified | | | | | | 0.119 | 0.207 | 0.117 |

| | | | | | | | | | | | | |
|---|---|---|---|---|---|---|---|---|---|---|---|---|
| **MBNT15** | | | | | | | | | | | | |
| MBNT15 | MBNT15 | MBNT15 | MBNT15 | uncultured_bacterium | | | | | | | 0.12 | |
| MBNT15 | MBNT15 | MBNT15 | MBNT15 | uncultured_*Myxococcales* | | | | | 0.045 | | | |
| MBNT15 | MBNT15 | MBNT15 | MBNT15 | unidentified | | | | | | | | 0.051 |
| **Methylomirabilota** | | | | | | | | | | | | |
| Methylomirabilia | Rokubacteriales | Rokubacteriales | *Rokubacteriales* | metagenome | | | | | 0.155 | | | 0.013 |
| Methylomirabilia | Rokubacteriales | Rokubacteriales | *Rokubacteriales* | uncultured_bacterium | | | | | 0.698 | | 1.364 | 0.814 |
| Methylomirabilia | Rokubacteriales | WX65 | WX65 | uncultured_bacterium | | | | | | | | 0.117 |
| **Myxococcota** | | | | | | | | | | | | |
| bacteriap25 | bacteriap25 | bacteriap25 | bacteriap25 | uncultured_*Syntrophobacterales* | | | | | | | 0.078 | |
| bacteriap25 | bacteriap25 | bacteriap25 | bacteriap25 | unidentified | | | | | 0.972 | 0.208 | 0.977 | 0.333 |
| bacteriap25 | bacteriap25 | bacteriap25 | bacteriap25 | unidentified | | | | | | | | 0.046 |
| Myxococcia | Myxococcales | Anaeromyxobacteraceae | *Anaeromyxobacter* | uncultured_*Anaeromyxobacter* | | | | | | | | 0.022 |
| Myxococcia | Myxococcales | Anaeromyxobacteraceae | *Anaeromyxobacter* | unidentified | | | | | | | | 0.026 |
| Myxococcia | Myxococcales | Myxococcaceae | *Cystobacter* | unidentified | | 0.943 | 0.827 | 1.504 | | | | |
| Myxococcia | Myxococcales | Myxococcaceae | P3OB-42 | uncultured_*Cystobacterineae* | | | | | | | 0.041 | |
| Myxococcia | Myxococcales | Myxococcaceae | uncultured | uncultured_bacterium | | | | | | | | 0.06 |
| Myxococcia | Myxococcales | Myxococcaceae | unidentified | unidentified | | 0.38 | 0.17 | | 0.354 | | 0.539 | 0.097 |
| Polyangia | Haliangiales | Haliangiaceae | *Haliangium* | uncultured_*Myxococcales* | | | | | | | 0.055 | |
| Polyangia | Haliangiales | Haliangiaceae | *Haliangium* | uncultured_proteobacterium | | | | | 0.055 | | | |
| Polyangia | Haliangiales | Haliangiaceae | *Haliangium* | unidentified | | | | | | 0.134 | 0.286 | 0.166 |

| | | | | | | | | | | | | |
|---|---|---|---|---|---|---|---|---|---|---|---|---|
| Polyangia | mle1-27 | mle1-27 | mle1-27 | unidentified | | | | | | | | 0.077 |
| Polyangia | Polyangiales | BIrii41 | BIrii41 | uncultured_bacterium | | | | | | 0.119 | 0.065 | 0.013 |
| Polyangia | Polyangiales | BIrii41 | BIrii41 | unidentified | | | | | | | | 0.097 |
| Polyangia | Polyangiales | Eel-36e1D6 | Eel-36e1D6 | uncultured_bacterium | | | | | | | | 0.031 |
| Polyangia | Polyangiales | Phaselicystidaceae | *Phaselicystis* | uncultured_bacterium | | | | | | | | 0.026 |
| Polyangia | Polyangiales | Phaselicystidaceae | *Phaselicystis* | uncultured_*Byssovorax* | | | | | | | 0.097 | |
| Polyangia | Polyangiales | Polyangiaceae | *Minicystis* | uncultured_bacterium | | | | | | | | 0.042 |
| Polyangia | Polyangiales | Polyangiaceae | *Pajaroellobacter* | unidentified | | | | | | | | 0.073 |
| Polyangia | Polyangiales | Polyangiaceae | *Polyangiaceae* | uncultured_bacterium | | | | | | | | 0.062 |
| Polyangia | Polyangiales | Sandaracinaceae | uncultured | uncultured_bacterium | | | | | | | 0.041 | |
| Polyangia | Polyangiales | Sandaracinaceae | uncultured | unidentified | | | | | 0.135 | | 0.244 | 0.104 |
| Polyangia | Polyangiales | unidentified | unidentified | unidentified | | | | | | | | 0.018 |
| unidentified | unidentified | unidentified | unidentified | unidentified | | | | | | | 0.028 | |
| **NB1-j** | | | | | | | | | | | | |
| NB1-j | NB1-j | NB1-j | NB1-j | uncultured_*Desulfoglaeba* | | | | | | | 0.074 | 0.073 |
| NB1-j | NB1-j | NB1-j | NB1-j | uncultured_*Myxococcales* | | | | | | | 0.065 | |
| NB1-j | NB1-j | NB1-j | NB1-j | unidentified | | | | | 0.135 | | 0.313 | 0.093 |
| **Nitrospirota** | | | | | | | | | | | | |
| Nitrospiria | Nitrospirales | Nitrospiraceae | *Nitrospira* | *Nitrospira japonica* | | | | | 0.259 | 0.163 | 0.295 | 0.104 |
| Nitrospiria | Nitrospirales | Nitrospiraceae | *Nitrospira* | uncultured_*Nitrospiraceae* | | | | | 0.035 | | | |
| Nitrospiria | Nitrospirales | Nitrospiraceae | *Nitrospira* | uncultured_soil | | | | | | | 0.217 | |
| Nitrospiria | Nitrospirales | Nitrospiraceae | *Nitrospira* | unidentified | | | | | 0.055 | | 0.083 | |

| | | | | | | | | | | | | |
|---|---|---|---|---|---|---|---|---|---|---|---|---|
| **Patescibacteria** | | | | | | | | | | | | |
| Parcubacteria | Candidatus_Kaiserbacteria | Candidatus_Kaiserbacteria | *Candidatus_Kaiserbacteria* | uncultured_bacterium | | | | | | | 0.111 | |
| Parcubacteria | Parcubacteria | Parcubacteria | *Parcubacteria* | uncultured_bacterium | | | | | | | 0.051 | 0.049 |
| Saccharimonadia | Saccharimonadales | LWQ8 | LWQ8 | uncultured_bacterium | | | | | 0.169 | | | |
| Saccharimonadia | Saccharimonadales | LWQ8 | LWQ8 | unidentified | | | | | | 0.163 | 0.129 | |
| Saccharimonadia | Saccharimonadales | S32 | TM7 | unidentified | | | | | | | | 0.073 |
| Saccharimonadia | Saccharimonadales | Saccharimonadaceae | TM7a | uncultured_bacterium | | 0.149 | | | | | | |
| Saccharimonadia | Saccharimonadales | Saccharimonadales | *Saccharimonadales* | unidentified | | | | | 0.095 | | 0.346 | 0.09 |
| Saccharimonadia | Saccharimonadales | unidentified | unidentified | unidentified | | 0.182 | 0.064 | | 0.045 | | 0.24 | 0.15 |
| Saccharimonadia | Saccharimonadales | WWH38 | WWH38 | uncultured_bacterium | | | | | | | 0.032 | |
| **Planctomycetota** | | | | | | | | | | | | |
| OM190 | OM190 | OM190 | OM190 | uncultured_bacterium | | | | | | | | 0.026 |
| Phycisphaerae | Phycisphaerales | Phycisphaeraceae | AKYG587 | uncultured_bacterium | | | | | 0.095 | | 0.032 | 0.022 |
| Phycisphaerae | Phycisphaerales | Phycisphaeraceae | CL500-3 | uncultured_bacterium | | | | | | | 0.046 | |
| Phycisphaerae | Phycisphaerales | Phycisphaeraceae | SM1A02 | unidentified | | | | | | | | 0.011 |
| Phycisphaerae | Tepidisphaerales | WD2101_soil_group | WD2101_soil_group | uncultured_bacterium | | | | | 0.02 | | | |
| Phycisphaerae | Tepidisphaerales | WD2101_soil_group | WD2101_soil_group | uncultured_planctomycete | | | | | | | | 0.018 |
| Phycisphaerae | Tepidisphaerales | WD2101_soil_group | WD2101_soil_group | unidentified | | 0.066 | | | 0.075 | | 0.111 | 0.044 |
| Planctomycetes | Gemmatales | Gemmataceae | uncultured | uncultured_bacterium | | | | | | | | 0.035 |

| | | | | | | | | | | | | |
|---|---|---|---|---|---|---|---|---|---|---|---|---|
| Planctomycetes | Gemmatales | Gemmataceae | uncultured | unidentified | | | | | | | | 0.024 |
| unidentified | unidentified | unidentified | unidentified | unidentified | | | | | | | 0.018 | |
| **Proteobacteria** | | | | | | | | | | | | |
| Alphaproteobacteria | Acetobacterales | Acetobacteraceae | *Acidicaldus* | uncultured_*Acidisphaera* | | | | | | 0.193 | | 0.049 |
| Alphaproteobacteria | Acetobacterales | Acetobacteraceae | *Belnapia* | uncultured_bacterium | 1.151 | 0.248 | | | | | | |
| Alphaproteobacteria | Acetobacterales | Acetobacteraceae | *Roseomonas* | unidentified | | 1.174 | 0.89 | 1.59 | | 0.134 | 0.06 | |
| Alphaproteobacteria | Acetobacterales | Acetobacteraceae | uncultured | uncultured_*Paracraurococcus* | | | | | 0.05 | | | |
| Alphaproteobacteria | Acetobacterales | Acetobacteraceae | unidentified | unidentified | 1.63 | 1.918 | 0.551 | | | | | 0.022 |
| Alphaproteobacteria | Azospirillales | Azospirillaceae | uncultured | uncultured_bacterium | | | | | 0.04 | | 0.028 | |
| Alphaproteobacteria | Azospirillales | Inquilinaceae | *Inquilinus* | *Inquilinus* sp. | | | | | | 0.267 | 0.083 | |
| Alphaproteobacteria | Azospirillales | uncultured | uncultured | unidentified | | | | | 0.11 | | 0.097 | 0.022 |
| Alphaproteobacteria | Caulobacterales | Caulobacteraceae | *Brevundimonas* | drinking_water | | 0.248 | 0.148 | | | | | |
| Alphaproteobacteria | Caulobacterales | Caulobacteraceae | *Brevundimonas* | unidentified | | 2.233 | 0.933 | | | | 0.124 | |
| Alphaproteobacteria | Caulobacterales | Caulobacteraceae | *Caulobacter* | unidentified | | | | | | | 0.433 | |
| Alphaproteobacteria | Caulobacterales | Caulobacteraceae | *Phenylobacterium* | uncultured_soil | | | | | | 0.163 | 0.152 | |
| Alphaproteobacteria | Caulobacterales | Caulobacteraceae | *Phenylobacterium* | unidentified | | | | | 0.165 | | 0.23 | 0.154 |
| Alphaproteobacteria | Caulobacterales | Caulobacteraceae | uncultured | unidentified | | | | | | | | 0.102 |
| Alphaproteobacteria | Caulobacterales | Hyphomonadaceae | *Hirschia* | unidentified | | | | | 0.13 | | 0.014 | |
| Alphaproteobacteria | Caulobacterales | Hyphomonadaceae | SWB02 | uncultured_soil | | | | | | | 0.037 | |

| | | | | | | | | | | | | |
|---|---|---|---|---|---|---|---|---|---|---|---|---|
| Alphaproteobacteria | Caulobacterales | Hyphomonadaceae | SWB02 | unidentified | | | | | | | 0.152 | 0.029 |
| Alphaproteobacteria | Caulobacterales | Parvularculaceae | *Amphiplicatus* | uncultured_bacterium | | | | | | 0.134 | | |
| Alphaproteobacteria | Defluviicoccales | Defluviicoccaceae | *Defluviicoccus* | unidentified | | | | | | | | 0.307 |
| Alphaproteobacteria | Dongiales | Dongiaceae | *Dongia* | metagenome | | | | | 0.174 | 0.327 | | 0.051 |
| Alphaproteobacteria | Dongiales | Dongiaceae | *Dongia* | unidentified | | | | | 1.276 | 1.929 | 4.069 | 0.607 |
| Alphaproteobacteria | Elsterales | uncultured | uncultured | unidentified | | | | | | | 0.101 | |
| Alphaproteobacteria | Holosporales | Holosporaceae | uncultured | uncultured_bacterium | | | | | | | | 0.819 |
| Alphaproteobacteria | Micropepsales | Micropepsaceae | uncultured | unidentified | | | | | 0.03 | | | |
| Alphaproteobacteria | Parvibaculales | Parvibaculaceae | uncultured | uncultured_bacterium | | | | | | | 0.055 | |
| Alphaproteobacteria | Puniceispirillales | Puniceispirillales_Incertae_Sedis | *Constrictibacter* | uncultured_soil | | 0.198 | 0.148 | | | | | |
| Alphaproteobacteria | Reyranellales | Reyranellaceae | *Reyranella* | unidentified | | | | | 0.563 | 2.196 | 1.682 | 2.006 |
| Alphaproteobacteria | Reyranellales | Reyranellaceae | uncultured | uncultured_bacterium | | | | | 0.13 | 0.193 | | 0.086 |
| Alphaproteobacteria | Reyranellales | Reyranellaceae | uncultured | uncultured_*Rhodospirillales* | | | | | | | 0.088 | |
| Alphaproteobacteria | Reyranellales | Reyranellaceae | uncultured | unidentified | | | | | | | 0.212 | |
| Alphaproteobacteria | Rhizobiales | A0839 | A0839 | uncultured_*Phyllobacteriaceae* | | | | | 0.075 | 0.252 | 0.106 | 0.06 |
| Alphaproteobacteria | Rhizobiales | A0839 | A0839 | uncultured_sludge | | | | | | 0.223 | 0.115 | |
| Alphaproteobacteria | Rhizobiales | Beijerinckiaceae | 1174-901-12 | unidentified | | 0.496 | | 0.859 | | | | |
| Alphaproteobacteria | Rhizobiales | Beijerinckiaceae | FFCH5858 | unidentified | | | | | | | | 0.218 |

| | | | | | | | | | | | | |
|---|---|---|---|---|---|---|---|---|---|---|---|---|
| Alphaproteobacteria | Rhizobiales | Beijerinckiaceae | *Methylobacterium-Methylorubrum* | *Methylobacterium aquaticum* | | | | 1.59 | | | | |
| Alphaproteobacteria | Rhizobiales | Beijerinckiaceae | *Methylobacterium-Methylorubrum* | *Methylobacterium brachiatum* | | | | 1.504 | | | | |
| Alphaproteobacteria | Rhizobiales | Beijerinckiaceae | *Methylobacterium-Methylorubrum* | *Methylobacterium hispanicum* | | 1.108 | 6.169 | 2.578 | | | | |
| Alphaproteobacteria | Rhizobiales | Beijerinckiaceae | *Methylobacterium-Methylorubrum* | *Methylobacterium komagatae* | 3.547 | 2.911 | 1.95 | 2.192 | | | | |
| Alphaproteobacteria | Rhizobiales | Beijerinckiaceae | *Methylobacterium-Methylorubrum* | *Methylorubrum podarium* | | 0.711 | | | | | | |
| Alphaproteobacteria | Rhizobiales | Beijerinckiaceae | *Methylobacterium-Methylorubrum* | unidentified | 19.847 | 15.396 | 16.154 | 24.065 | | | | |
| Alphaproteobacteria | Rhizobiales | Beijerinckiaceae | *Microvirga* | unidentified | | 0.281 | 0.657 | | 1.346 | 0.445 | 0.525 | 0.135 |
| Alphaproteobacteria | Rhizobiales | Beijerinckiaceae | unidentified | unidentified | | 0.992 | 0.996 | | 0.145 | | | 0.086 |
| Alphaproteobacteria | Rhizobiales | D05-2 | D05-2 | uncultured_bacterium | | | | | 0.04 | | 0.106 | |
| Alphaproteobacteria | Rhizobiales | Devosiaceae | *Devosia* | unidentified | | 1.637 | 0.254 | | | | 0.161 | |
| Alphaproteobacteria | Rhizobiales | Devosiaceae | uncultured | unidentified | | | | | | | 0.253 | |
| Alphaproteobacteria | Rhizobiales | Devosiaceae | unidentified | unidentified | | | 0.424 | | | | | |
| Alphaproteobacteria | Rhizobiales | Hyphomicrobiaceae | *Hyphomicrobium* | uncultured_*Hyphomicrobium* | | | | | | 0.416 | | 0.011 |
| Alphaproteobacteria | Rhizobiales | Hyphomicrobiaceae | *Hyphomicrobium* | unidentified | | | | | 0.224 | 0.282 | 0.41 | |
| Alphaproteobacteria | Rhizobiales | Hyphomicrobiaceae | *Pedomicrobium* | uncultured_bacterium | | | | | 0.135 | 0.237 | 0.161 | |
| Alphaproteobacteria | Rhizobiales | Hyphomicrobiaceae | *Pedomicrobium* | uncultured_*Hyphomicrobiaceae* | | | | | 0.354 | | 0.553 | 0.252 |
| Alphaproteobacteria | Rhizobiales | Hyphomicrobiaceae | *Pedomicrobium* | unidentified | | | | | 0.334 | 3.413 | 0.991 | 0.11 |
| Alphaproteobacteria | Rhizobiales | Hyphomicrobiaceae | unidentified | unidentified | | | | | | | 0.028 | |

| | | | | | | | | | | | | |
|---|---|---|---|---|---|---|---|---|---|---|---|---|
| Alphaproteobacteria | Rhizobiales | KF-JG30-B3 | KF-JG30-B3 | uncultured_bacterium | | | | | 0.264 | | 0.24 | |
| Alphaproteobacteria | Rhizobiales | KF-JG30-B3 | KF-JG30-B3 | unidentified | | | | | | | 0.327 | 0.17 |
| Alphaproteobacteria | Rhizobiales | Labraceae | *Labrys* | unidentified | | | | | | | 0.028 | |
| Alphaproteobacteria | Rhizobiales | Methyloligellaceae | *Methyloligellaceae* | uncultured_bacterium | | | | | 0.578 | 0.49 | 1.055 | |
| Alphaproteobacteria | Rhizobiales | Methyloligellaceae | unidentified | unidentified | | | | | | | | 0.073 |
| Alphaproteobacteria | Rhizobiales | Methylopilaceae | unidentified | unidentified | | 0.347 | | | | | | |
| Alphaproteobacteria | Rhizobiales | Rhizobiaceae | *Allorhizobium-Neorhizobium-Pararhizobium-Rhizobium* | unidentified | 5.944 | 4.581 | 1.42 | 4.168 | 0.354 | 1.647 | 1.276 | 0.23 |
| Alphaproteobacteria | Rhizobiales | Rhizobiaceae | *Aureimonas* | *Aurantimonas* sp. | | 1.306 | 0.996 | | | | | |
| Alphaproteobacteria | Rhizobiales | Rhizobiaceae | *Aureimonas* | *Aureimonas jatrophae* | 11.218 | 4.994 | 5.109 | 21.917 | | | | |
| Alphaproteobacteria | Rhizobiales | Rhizobiaceae | *Aureimonas* | *Aureimonas phyllosphaerae* | | 0.827 | 0.827 | 0.473 | | | | |
| Alphaproteobacteria | Rhizobiales | Rhizobiaceae | *Aureimonas* | *Aureimonas rubiginis* | | 0.413 | | 2.192 | | | | |
| Alphaproteobacteria | Rhizobiales | Rhizobiaceae | *Aureimonas* | uncultured_bacterium | | | 0.127 | | | | | |
| Alphaproteobacteria | Rhizobiales | Rhizobiaceae | *Aureimonas* | unidentified | 9.684 | 7.557 | 6.084 | 7.263 | | | | |
| Alphaproteobacteria | Rhizobiales | Rhizobiaceae | *Ensifer* | unidentified | | | | | 0.09 | 0.534 | 0.272 | |
| Alphaproteobacteria | Rhizobiales | Rhizobiaceae | *Mesorhizobium* | unidentified | 1.63 | 1.439 | 1.399 | | 0.678 | 1.558 | 0.461 | 0.245 |
| Alphaproteobacteria | Rhizobiales | Rhizobiaceae | *Tianweitania* | uncultured_bacterium | | 0.695 | | | | | | |
| Alphaproteobacteria | Rhizobiales | Rhizobiaceae | unidentified | unidentified | 4.219 | 7.64 | 5.109 | 1.633 | 0.04 | 0.579 | 0.825 | |

| | | | | | | | | | | | | |
|---|---|---|---|---|---|---|---|---|---|---|---|---|
| Alphaproteobacteria | Rhizobiales | Rhizobiales_Incertae_Sedis | *Bauldia* | uncultured_bacterium | | | | | | | 0.078 | |
| Alphaproteobacteria | Rhizobiales | Rhizobiales_Incertae_Sedis | *Bauldia* | uncultured_*Phyllobacteriaceae* | | | | | 0.03 | | | |
| Alphaproteobacteria | Rhizobiales | Rhizobiales_Incertae_Sedis | *Bauldia* | unidentified | | | | | 0.02 | | 0.051 | |
| Alphaproteobacteria | Rhizobiales | Rhizobiales_Incertae_Sedis | *Nordella* | uncultured_*Alphaproteobacteria* | | | | | 0.284 | | | 0.066 |
| Alphaproteobacteria | Rhizobiales | Rhizobiales_Incertae_Sedis | *Nordella* | uncultured_*Phyllobacteriaceae* | | | | | 0.145 | 0.401 | 0.078 | 0.044 |
| Alphaproteobacteria | Rhizobiales | Rhizobiales_Incertae_Sedis | *Nordella* | unidentified | | | | | 0.09 | 0.95 | 0.728 | 0.121 |
| Alphaproteobacteria | Rhizobiales | Rhizobiales_Incertae_Sedis | unidentified | unidentified | | | | | | 0.252 | | |
| Alphaproteobacteria | Rhizobiales | Rhodomicrobiaceae | *Rhodomicrobium* | uncultured_*Alphaproteobacteria* | | | | | 0.14 | | | |
| Alphaproteobacteria | Rhizobiales | Rhodomicrobiaceae | *Rhodomicrobium* | uncultured_bacterium | | | | | 0.065 | 0.386 | 0.295 | |
| Alphaproteobacteria | Rhizobiales | Rhodomicrobiaceae | *Rhodomicrobium* | unidentified | | | | | | 0.282 | 0.147 | |
| Alphaproteobacteria | Rhizobiales | Stappiaceae | *Agaricicola* | *Agaricicola taiwanensis* | | 1.753 | 1.336 | | | | | |
| Alphaproteobacteria | Rhizobiales | uncultured | uncultured | uncultured_*Alphaproteobacteria* | | | | | | | 0.143 | |
| Alphaproteobacteria | Rhizobiales | uncultured | uncultured | unidentified | | | | | 0.374 | 0.876 | 0.571 | 1.006 |
| Alphaproteobacteria | Rhizobiales | unidentified | unidentified | unidentified | 4.89 | 2.828 | 6.826 | 5.93 | 0.628 | 0.638 | 0.622 | 0.055 |
| Alphaproteobacteria | Rhizobiales | Xanthobacteraceae | *Bradyrhizobium* | unidentified | | | | | 1.62 | 5.239 | 1.737 | 0.781 |
| Alphaproteobacteria | Rhizobiales | Xanthobacteraceae | *Pseudolabrys* | uncultured_*Bradyrhizobiaceae* | | | | | 0.553 | 0.371 | 0.129 | |
| Alphaproteobacteria | Rhizobiales | Xanthobacteraceae | *Pseudolabrys* | unidentified | | | | | | | | 0.04 |
| Alphaproteobacteria | Rhizobiales | Xanthobacteraceae | *Rhodoplanes* | uncultured_bacterium | | | | | | | | 0.099 |

| | | | | | | | | | | | | |
|---|---|---|---|---|---|---|---|---|---|---|---|---|
| Alphaproteobacteria | Rhizobiales | Xanthobacteraceae | *Rhodoplanes* | uncultured_*Rhodoplanes* | | | | | 0.204 | | | 0.636 |
| Alphaproteobacteria | Rhizobiales | Xanthobacteraceae | *Rhodoplanes* | unidentified | | | | | 0.229 | 0.579 | 0.235 | 0.64 |
| Alphaproteobacteria | Rhizobiales | Xanthobacteraceae | uncultured | uncultured_*Blastochloris* | | | | | 0.254 | 0.327 | | 0.053 |
| Alphaproteobacteria | Rhizobiales | Xanthobacteraceae | uncultured | uncultured_*Bradyrhizobiaceae* | | | | | | | 0.115 | |
| Alphaproteobacteria | Rhizobiales | Xanthobacteraceae | uncultured | uncultured_*Pseudolabrys* | | | | | | | | 0.424 |
| Alphaproteobacteria | Rhizobiales | Xanthobacteraceae | uncultured | unidentified | | | | | 3.26 | 3.057 | 3.014 | 5.636 |
| Alphaproteobacteria | Rhizobiales | Xanthobacteraceae | unidentified | unidentified | | 0.298 | | | 1.177 | 2.968 | 2.152 | 2.119 |
| Alphaproteobacteria | Rhizobiales | Xanthobacteraceae | *Xanthobacteraceae* | unidentified | | | | | | | 0.124 | |
| Alphaproteobacteria | Rhodobacterales | Rhodobacteraceae | *Falsirhodobacter* | unidentified | | | | | | | 0.046 | |
| Alphaproteobacteria | Rhodobacterales | Rhodobacteraceae | *Paracoccus* | unidentified | | | | | 0.174 | | 0.055 | |
| Alphaproteobacteria | Rhodobacterales | Rhodobacteraceae | *Rubellimicrobium* | *Rubellimicrobium mesophilum* | | | | | 0.155 | | | |
| Alphaproteobacteria | Rhodobacterales | Rhodobacteraceae | *Rubellimicrobium* | uncultured_bacterium | | 0.116 | | | | | | |
| Alphaproteobacteria | Rhodobacterales | Rhodobacteraceae | unidentified | unidentified | | | | | | | | 0.031 |
| Alphaproteobacteria | Rhodospirillales | Rhodospirillaceae | uncultured | metagenome | | | | | | | | 0.06 |
| Alphaproteobacteria | Rhodospirillales | Rhodospirillaceae | uncultured | unidentified | | | | | | | 0.06 | |
| Alphaproteobacteria | Sphingomonadales | Sphingomonadaceae | *Altererythrobacter* | unidentified | | | | | 0.1 | | 0.161 | 0.093 |
| Alphaproteobacteria | Sphingomonadales | Sphingomonadaceae | Ellin6055 | unidentified | | | | | | | | 0.15 |
| Alphaproteobacteria | Sphingomonadales | Sphingomonadaceae | *Novosphingobium* | *Novosphingobium gossypii* | | | | | | | 0.065 | |

| | | | | | | | | | | | | |
|---|---|---|---|---|---|---|---|---|---|---|---|---|
| Alphaproteobacteria | Sphingomonadales | Sphingomonadaceae | *Novosphingobium* | unidentified | | | | | | 0.148 | 0.442 | |
| Alphaproteobacteria | Sphingomonadales | Sphingomonadaceae | *Porphyrobacter* | unidentified | | | | | | | | 0.097 |
| Alphaproteobacteria | Sphingomonadales | Sphingomonadaceae | *Qipengyuania* | unidentified | | | | | | | 0.046 | |
| Alphaproteobacteria | Sphingomonadales | Sphingomonadaceae | *Sphingobium* | unidentified | | | | | | | 0.415 | 0.23 |
| Alphaproteobacteria | Sphingomonadales | Sphingomonadaceae | *Sphingomonas* | *Caulobacter* sp. | | | | | | 0.505 | | |
| Alphaproteobacteria | Sphingomonadales | Sphingomonadaceae | *Sphingomonas* | *Sphingomonas gimensis* | | 0.843 | 0.7 | 0.602 | | | | |
| Alphaproteobacteria | Sphingomonadales | Sphingomonadaceae | *Sphingomonas* | *Sphingomonas guangdongensis* | | 0.579 | | 0.559 | | | | |
| Alphaproteobacteria | Sphingomonadales | Sphingomonadaceae | *Sphingomonas* | *Sphingomonas phyllosphaerae* | | 0.182 | | | | | | |
| Alphaproteobacteria | Sphingomonadales | Sphingomonadaceae | *Sphingomonas* | *Sphingomonas rhizophila* | | | | | | | 0.111 | |
| Alphaproteobacteria | Sphingomonadales | Sphingomonadaceae | *Sphingomonas* | unidentified | | 3.456 | 2.141 | 5.243 | 0.573 | | 0.396 | 0.161 |
| Alphaproteobacteria | Sphingomonadales | Sphingomonadaceae | unidentified | unidentified | 6.328 | 5.093 | 9.307 | 2.235 | 0.01 | | 0.35 | 0.26 |
| Alphaproteobacteria | Tistrellales | Geminicoccaceae | uncultured | uncultured_*Acetobacteraceae* | | | | | | | | 0.022 |
| Alphaproteobacteria | Tistrellales | Geminicoccaceae | uncultured | uncultured_bacterium | | | | | | | 0.083 | 0.075 |
| Alphaproteobacteria | Tistrellales | Geminicoccaceae | uncultured | unidentified | | | | | | | 0.23 | 0.046 |
| Alphaproteobacteria | uncultured | uncultured | uncultured | *Rhodospirillaceae bacterium* | | | | | | | 0.078 | 0.102 |
| Alphaproteobacteria | uncultured | uncultured | uncultured | uncultured_*Acetobacteraceae* | | | | | | 0.237 | | 0.117 |
| Alphaproteobacteria | uncultured | uncultured | uncultured | uncultured_bacterium | | | | | | | 0.009 | 0.033 |
| Alphaproteobacteria | uncultured | uncultured | uncultured | uncultured_*Rhodospirillaceae* | | | | | | | | 0.024 |

| | | | | | | | | | | | | |
|---|---|---|---|---|---|---|---|---|---|---|---|---|
| Alphaproteobacteria | uncultured | uncultured | uncultured | uncultured_soil | | | | | 0.189 | 0.297 | 0.484 | |
| Alphaproteobacteria | uncultured | uncultured | uncultured | unidentified | | | | | 0.09 | | 0.783 | 0.17 |
| Alphaproteobacteria | unidentified | unidentified | unidentified | unidentified | | | 0.148 | | 0.867 | 0.816 | 0.493 | 0.313 |
| Gammaproteobacteria | Burkholderiales | A21b | A21b | unidentified | | | | | 0.045 | | | 0.033 |
| Gammaproteobacteria | Burkholderiales | B1-7BS | B1-7BS | uncultured_bacterium | | | | | 0.05 | | 0.276 | 0.044 |
| Gammaproteobacteria | Burkholderiales | Burkholderiaceae | *Burkholderia-Caballeronia-Paraburkholderia* | uncultured_*Burkholderiales* | | | | | | | 0.078 | |
| Gammaproteobacteria | Burkholderiales | Burkholderiaceae | *Burkholderia-Caballeronia-Paraburkholderia* | unidentified | | 0.265 | | | | | | |
| Gammaproteobacteria | Burkholderiales | Burkholderiaceae | *Cupriavidus* | unidentified | | | | | 0.125 | 0.341 | 0.184 | |
| Gammaproteobacteria | Burkholderiales | Burkholderiaceae | *Ralstonia* | unidentified | | | | | | | | 0.238 |
| Gammaproteobacteria | Burkholderiales | Comamonadaceae | *Azohydromonas* | uncultured_bacterium | | | | | | | | 0.02 |
| Gammaproteobacteria | Burkholderiales | Comamonadaceae | *Ramlibacter* | unidentified | | | | | 0.688 | | 0.276 | |
| Gammaproteobacteria | Burkholderiales | Comamonadaceae | *Rivibacter* | uncultured_bacterium | | | | | 0.08 | | 0.12 | 0.291 |
| Gammaproteobacteria | Burkholderiales | Comamonadaceae | *Roseateles* | unidentified | | | | | | | 0.101 | |
| Gammaproteobacteria | Burkholderiales | Comamonadaceae | unidentified | unidentified | | 0.281 | | | 0.234 | | 0.479 | 0.19 |
| Gammaproteobacteria | Burkholderiales | Comamonadaceae | *Variovorax* | unidentified | | | | | | 0.163 | 0.124 | |
| Gammaproteobacteria | Burkholderiales | Methylophilaceae | MM2 | uncultured_bacterium | | | | | | | 0.041 | |
| Gammaproteobacteria | Burkholderiales | Nitrosomonadaceae | Ellin6067 | uncultured_*Oxalobacteraceae* | | | | | | | | 0.11 |

| | | | | | | | | | | | | |
|---|---|---|---|---|---|---|---|---|---|---|---|---|
| Gammaproteobacteria | Burkholderiales | Nitrosomonadaceae | Ellin6067 | unidentified | | | | | 0.374 | 0.223 | 0.244 | 0.437 |
| Gammaproteobacteria | Burkholderiales | Nitrosomonadaceae | MND1 | uncultured_*Nitrosomonadales* | | | | | | | | 0.062 |
| Gammaproteobacteria | Burkholderiales | Nitrosomonadaceae | MND1 | unidentified | | | | | 0.513 | 0.608 | 0.917 | 0.329 |
| Gammaproteobacteria | Burkholderiales | Oxalobacteraceae | *Duganella* | unidentified | | | | | | | 0.06 | |
| Gammaproteobacteria | Burkholderiales | Oxalobacteraceae | *Massilia* | *Massilia albidiflava* | | | | | 0.199 | | 0.166 | |
| Gammaproteobacteria | Burkholderiales | Oxalobacteraceae | *Massilia* | *Massilia oculi* | | | | | | | 0.065 | |
| Gammaproteobacteria | Burkholderiales | Oxalobacteraceae | *Massilia* | unidentified | | | | | 0.085 | | 0.364 | 0.238 |
| Gammaproteobacteria | Burkholderiales | Oxalobacteraceae | unidentified | unidentified | | | | | | | 0.111 | |
| Gammaproteobacteria | Burkholderiales | Rhodocyclaceae | unidentified | unidentified | | | | | | | 0.078 | 0.024 |
| Gammaproteobacteria | Burkholderiales | SC-I-84 | SC-I-84 | uncultured_bacterium | | | | | | | 0.083 | |
| Gammaproteobacteria | Burkholderiales | SC-I-84 | SC-I-84 | unidentified | | | | | | | 0.152 | 0.172 |
| Gammaproteobacteria | Burkholderiales | Sutterellaceae | uncultured | uncultured_bacterium | | | | | 0.209 | | 0.359 | 0.079 |
| Gammaproteobacteria | Burkholderiales | TRA3-20 | TRA3-20 | bacterium_enrichment | | | | | 0.075 | 0.282 | 0.198 | 0.121 |
| Gammaproteobacteria | Burkholderiales | TRA3-20 | TRA3-20 | uncultured_bacterium | | | | | | | 0.101 | |
| Gammaproteobacteria | Burkholderiales | TRA3-20 | TRA3-20 | uncultured_*Green* | | | | | | | 0.129 | |
| Gammaproteobacteria | Burkholderiales | TRA3-20 | TRA3-20 | uncultured_*Nitrosomonadales* | | | | | 0.409 | | | 0.097 |
| Gammaproteobacteria | Burkholderiales | TRA3-20 | TRA3-20 | unidentified | | | | | 0.189 | | 0.3 | 0.329 |
| Gammaproteobacteria | Burkholderiales | unidentified | unidentified | unidentified | | | | | | | 0.023 | 0.104 |

| | | | | | | | | | | | | |
|---|---|---|---|---|---|---|---|---|---|---|---|---|
| Gammaproteobacteria | CCD24 | CCD24 | CCD24 | uncultured_gamma | | | | | | | 0.212 | |
| Gammaproteobacteria | CCD24 | CCD24 | CCD24 | unidentified | | | | | 0.179 | 0.312 | 0.548 | |
| Gammaproteobacteria | Diplorickettsiales | Diplorickettsiaceae | *Aquicella* | uncultured_*Aquicella* | | | | | | | | 0.029 |
| Gammaproteobacteria | Diplorickettsiales | Diplorickettsiaceae | *Aquicella* | uncultured_bacterium | | | | | | | | 1.953 |
| Gammaproteobacteria | Diplorickettsiales | Diplorickettsiaceae | *Aquicella* | unidentified | | | | | | | | 0.839 |
| Gammaproteobacteria | Diplorickettsiales | Diplorickettsiaceae | uncultured | agricultural_soil | | | | | | | | 0.024 |
| Gammaproteobacteria | Diplorickettsiales | Diplorickettsiaceae | uncultured | unidentified | | | | | | | | 0.373 |
| Gammaproteobacteria | Diplorickettsiales | Diplorickettsiaceae | unidentified | unidentified | | | | | | | | 0.071 |
| Gammaproteobacteria | Enterobacterales | Erwiniaceae | *Pantoea* | unidentified | | 0.182 | | | 0.055 | | | |
| Gammaproteobacteria | EV818SWSAP88 | EV818SWSAP88 | EV818SWSAP88 | uncultured_bacterium | | | | | | | | 0.088 |
| Gammaproteobacteria | Gammaproteobacteria_Incertae_Sedis | Unknown_Family | *Acidibacter* | metagenome | | | | | 0.09 | | | |
| Gammaproteobacteria | Gammaproteobacteria_Incertae_Sedis | Unknown_Family | *Acidibacter* | uncultured_bacterium | | | | | | | 0.078 | |
| Gammaproteobacteria | Gammaproteobacteria_Incertae_Sedis | Unknown_Family | *Acidibacter* | unidentified | | | | | 0.862 | 1.217 | 1.668 | 0.256 |
| Gammaproteobacteria | Gammaproteobacteria_Incertae_Sedis | Unknown_Family | unidentified | unidentified | | | | | | | | 0.018 |
| Gammaproteobacteria | JG36-GS-52 | JG36-GS-52 | JG36-GS-52 | uncultured_bacterium | | | | | | | | 0.062 |
| Gammaproteobacteria | Legionellales | Legionellaceae | *Legionella* | *Legionella adelaidensis* | | | | | | | | 0.146 |

| | | | | | | | | | | | | |
|---|---|---|---|---|---|---|---|---|---|---|---|---|
| Gammaproteobacteria | Legionellales | Legionellaceae | *Legionella* | uncultured_bacterium | | | | | | | | 0.038 |
| Gammaproteobacteria | Legionellales | Legionellaceae | *Legionella* | unidentified | | | | | | | | 3.699 |
| Gammaproteobacteria | PLTA13 | PLTA13 | PLTA13 | uncultured_bacterium | | | | | | | | 0.038 |
| Gammaproteobacteria | PLTA13 | PLTA13 | PLTA13 | uncultured_gamma | | | | | 0.085 | 0.297 | 0.276 | 0.062 |
| Gammaproteobacteria | PLTA13 | PLTA13 | PLTA13 | unidentified | | | | | | | | 0.057 |
| Gammaproteobacteria | Pseudomonadales | Pseudomonadaceae | *Pseudomonas* | *Pseudomonas psychrotolerans* | 0.767 | | | | | | | |
| Gammaproteobacteria | Pseudomonadales | Pseudomonadaceae | *Pseudomonas* | unidentified | | | | | 0.055 | | | |
| Gammaproteobacteria | Salinisphaerales | Solimonadaceae | *Polycyclovorans* | uncultured_bacterium | | | | | | | | 0.02 |
| Gammaproteobacteria | Steroidobacterales | Steroidobacteraceae | *Steroidobacter* | uncultured_*Xanthomonadaceae* | | | | | | | | 0.035 |
| Gammaproteobacteria | Steroidobacterales | Steroidobacteraceae | *Steroidobacter* | unidentified | | | | | 1.122 | 0.905 | 1.373 | 0.23 |
| Gammaproteobacteria | Steroidobacterales | Steroidobacteraceae | uncultured | unidentified | | | | | 0.095 | | | 0.04 |
| Gammaproteobacteria | Steroidobacterales | Steroidobacteraceae | unidentified | unidentified | | | | | 0.08 | | 0.263 | |
| Gammaproteobacteria | unidentified | unidentified | unidentified | unidentified | | | | | 0.09 | | 0.083 | 0.179 |
| Gammaproteobacteria | Xanthomonadales | Rhodanobacteraceae | *Luteibacter* | *Luteibacter yeojuensis* | | 0.149 | | | | | | |
| Gammaproteobacteria | Xanthomonadales | Xanthomonadaceae | *Arenimonas* | unidentified | | | | | | | | 0.057 |
| Gammaproteobacteria | Xanthomonadales | Xanthomonadaceae | *Lysobacter* | *Lysobacter bugurensis* | | | | | | | | 0.035 |
| Gammaproteobacteria | Xanthomonadales | Xanthomonadaceae | *Lysobacter* | *Lysobacter dokdonensis* | | | | | 0.219 | | | |
| Gammaproteobacteria | Xanthomonadales | Xanthomonadaceae | *Lysobacter* | unidentified | | | | | 0.05 | 0.178 | 0.475 | |

| | | | | | | | | | | | | |
|---|---|---|---|---|---|---|---|---|---|---|---|---|
| Gammaproteobacteria | Xanthomonadales | Xanthomonadaceae | *Stenotrophomonas* | unidentified | 3.356 | 0.099 | | | | | | 0.064 |
| Gammaproteobacteria | Xanthomonadales | Xanthomonadaceae | unidentified | unidentified | | 0.198 | | | | | 0.217 | |
| Gammaproteobacteria | Xanthomonadales | Xanthomonadaceae | *Xanthomonas* | unidentified | 1.534 | | 0.276 | | | | | |
| unidentified | unidentified | unidentified | unidentified | unidentified | | | 0.339 | | | | 0.304 | 0.062 |
| **SAR324_clade_(Marine_group_B)** | | | | | | | | | | | | |
| SAR324_clade (Marine_group_B) | SAR324_clade (Marine_group_B) | SAR324_clade (Marine_group_B) | SAR324_clade (Marine_group_B) | uncultured_bacterium | | | | | | | | 0.018 |
| **Verrucomicrobiota** | | | | | | | | | | | | |
| Omnitrophia | Omnitrophales | Omnitrophaceae | *Candidatus_Omnitrophus* | unidentified | | | | | | | | 0.02 |
| **WPS-2** | | | | | | | | | | | | |
| WPS-2 | WPS-2 | WPS-2 | WPS-2 | metagenome | | | | | | | | 0.108 |
