# Supplementary material for: Hidden Tenants: Microbiota of the Rhizosphere and Phyllosphere of Cordia dodecandra Trees in Mayan Forests and Homegardens
Source: Plants (Basel). 2022 Nov 15;11(22):3098. doi: 10.3390/plants11223098 (PMC9699097; doi:10.3390/plants11223098)
Supplement: Supplementary file 1 [file plants-11-03098-s001.zip › plants-1975968-Supplementary material Table S2.pdf]

Hidden Tenants: Microbiota of the Rhizosphere and Phyllosphere of *Cordia dodecandra* Trees in Mayan Forests and Homegardens

Carla G. May-Mutul, Miguel A. López-Garrido, Aileen O'Connor-Sánchez, Yuri J. Peña-Ramírez, Natalia Y. Labrín-Sotomayor, Héctor Estrada-Medina and Miriam M. Ferrer

Supplementary material Table S2. Taxonomic classification assigned to 1096 fungi OTUs from phyllosphere (phy) and rhizosphere (rhi) *Cordia dodecandra* microbiota in Yucatán´s forest (for) and homegarden (hom) populations of the Northeast (NE) and Southwest (SW) region. The last 8 columns present the percent (relative frequency) per sample coded by region, population, and compartment.

Class	Order	Family	Genus	Species	SW Hom Phy	NE For Phy	NE Hom Phy	SW For Phy	NE Hom Rhi	NE For Rhi	SW Hom Rhi	SW For Rhi
**Ascomycota**												
Ascomycota *Inc. sed.*	Ascomycota *Inc. sed.*	Ascomycota *Inc. sed.*	*Gyrothrix*	*Gyrothrix circinata*						0.108		
Ascomycota *Inc. sed.*	Ascomycota *Inc. sed.*	Ascomycota *Inc. sed.*	*Virgaria*	*Virgaria nigra*					0.019			
Dothideomycetes	Botryosphaeriales	Botryosphaeriaceae	*Botryosphaeria*	unidentified						0.594		
Dothideomycetes	Botryosphaeriales	Botryosphaeriaceae	*Botryosphaeria*	unidentified							0.306	
Dothideomycetes	Botryosphaeriales	Botryosphaeriaceae	*Botryosphaeria*	unidentified					0.073			
Dothideomycetes	Botryosphaeriales	Botryosphaeriaceae	*Botryosphaeria*	unidentified					0.073			
Dothideomycetes	Botryosphaeriales	Botryosphaeriaceae	*Botryosphaeria*	unidentified					0.035			
Dothideomycetes	Botryosphaeriales	Botryosphaeriaceae	*Botryosphaeria*	unidentified					0.027			
Dothideomycetes	Botryosphaeriales	Botryosphaeriaceae	*Botryosphaeria*	unidentified						0.047		
Dothideomycetes	Capnodiales	Cladosporiaceae	*Cladosporium*	*Cladosporium adianticola*					0.085	0.04	0.602	
Dothideomycetes	Capnodiales	Cladosporiaceae	*Cladosporium*	*Cladosporium cladosporioides*		0.037						
Dothideomycetes	Capnodiales	Cladosporiaceae	*Cladosporium*	unidentified					0.031	0.081	0.043	0.278

Dothideomycetes	Capnodiales	Cladosporiaceae	*Cladosporium*	unidentified								0.126
Dothideomycetes	Capnodiales	Mycosphaerellaceae	*Annellosympodiella*	*Annellosympodiella juniperi*	0.024		0.024	0.695				
Dothideomycetes	Capnodiales	Mycosphaerellaceae	*Mycosphaerella*	unidentified				0.268				
Dothideomycetes	Capnodiales	Mycosphaerellaceae	*Mycosphaerella*	unidentified				0.129				
Dothideomycetes	Capnodiales	Mycosphaerellaceae	unidentified	unidentified				1.201				
Dothideomycetes	Capnodiales	Mycosphaerellaceae	unidentified	unidentified		0.21						
Dothideomycetes	Capnodiales	Mycosphaerellaceae	*Zasmidium*	*Zasmidium rothmanniae*				0.347				
Dothideomycetes	Capnodiales	unidentified	unidentified	unidentified	0.033	0.03	0.033	0.347				
Dothideomycetes	Capnodiales	unidentified	unidentified	unidentified	0.033		0.033					
Dothideomycetes	Capnodiales	unidentified	unidentified	unidentified		0.09						
Dothideomycetes	Capnodiales	unidentified	unidentified	unidentified				0.109				
Dothideomycetes	Capnodiales	unidentified	unidentified	unidentified		0.075						
Dothideomycetes	Capnodiales	unidentified	unidentified	unidentified				0.099				
Dothideomycetes	Capnodiales	unidentified	unidentified	unidentified		0.067						
Dothideomycetes	Capnodiales	unidentified	unidentified	unidentified				0.079				
Dothideomycetes	Capnodiales	unidentified	unidentified	unidentified				0.079				
Dothideomycetes	Capnodiales	unidentified	unidentified	unidentified	0.01		0.01					
Dothideomycetes	Capnodiales	unidentified	unidentified	unidentified				0.03				
Dothideomycetes	Capnodiales	unidentified	unidentified	unidentified								0.505
Dothideomycetes	Dothideales	Dothideales *Inc. sed.*	*Hortaea*	*Hortaea werneckii*								0.177
Dothideomycetes	Dothideomycetes *Inc. sed.*	Dothideomycetes *Inc. sed.*	*Leptodiscella*	*Leptodiscella africana*					0.062			
Dothideomycetes	Dothideomycetes *Inc. sed.*	Dothideomycetes *Inc. sed.*	*Paramicrothyrium*	*unidentified*	0.057		0.057					
Dothideomycetes	Myriangiales	Elsinoaceae	unidentified	unidentified				0.318				

Dothideomycetes	Myriangiales	Elsinoaceae	unidentified	unidentified	0.038		0.038					
Dothideomycetes	Myriangiales	Elsinoaceae	unidentified	unidentified				0.159				
Dothideomycetes	Myriangiales	Elsinoaceae	unidentified	unidentified		0.09						
Dothideomycetes	Myriangiales	Elsinoaceae	unidentified	unidentified				0.109				
Dothideomycetes	Pleosporales	Coniothyriaceae	unidentified	unidentified		0.157						
Dothideomycetes	Pleosporales	Coniothyriaceae	unidentified	unidentified				0.089				
Dothideomycetes	Pleosporales	Corynesporascaceae	*Corynespora*	unidentified	0.09		0.09					
Dothideomycetes	Pleosporales	Dictyosporiaceae	*Gregarithecium*	*Gregarithecium curvisporum*				0.02				
Dothideomycetes	Pleosporales	Dictyosporiaceae	*Pseudocoleophoma*	*Pseudocoleophoma calamagrostidis*	0.024		0.024		0.035			
Dothideomycetes	Pleosporales	Didymellaceae	*Allophoma*	*Allophoma tropica*	0.029	0.12	0.029	0.03				
Dothideomycetes	Pleosporales	Didymosphaeriaceae	*Dendrothyrium*	unidentified				0.06				
Dothideomycetes	Pleosporales	Didymosphaeriaceae	*Letendraea*	unidentified	0.09		0.09		0.015			
Dothideomycetes	Pleosporales	Didymosphaeriaceae	*Letendraea*	unidentified				0.069				
Dothideomycetes	Pleosporales	Didymosphaeriaceae	*Paraconiothyrium*	unidentified		0.03						
Dothideomycetes	Pleosporales	Didymosphaeriaceae	*Paraphaeosphaeria*	unidentified		0.082						
Dothideomycetes	Pleosporales	Didymosphaeriaceae	*Paraphaeosphaeria*	unidentified	0.024		0.024					
Dothideomycetes	Pleosporales	Didymosphaeriaceae	*Pseudopithomyces*	*Pseudopithomyces chartarum*	0.181	0.075	0.181	0.278			0.435	
Dothideomycetes	Pleosporales	Didymosphaeriaceae	unidentified	unidentified	0.067		0.067	0.228				
Dothideomycetes	Pleosporales	Didymosphaeriaceae	unidentified	unidentified	0.024		0.024					
Dothideomycetes	Pleosporales	Massarinaceae	*Helminthosporium*	unidentified		0.022						
Dothideomycetes	Pleosporales	Periconiaceae	*Periconia*	*Periconia homothallica*				0.03				

Dothideomycetes	Pleosporales	Phaeosphaeriaceae	*Neosetophoma*	*Neosetophoma samararum*		0.045						
Dothideomycetes	Pleosporales	Pleosporaceae	*Alternaria*	*Alternaria macrospora*					0.015		0.027	
Dothideomycetes	Pleosporales	Pleosporaceae	*Alternaria*	unidentified	0.019	0.045	0.019	0.089		0.081		
Dothideomycetes	Pleosporales	Pleosporaceae	*Alternaria*	unidentified					0.05			
Dothideomycetes	Pleosporales	Pleosporaceae	*Curvularia*	*Curvularia clavata*	0.038		0.038		0.077			
Dothideomycetes	Pleosporales	Pleosporaceae	*Curvularia*	*Curvularia lunata*	0.147		0.147		0.135		0.124	
Dothideomycetes	Pleosporales	Pleosporaceae	*Curvularia*	unidentified	0.128	0.12	0.128	0.089	0.012			
Dothideomycetes	Pleosporales	Pleosporaceae	*Exserohilum*	unidentified				0.06				
Dothideomycetes	Pleosporales	Pleosporales *Inc. sed.*	*Matsushimamyces*	*Matsushimamyces venustum*						0.067	0.07	
Dothideomycetes	Pleosporales	Pleosporales *Inc. sed.*	*Pseudorobillarda*	*Pseudorobillarda sojae*	0.014		0.014					
Dothideomycetes	Pleosporales	Pleosporales *Inc. sed.*	unidentified	unidentified							0.086	
Dothideomycetes	Pleosporales	Sporormiaceae	*Preussia*	unidentified					0.05			
Dothideomycetes	Pleosporales	Tetraplosphaeriaceae	*Tetraplosphaeria*	*Tetraplosphaeria yakushimensis*							0.059	
Dothideomycetes	Pleosporales	Thyridariaceae	*Roussoella*	unidentified	0.024		0.024		0.058			
Dothideomycetes	Pleosporales	unidentified	unidentified	unidentified		5.115		10.667				
Dothideomycetes	Pleosporales	unidentified	unidentified	unidentified		12.433			0.22			
Dothideomycetes	Pleosporales	unidentified	unidentified	unidentified	3.812		3.812					
Dothideomycetes	Pleosporales	unidentified	unidentified	unidentified	2.457		2.457	2.917		0.216	0.102	
Dothideomycetes	Pleosporales	unidentified	unidentified	unidentified	0.428	0.846	0.428					
Dothideomycetes	Pleosporales	unidentified	unidentified	unidentified	0.114		0.114	0.655				
Dothideomycetes	Pleosporales	unidentified	unidentified	unidentified	0.109	0.202	0.109	0.258			0.204	
Dothideomycetes	Pleosporales	unidentified	unidentified	unidentified	0.147		0.147					
Dothideomycetes	Pleosporales	unidentified	unidentified	unidentified	0.033		0.033	0.427				
Dothideomycetes	Pleosporales	unidentified	unidentified	unidentified	0.029	0.315	0.029					
Dothideomycetes	Pleosporales	unidentified	unidentified	unidentified		0.315						

Dothideomycetes	Pleosporales	unidentified	unidentified	unidentified	0.09		0.09		0.035	0.074	0.199	
Dothideomycetes	Pleosporales	unidentified	unidentified	unidentified	0.052		0.052					
Dothideomycetes	Pleosporales	unidentified	unidentified	unidentified		0.127						
Dothideomycetes	Pleosporales	unidentified	unidentified	unidentified		0.067						
Dothideomycetes	Pleosporales	unidentified	unidentified	unidentified	0.019		0.019		0.012			
Dothideomycetes	Pleosporales	unidentified	unidentified	unidentified		0.037						
Dothideomycetes	Pleosporales	unidentified	unidentified	unidentified	0.01		0.01				0.048	
Dothideomycetes	Pleosporales	unidentified	unidentified	unidentified	0.01		0.01					
Dothideomycetes	Pleosporales	unidentified	unidentified	unidentified		0.022						
Dothideomycetes	Pleosporales	unidentified	unidentified	unidentified							0.048	
Dothideomycetes	Pleosporales	unidentified	unidentified	unidentified							0.048	
Dothideomycetes	Pleosporales	unidentified	unidentified	unidentified					0.035			
Dothideomycetes	Pleosporales	unidentified	unidentified	unidentified							0.016	
Dothideomycetes	Pleosporales	unidentified	unidentified	unidentified					0.008			
Dothideomycetes	Pleosporales	unidentified	unidentified	unidentified					0.008			
Dothideomycetes	Pleosporales	unidentified	unidentified	unidentified						0.013		
Dothideomycetes	unidentified	unidentified	unidentified	unidentified	0.105	0.494	0.105					
Dothideomycetes	unidentified	unidentified	unidentified	unidentified				0.893				
Dothideomycetes	unidentified	unidentified	unidentified	unidentified		0.195		0.248				
Dothideomycetes	unidentified	unidentified	unidentified	unidentified	0.081	0.112	0.081					
Dothideomycetes	unidentified	unidentified	unidentified	unidentified				0.258				
Dothideomycetes	unidentified	unidentified	unidentified	unidentified		0.172						
Dothideomycetes	unidentified	unidentified	unidentified	unidentified				0.198				
Dothideomycetes	unidentified	unidentified	unidentified	unidentified				0.189				
Dothideomycetes	unidentified	unidentified	unidentified	unidentified	0.024		0.024		0.019		0.167	
Dothideomycetes	unidentified	unidentified	unidentified	unidentified		0.075						
Dothideomycetes	unidentified	unidentified	unidentified	unidentified	0.014		0.014					
Dothideomycetes	unidentified	unidentified	unidentified	unidentified				0.05				

Dothideomycetes	unidentified	unidentified	unidentified	unidentified		0.022						
Dothideomycetes	unidentified	unidentified	unidentified	unidentified								1.01
Dothideomycetes	unidentified	unidentified	unidentified	unidentified								0.909
Dothideomycetes	unidentified	unidentified	unidentified	unidentified						0.196		
Dothideomycetes	unidentified	unidentified	unidentified	unidentified						0.088		
Dothideomycetes	unidentified	unidentified	unidentified	unidentified					0.043			
Dothideomycetes	unidentified	unidentified	unidentified	unidentified					0.012			
Dothideomycetes	unidentified	unidentified	unidentified	unidentified					0.012			
Dothideomycetes	unidentified	unidentified	unidentified	unidentified						0.013		
Dothideomycetes	Valsariales	Valsariaceae	*Myrmaecium*	*Myrmaecium fulvopruinatum*	0.024		0.024					
Dothideomycetes	Venturiales	Sympoventuriaceae	*Scolecobasidium*	*Scolecobasidium cateniphorum*							0.048	
Dothideomycetes	Venturiales	Sympoventuriaceae	*Scolecobasidium*	*Scolecobasidium constrictum*						0.061		
Eurotiomycetes	Chaetothyriales	Chaetothyriales *Inc. sed.*	*Hyalocladosporiella*	*Hyalocladosporiella tectonae*	0.067	0.157	0.067					
Eurotiomycetes	Chaetothyriales	Chaetothyriales *Inc. sed.*	*Neostrelitziana*	*Neostrelitziana acaciigena*	0.109		0.109	0.159				
Eurotiomycetes	Chaetothyriales	Chaetothyriales *Inc. sed.*	*Strelitziana*	*Strelitziana africana*				0.714				
Eurotiomycetes	Chaetothyriales	Chaetothyriales *Inc. sed.*	*Strelitziana*	*Strelitziana malaysiana*	6.811	0.412	6.811	0.605				
Eurotiomycetes	Chaetothyriales	Chaetothyriales *Inc. sed.*	*Strelitziana*	unidentified	2.576	1.76	2.576	5.041				
Eurotiomycetes	Chaetothyriales	Chaetothyriales *Inc. sed.*	*Strelitziana*	unidentified	0.808		0.808					
Eurotiomycetes	Chaetothyriales	Chaetothyriales *Inc. sed.*	*Strelitziana*	unidentified	0.399		0.399					
Eurotiomycetes	Chaetothyriales	Chaetothyriales *Inc. sed.*	*Strelitziana*	unidentified				0.248				
Eurotiomycetes	Chaetothyriales	Chaetothyriales *Inc. sed.*	*Xenobotrytis*	*Xenobotrytis acaducospora*					0.062	0.587	0.167	
Eurotiomycetes	Chaetothyriales	Cyphellophoraceae	*Cyphellophora*	unidentified	0.352		0.352					

Eurotiomycetes	Chaetothyriales	Cyphellophoraceae	*Cyphellophora*	unidentified	0.124		0.124					
Eurotiomycetes	Chaetothyriales	Cyphellophoraceae	*Cyphellophora*	unidentified		0.075		0.069				
Eurotiomycetes	Chaetothyriales	Cyphellophoraceae	*Cyphellophora*	unidentified				0.079				
Eurotiomycetes	Chaetothyriales	Herpotrichiellaceae	*Cladophialophora*	unidentified				0.03		0.047		
Eurotiomycetes	Chaetothyriales	Herpotrichiellaceae	*Cladophialophora*	unidentified					0.263		0.086	
Eurotiomycetes	Chaetothyriales	Herpotrichiellaceae	*Cladophialophora*	unidentified					0.097			
Eurotiomycetes	Chaetothyriales	Herpotrichiellaceae	*Cladophialophora*	unidentified						0.02		
Eurotiomycetes	Chaetothyriales	Herpotrichiellaceae	*Exophiala*	unidentified							0.043	
Eurotiomycetes	Chaetothyriales	Herpotrichiellaceae	*Melanoctona*	*Melanoctona tectonae*							0.021	
Eurotiomycetes	Chaetothyriales	Herpotrichiellaceae	unidentified	unidentified						12.15		
Eurotiomycetes	Chaetothyriales	Herpotrichiellaceae	unidentified	unidentified						0.594		
Eurotiomycetes	Chaetothyriales	Herpotrichiellaceae	unidentified	unidentified						0.216		
Eurotiomycetes	Chaetothyriales	Herpotrichiellaceae	unidentified	unidentified							0.075	
Eurotiomycetes	Chaetothyriales	Herpotrichiellaceae	unidentified	unidentified						0.02		
Eurotiomycetes	Chaetothyriales	Trichomeriaceae	*Brycekendrickomyces*	*Brycekendrickomyces acaciae*		0.037						
Eurotiomycetes	Chaetothyriales	Trichomeriaceae	*Trichomerium*	*Trichomerium siamensis*		0.037		0.308		0.027		
Eurotiomycetes	Chaetothyriales	unidentified	unidentified	unidentified	0.138		0.138	28.855				
Eurotiomycetes	Chaetothyriales	unidentified	unidentified	unidentified	0.095	0.15	0.095	3.88				
Eurotiomycetes	Chaetothyriales	unidentified	unidentified	unidentified	0.029	1.805	0.029	1.578				
Eurotiomycetes	Chaetothyriales	unidentified	unidentified	unidentified	0.337	0.404	0.337	0.248				
Eurotiomycetes	Chaetothyriales	unidentified	unidentified	unidentified	0.404		0.404					
Eurotiomycetes	Chaetothyriales	unidentified	unidentified	unidentified	0.399		0.399					
Eurotiomycetes	Chaetothyriales	unidentified	unidentified	unidentified	0.157	0.12	0.157					
Eurotiomycetes	Chaetothyriales	unidentified	unidentified	unidentified	0.157	0.082	0.157					
Eurotiomycetes	Chaetothyriales	unidentified	unidentified	unidentified	0.128		0.128					
Eurotiomycetes	Chaetothyriales	unidentified	unidentified	unidentified	0.109		0.109					

Eurotiomycetes	Chaetothyriales	unidentified	unidentified	unidentified	0.081	0.082	0.081			0.074	0.075	
Eurotiomycetes	Chaetothyriales	unidentified	unidentified	unidentified		0.322						
Eurotiomycetes	Chaetothyriales	unidentified	unidentified	unidentified				0.387				
Eurotiomycetes	Chaetothyriales	unidentified	unidentified	unidentified	0.09		0.09					
Eurotiomycetes	Chaetothyriales	unidentified	unidentified	unidentified	0.062		0.062	0.109	0.008			
Eurotiomycetes	Chaetothyriales	unidentified	unidentified	unidentified	0.019	0.202	0.019					
Eurotiomycetes	Chaetothyriales	unidentified	unidentified	unidentified				0.218				
Eurotiomycetes	Chaetothyriales	unidentified	unidentified	unidentified		0.045		0.129				
Eurotiomycetes	Chaetothyriales	unidentified	unidentified	unidentified				0.179				
Eurotiomycetes	Chaetothyriales	unidentified	unidentified	unidentified	0.038		0.038					
Eurotiomycetes	Chaetothyriales	unidentified	unidentified	unidentified				0.159				
Eurotiomycetes	Chaetothyriales	unidentified	unidentified	unidentified		0.097						
Eurotiomycetes	Chaetothyriales	unidentified	unidentified	unidentified				0.129				
Eurotiomycetes	Chaetothyriales	unidentified	unidentified	unidentified		0.09						
Eurotiomycetes	Chaetothyriales	unidentified	unidentified	unidentified				0.109				
Eurotiomycetes	Chaetothyriales	unidentified	unidentified	unidentified	0.024		0.024					
Eurotiomycetes	Chaetothyriales	unidentified	unidentified	unidentified	0.024		0.024					
Eurotiomycetes	Chaetothyriales	unidentified	unidentified	unidentified		0.067						
Eurotiomycetes	Chaetothyriales	unidentified	unidentified	unidentified	0.019		0.019					
Eurotiomycetes	Chaetothyriales	unidentified	unidentified	unidentified				0.079				
Eurotiomycetes	Chaetothyriales	unidentified	unidentified	unidentified				0.069				
Eurotiomycetes	Chaetothyriales	unidentified	unidentified	unidentified		0.045						
Eurotiomycetes	Chaetothyriales	unidentified	unidentified	unidentified		0.045						
Eurotiomycetes	Chaetothyriales	unidentified	unidentified	unidentified		0.045						
Eurotiomycetes	Chaetothyriales	unidentified	unidentified	unidentified				0.06				
Eurotiomycetes	Chaetothyriales	unidentified	unidentified	unidentified		0.037						
Eurotiomycetes	Chaetothyriales	unidentified	unidentified	unidentified		0.03						
Eurotiomycetes	Chaetothyriales	unidentified	unidentified	unidentified				0.03				

Eurotiomycetes	Chaetothyriales	unidentified	unidentified	unidentified		0.015				0.169	0.269	
Eurotiomycetes	Chaetothyriales	unidentified	unidentified	unidentified						0.513		
Eurotiomycetes	Chaetothyriales	unidentified	unidentified	unidentified					0.147	0.223		
Eurotiomycetes	Chaetothyriales	unidentified	unidentified	unidentified						0.277		
Eurotiomycetes	Chaetothyriales	unidentified	unidentified	unidentified						0.216		
Eurotiomycetes	Chaetothyriales	unidentified	unidentified	unidentified							0.167	
Eurotiomycetes	Chaetothyriales	unidentified	unidentified	unidentified					0.108			
Eurotiomycetes	Chaetothyriales	unidentified	unidentified	unidentified							0.107	
Eurotiomycetes	Chaetothyriales	unidentified	unidentified	unidentified					0.073			
Eurotiomycetes	Chaetothyriales	unidentified	unidentified	unidentified						0.108		
Eurotiomycetes	Chaetothyriales	unidentified	unidentified	unidentified							0.086	
Eurotiomycetes	Chaetothyriales	unidentified	unidentified	unidentified						0.108		
Eurotiomycetes	Chaetothyriales	unidentified	unidentified	unidentified					0.058			
Eurotiomycetes	Chaetothyriales	unidentified	unidentified	unidentified						0.094		
Eurotiomycetes	Chaetothyriales	unidentified	unidentified	unidentified							0.075	
Eurotiomycetes	Chaetothyriales	unidentified	unidentified	unidentified							0.075	
Eurotiomycetes	Chaetothyriales	unidentified	unidentified	unidentified						0.088		
Eurotiomycetes	Chaetothyriales	unidentified	unidentified	unidentified							0.048	
Eurotiomycetes	Chaetothyriales	unidentified	unidentified	unidentified					0.035			
Eurotiomycetes	Chaetothyriales	unidentified	unidentified	unidentified							0.043	
Eurotiomycetes	Chaetothyriales	unidentified	unidentified	unidentified						0.047		
Eurotiomycetes	Chaetothyriales	unidentified	unidentified	unidentified						0.04		
Eurotiomycetes	Chaetothyriales	unidentified	unidentified	unidentified					0.023			
Eurotiomycetes	Chaetothyriales	unidentified	unidentified	unidentified						0.034		
Eurotiomycetes	Chaetothyriales	unidentified	unidentified	unidentified						0.034		
Eurotiomycetes	Chaetothyriales	unidentified	unidentified	unidentified						0.027		
Eurotiomycetes	Chaetothyriales	unidentified	unidentified	unidentified					0.012			
Eurotiomycetes	Chaetothyriales	unidentified	unidentified	unidentified							0.016	

Eurotiomycetes	Chaetothyriales	unidentified	unidentified	unidentified							0.016	
Eurotiomycetes	Chaetothyriales	unidentified	unidentified	unidentified					0.012			
Eurotiomycetes	Eurotiales	Aspergillaceae	*Aspergillus*	*Aspergillus aculeatus*							0.027	
Eurotiomycetes	Eurotiales	Aspergillaceae	*Aspergillus*	*Aspergillus carbonarius*		0.045					0.118	
Eurotiomycetes	Eurotiales	Aspergillaceae	*Aspergillus*	*Aspergillus eburneocremeus*						0.25	0.054	
Eurotiomycetes	Eurotiales	Aspergillaceae	*Aspergillus*	*Aspergillus flavus*							0.145	
Eurotiomycetes	Eurotiales	Aspergillaceae	*Aspergillus*	*Aspergillus nidulans*								0.152
Eurotiomycetes	Eurotiales	Aspergillaceae	*Aspergillus*	*Aspergillus sclerotiorum*						0.121	0.081	
Eurotiomycetes	Eurotiales	Aspergillaceae	*Aspergillus*	*Aspergillus tamarii*					0.043	0.02	0.129	
Eurotiomycetes	Eurotiales	Aspergillaceae	*Aspergillus*	unidentified							6.118	
Eurotiomycetes	Eurotiales	Aspergillaceae	*Aspergillus*	unidentified							0.021	
Eurotiomycetes	Eurotiales	Aspergillaceae	*Penicillium*	unidentified						0.567		
Eurotiomycetes	Eurotiales	Aspergillaceae	*Penicillium*	unidentified					0.108			
Eurotiomycetes	Eurotiales	Aspergillaceae	*Penicillium*	unidentified							0.15	
Eurotiomycetes	Eurotiales	Aspergillaceae	*Penicillium*	unidentified					0.066	0.067		
Eurotiomycetes	Eurotiales	Aspergillaceae	*Penicillium*	unidentified						0.115		
Eurotiomycetes	Eurotiales	Aspergillaceae	*Penicillium*	unidentified							0.07	
Eurotiomycetes	Eurotiales	Aspergillaceae	*Penicillium*	unidentified							0.059	
Eurotiomycetes	Eurotiales	Aspergillaceae	*Penicillium*	unidentified						0.067		
Eurotiomycetes	Eurotiales	Aspergillaceae	*Penicillium*	unidentified								0.227
Eurotiomycetes	Eurotiales	Aspergillaceae	unidentified	unidentified								0.581
Eurotiomycetes	Eurotiales	Aspergillaceae	unidentified	unidentified							0.07	0.152
Eurotiomycetes	Eurotiales	Aspergillaceae	unidentified	unidentified							0.054	
Eurotiomycetes	Eurotiales	Aspergillaceae	unidentified	unidentified						0.047		
Eurotiomycetes	Eurotiales	Trichocomaceae	*Talaromyces*	*Talaromyces aculeatus*					0.035		0.129	

Eurotiomycetes	Eurotiales	Trichocomaceae	*Talaromyces*	*Talaromyces minioluteus*								1.465
Eurotiomycetes	Eurotiales	Trichocomaceae	*Talaromyces*	unidentified							0.064	
Eurotiomycetes	Eurotiales	Trichocomaceae	unidentified	unidentified							0.032	
Eurotiomycetes	Eurotiales	unidentified	unidentified	unidentified					0.085			
Eurotiomycetes	Onygenales	unidentified	unidentified	unidentified					0.027			
Eurotiomycetes	unidentified	unidentified	unidentified	unidentified						0.074		
Eurotiomycetes	unidentified	unidentified	unidentified	unidentified						0.054		
Lecanoromycetes	Caliciales	Physciaceae	*Physcia*	*Physcia atrostriata*				0.03				
Lecanoromycetes	Lecanorales	Ectolechiaceae	*Calopadia*	*Calopadia foliicola*	0.38		0.38	0.06				
Lecanoromycetes	Lecanorales	Ramalinaceae	unidentified	unidentified	0.038		0.038					
Leotiomycetes	Helotiales	Dermateaceae	*Trimmatostroma*	*Trimmatostroma cordae*	0.057		0.057	0.109				
Leotiomycetes	Helotiales	Helotiaceae	*Articulospora*	unidentified		0.127						
Leotiomycetes	Helotiales	Helotiaceae	*Articulospora*	unidentified	0.024		0.024	0.06				
Leotiomycetes	Helotiales	Helotiaceae	*Articulospora*	unidentified				0.069				
Leotiomycetes	Helotiales	Rutstroemiaceae	*Lambertella*	unidentified				0.03				
Leotiomycetes	Helotiales	unidentified	unidentified	unidentified		0.112						
Leotiomycetes	Helotiales	unidentified	unidentified	unidentified							0.301	
Leotiomycetes	Leotiomycetes *Inc. sed.*	Leotiomycetes *Inc. sed.*	*Scytalidium*	unidentified					0.081			
Leotiomycetes	Leotiomycetes *Inc. sed.*	Leotiomycetes *Inc. sed.*	*Scytalidium*	unidentified							0.086	
Leotiomycetes	unidentified	unidentified	unidentified	unidentified	0.024		0.024					
Leotiomycetes	unidentified	unidentified	unidentified	unidentified		0.022						
Leotiomycetes	unidentified	unidentified	unidentified	unidentified					0.178			
Leotiomycetes	unidentified	unidentified	unidentified	unidentified					0.135			
Leotiomycetes	unidentified	unidentified	unidentified	unidentified					0.081			
Leotiomycetes	unidentified	unidentified	unidentified	unidentified							0.064	
Orbiliomycetes	Orbiliales	Orbiliaceae	*Dactylella*	*Dactylella clavata*						0.04		

Orbiliomycetes	Orbiliales	Orbiliaceae	*Dactylellina*	*Dactylellina cionopaga*					0.05		0.081	
Orbiliomycetes	Orbiliales	Orbiliaceae	*Orbilia*	*Orbilia auricolor*					0.023		0.075	
Orbiliomycetes	Orbiliales	Orbiliaceae	*Orbilia*	unidentified		0.022						
Orbiliomycetes	Orbiliales	Orbiliaceae	*Orbilia*	unidentified							0.252	
Orbiliomycetes	Orbiliales	Orbiliaceae	*Orbilia*	unidentified							0.177	
Orbiliomycetes	Orbiliales	Orbiliaceae	*Orbilia*	unidentified						0.047		
Orbiliomycetes	Orbiliales	Orbiliaceae	unidentified	unidentified							0.15	0.379
Orbiliomycetes	Orbiliales	unidentified	unidentified	unidentified		0.082			0.031			
Orbiliomycetes	Orbiliales	unidentified	unidentified	unidentified				0.02				
Orbiliomycetes	Orbiliales	unidentified	unidentified	unidentified							0.043	
Orbiliomycetes	unidentified	unidentified	unidentified	unidentified						0.027		
Pezizomycetes	Pezizales	Pezizaceae	*Mattirolomyces*	*Mattirolomyces mexicanus*							3.599	
Pezizomycetes	Pezizales	Pezizaceae	unidentified	unidentified							0.048	
Pezizomycetes	Pezizales	Pyronemataceae	unidentified	unidentified					0.008			
Pezizomycetes	Pezizales	unidentified	unidentified	unidentified						0.02		
Saccharomycetes	Saccharomycetales	Debaryomycetaceae	*Schwanniomyces*	*Schwanniomyces pseudopolymorphus*							0.107	0.152
Saccharomycetes	Saccharomycetales	Saccharomycetales *Inc. sed.*	*Candida*	unidentified							0.064	
Sordariomycetes	Branch06	unidentified	unidentified	unidentified							0.107	
Sordariomycetes	Branch06	unidentified	unidentified	unidentified					0.058			
Sordariomycetes	Branch06	unidentified	unidentified	unidentified							0.07	
Sordariomycetes	Branch06	unidentified	unidentified	unidentified						0.074		
Sordariomycetes	Branch06	unidentified	unidentified	unidentified								0.227
Sordariomycetes	Branch06	unidentified	unidentified	unidentified							0.027	
Sordariomycetes	Diaporthales	Schizoparmaceae	*Coniella*	unidentified				0.228				
Sordariomycetes	Diaporthales	unidentified	unidentified	unidentified				0.139				

Sordariomycetes	Glomerellales	Glomerellaceae	*Colletotrichum*	*Colletotrichum gloeosporioides*	0.228		0.228	0.109				
Sordariomycetes	Glomerellales	Glomerellaceae	*Colletotrichum*	*Colletotrichum truncatum*							0.07	
Sordariomycetes	Glomerellales	Glomerellaceae	*Colletotrichum*	unidentified	0.052	0.157	0.052					
Sordariomycetes	Glomerellales	Glomerellaceae	*Colletotrichum*	unidentified	0.062		0.062					
Sordariomycetes	Glomerellales	Glomerellaceae	*Colletotrichum*	unidentified				0.079				
Sordariomycetes	Glomerellales	Glomerellaceae	*Colletotrichum*	unidentified					0.031			
Sordariomycetes	Glomerellales	Plectosphaerellaceae	unidentified	unidentified		0.022						
Sordariomycetes	Hypocreales	Bionectriaceae	*Hydropisphaera*	unidentified							0.075	
Sordariomycetes	Hypocreales	Clavicipitaceae	unidentified	unidentified					0.019			
Sordariomycetes	Hypocreales	Cordycipitaceae	*Beauveria*	unidentified							0.011	
Sordariomycetes	Hypocreales	Cordycipitaceae	*Simplicillium*	unidentified	0.033		0.033					
Sordariomycetes	Hypocreales	Hypocreaceae	*Trichoderma*	*Trichoderma citrinoviride*					0.039			
Sordariomycetes	Hypocreales	Hypocreaceae	*Trichoderma*	*Trichoderma longibrachiatum*							0.016	2.096
Sordariomycetes	Hypocreales	Hypocreaceae	*Trichoderma*	*Trichoderma virens*							0.059	1.086
Sordariomycetes	Hypocreales	Hypocreaceae	*Trichoderma*	unidentified						0.189		
Sordariomycetes	Hypocreales	Hypocreaceae	*Trichoderma*	unidentified					0.023			
Sordariomycetes	Hypocreales	Hypocreales *Inc. sed.*	*Acremonium*	*Acremonium hennebertii*							0.199	
Sordariomycetes	Hypocreales	Hypocreales *Inc. sed.*	*Solheimia*	*Solheimia costispora*	0.014	0.037	0.014		0.054		0.032	
Sordariomycetes	Hypocreales	Nectriaceae	*Albonectria*	*Albonectria rigidiuscula*	0.086		0.086			0.263		
Sordariomycetes	Hypocreales	Nectriaceae	*Fusarium*	*Fusarium brachygibbosum*					0.043			
Sordariomycetes	Hypocreales	Nectriaceae	*Fusarium*	*Fusarium solani*					0.572	0.405	1.96	
Sordariomycetes	Hypocreales	Nectriaceae	*Fusarium*	unidentified						0.25		
Sordariomycetes	Hypocreales	Nectriaceae	*Nectria*	*Nectria bactridioides*	0.147		0.147				0.118	

Sordariomycetes	Hypocreales	Nectriaceae	*Stylonectria*	unidentified						0.034		
Sordariomycetes	Hypocreales	Nectriaceae	*Thelonectria*	*Thelonectria olida*							0.027	
Sordariomycetes	Hypocreales	Nectriaceae	unidentified	unidentified					0.104			
Sordariomycetes	Hypocreales	Ophiocordycipitaceae	*Hyaloseta*	*Hyaloseta nolinae*					0.012			
Sordariomycetes	Hypocreales	Ophiocordycipitaceae	unidentified	unidentified						0.074		
Sordariomycetes	Hypocreales	Stachybotryaceae	*Myrothecium*	*Myrothecium gramineum*	0.071		0.071	0.139				
Sordariomycetes	Hypocreales	Stachybotryaceae	*Myrothecium*	unidentified						0.081		
Sordariomycetes	Hypocreales	Stachybotryaceae	*Paramyrothecium*	*Paramyrothecium roridum*	0.062		0.062		0.112		0.167	
Sordariomycetes	Hypocreales	Stachybotryaceae	*Smaragdiniseta*	*Smaragdiniseta bisetosa*						0.034		
Sordariomycetes	Hypocreales	Stachybotryaceae	*Stachybotrys*	*Stachybotrys chartarum*					0.046		0.129	
Sordariomycetes	Hypocreales	Stachybotryaceae	*Stachybotrys*	unidentified	0.086		0.086					
Sordariomycetes	Hypocreales	Stachybotryaceae	unidentified	unidentified						0.067		
Sordariomycetes	Hypocreales	unidentified	unidentified	unidentified	0.195	1.386	0.195	0.466		0.067		
Sordariomycetes	Hypocreales	unidentified	unidentified	unidentified		0.427		1.072				
Sordariomycetes	Hypocreales	unidentified	unidentified	unidentified		0.869						
Sordariomycetes	Hypocreales	unidentified	unidentified	unidentified	0.105		0.105					
Sordariomycetes	Hypocreales	unidentified	unidentified	unidentified	0.1		0.1					
Sordariomycetes	Hypocreales	unidentified	unidentified	unidentified		0.225						
Sordariomycetes	Hypocreales	unidentified	unidentified	unidentified		0.202						
Sordariomycetes	Hypocreales	unidentified	unidentified	unidentified	0.024	0.082	0.024					
Sordariomycetes	Hypocreales	unidentified	unidentified	unidentified		0.15						
Sordariomycetes	Hypocreales	unidentified	unidentified	unidentified		0.127						
Sordariomycetes	Hypocreales	unidentified	unidentified	unidentified	0.029		0.029					
Sordariomycetes	Hypocreales	unidentified	unidentified	unidentified	0.029		0.029					
Sordariomycetes	Hypocreales	unidentified	unidentified	unidentified	0.029		0.029					

Sordariomycetes	Hypocreales	unidentified	unidentified	unidentified	0.024		0.024					
Sordariomycetes	Hypocreales	unidentified	unidentified	unidentified		0.067						
Sordariomycetes	Hypocreales	unidentified	unidentified	unidentified				0.089				
Sordariomycetes	Hypocreales	unidentified	unidentified	unidentified		0.052						
Sordariomycetes	Hypocreales	unidentified	unidentified	unidentified	0.014		0.014					
Sordariomycetes	Hypocreales	unidentified	unidentified	unidentified		0.045						
Sordariomycetes	Hypocreales	unidentified	unidentified	unidentified		0.045						
Sordariomycetes	Hypocreales	unidentified	unidentified	unidentified		0.045						
Sordariomycetes	Hypocreales	unidentified	unidentified	unidentified		0.037						
Sordariomycetes	Hypocreales	unidentified	unidentified	unidentified		0.022						
Sordariomycetes	Hypocreales	unidentified	unidentified	unidentified					0.263		0.682	
Sordariomycetes	Hypocreales	unidentified	unidentified	unidentified					0.313		0.424	
Sordariomycetes	Hypocreales	unidentified	unidentified	unidentified						0.729	0.22	
Sordariomycetes	Hypocreales	unidentified	unidentified	unidentified						0.513		
Sordariomycetes	Hypocreales	unidentified	unidentified	unidentified							0.317	
Sordariomycetes	Hypocreales	unidentified	unidentified	unidentified						0.034	0.285	
Sordariomycetes	Hypocreales	unidentified	unidentified	unidentified							0.285	
Sordariomycetes	Hypocreales	unidentified	unidentified	unidentified							0.118	
Sordariomycetes	Hypocreales	unidentified	unidentified	unidentified						0.135		
Sordariomycetes	Hypocreales	unidentified	unidentified	unidentified							0.086	
Sordariomycetes	Hypocreales	unidentified	unidentified	unidentified							0.081	
Sordariomycetes	Hypocreales	unidentified	unidentified	unidentified						0.094		
Sordariomycetes	Hypocreales	unidentified	unidentified	unidentified						0.074		
Sordariomycetes	Hypocreales	unidentified	unidentified	unidentified							0.048	
Sordariomycetes	Hypocreales	unidentified	unidentified	unidentified							0.048	
Sordariomycetes	Hypocreales	unidentified	unidentified	unidentified						0.061		
Sordariomycetes	Hypocreales	unidentified	unidentified	unidentified					0.031			
Sordariomycetes	Hypocreales	unidentified	unidentified	unidentified					0.027			

Sordariomycetes	Hypocreales	unidentified	unidentified	unidentified					0.027			
Sordariomycetes	Hypocreales	unidentified	unidentified	unidentified						0.04		
Sordariomycetes	Hypocreales	unidentified	unidentified	unidentified					0.019			
Sordariomycetes	Magnaporthales	Magnaporthaceae	*Mycoleptodiscus*	unidentified						0.601		
Sordariomycetes	Microascales	Microascaceae	*Pseudallescheria*	unidentified								0.429
Sordariomycetes	Microascales	Microascaceae	*Pseudallescheria*	unidentified						0.027		
Sordariomycetes	Myrmecridiales	Myrmecridiaceae	*Myrmecridium*	*Myrmecridium phragmitis*		0.075						
Sordariomycetes	Pleurotheciales	Pleurotheciaceae	*Phaeoisaria*	*Phaeoisaria sedimenticola*							0.054	
Sordariomycetes	Pleurotheciales	Pleurotheciaceae	*Phaeoisaria*	unidentified						0.013		
Sordariomycetes	Pleurotheciales	Pleurotheciaceae	*Pleurotheciella*	*Pleurotheciella uniseptata*					0.046	0.02		
Sordariomycetes	Savoryellales	unidentified	unidentified	unidentified						0.04		
Sordariomycetes	Sordariales	Chaetomiaceae	*Chaetomium*	unidentified					0.054	1.39		
Sordariomycetes	Sordariales	Chaetomiaceae	*Chaetomium*	unidentified							0.129	
Sordariomycetes	Sordariales	Chaetomiaceae	*Chaetomium*	unidentified					0.081			
Sordariomycetes	Sordariales	Chaetomiaceae	*Chaetomium*	unidentified					0.027		0.027	
Sordariomycetes	Sordariales	Chaetomiaceae	*Chaetomium*	unidentified						0.067		
Sordariomycetes	Sordariales	Chaetomiaceae	*Thielavia*	unidentified						0.25		
Sordariomycetes	Sordariales	Chaetomiaceae	unidentified	unidentified					1.446	2.133	0.295	
Sordariomycetes	Sordariales	Chaetomiaceae	unidentified	unidentified					0.97		2.095	
Sordariomycetes	Sordariales	Chaetomiaceae	unidentified	unidentified							0.124	
Sordariomycetes	Sordariales	Lasiosphaeriaceae	unidentified	unidentified					0.158			
Sordariomycetes	Sordariales	Sordariaceae	*Gelasinospora*	*Gelasinospora cratophora*					0.085			
Sordariomycetes	Sordariales	Sordariales *Inc. sed.*	*Monosporascus*	*Monosporascus cannonballus*	0.124		0.124			0.034	0.274	
Sordariomycetes	Sordariales	unidentified	unidentified	unidentified					0.023			
Sordariomycetes	Sordariomycetes *Inc. sed.*	Sordariomycetes *Inc. sed.*	*Ciliochorella*	unidentified					0.062			

Sordariomycetes	Trichosphaeriales	Trichosphaeriaceae	*Nigrospora*	*Nigrospora oryzae*	0.057		0.057	0.079				22.076
Sordariomycetes	unidentified	unidentified	unidentified	unidentified				2.024				
Sordariomycetes	unidentified	unidentified	unidentified	unidentified	0.128	0.105	0.128			0.101		
Sordariomycetes	unidentified	unidentified	unidentified	unidentified	0.119		0.119					
Sordariomycetes	unidentified	unidentified	unidentified	unidentified	0.081		0.081					
Sordariomycetes	unidentified	unidentified	unidentified	unidentified				0.089				
Sordariomycetes	unidentified	unidentified	unidentified	unidentified	0.019		0.019					
Sordariomycetes	unidentified	unidentified	unidentified	unidentified		0.06						
Sordariomycetes	unidentified	unidentified	unidentified	unidentified	0.014		0.014					
Sordariomycetes	unidentified	unidentified	unidentified	unidentified				0.03				
Sordariomycetes	unidentified	unidentified	unidentified	unidentified				0.03				
Sordariomycetes	unidentified	unidentified	unidentified	unidentified		0.015						
Sordariomycetes	unidentified	unidentified	unidentified	unidentified					0.081			
Sordariomycetes	unidentified	unidentified	unidentified	unidentified					0.077			
Sordariomycetes	unidentified	unidentified	unidentified	unidentified							0.107	
Sordariomycetes	unidentified	unidentified	unidentified	unidentified					0.073			
Sordariomycetes	unidentified	unidentified	unidentified	unidentified						0.121		
Sordariomycetes	unidentified	unidentified	unidentified	unidentified							0.091	
Sordariomycetes	unidentified	unidentified	unidentified	unidentified					0.046			
Sordariomycetes	unidentified	unidentified	unidentified	unidentified							0.059	
Sordariomycetes	unidentified	unidentified	unidentified	unidentified					0.035			
Sordariomycetes	unidentified	unidentified	unidentified	unidentified						0.054		
Sordariomycetes	unidentified	unidentified	unidentified	unidentified					0.027			
Sordariomycetes	unidentified	unidentified	unidentified	unidentified						0.047		
Sordariomycetes	unidentified	unidentified	unidentified	unidentified							0.038	
Sordariomycetes	unidentified	unidentified	unidentified	unidentified								0.126
Sordariomycetes	unidentified	unidentified	unidentified	unidentified						0.034		
Sordariomycetes	unidentified	unidentified	unidentified	unidentified							0.021	

Sordariomycetes	unidentified	unidentified	unidentified	unidentified						0.02		
Sordariomycetes	unidentified	unidentified	unidentified	unidentified							0.011	
Sordariomycetes	Xylariales	Amphisphaeriaceae	*Lepteutypa*	*Lepteutypa sambuci*							0.038	
Sordariomycetes	Xylariales	Bartaliniaceae	*Bartalinia*	unidentified					0.031			
Sordariomycetes	Xylariales	Beltraniaceae	*Beltraniella*	unidentified						0.121		
Sordariomycetes	Xylariales	Beltraniaceae	*Beltraniella*	unidentified					0.054			
Sordariomycetes	Xylariales	Diatrypaceae	*Peroneutypa*	*Peroneutypa alsophila*				0.02				
Sordariomycetes	Xylariales	Diatrypaceae	*Peroneutypa*	*Peroneutypa scoparia*				0.099				
Sordariomycetes	Xylariales	Sporocadaceae	*Pestalotiopsis*	*Pestalotiopsis microspora*						0.054		
Sordariomycetes	Xylariales	Sporocadaceae	*Pestalotiopsis*	unidentified	0.223	0.532	0.223			0.094		
Sordariomycetes	Xylariales	Sporocadaceae	*Pestalotiopsis*	unidentified				0.06				
Sordariomycetes	Xylariales	unidentified	unidentified	unidentified	0.062		0.062					
Sordariomycetes	Xylariales	unidentified	unidentified	unidentified	0.057		0.057					
Sordariomycetes	Xylariales	unidentified	unidentified	unidentified	0.048		0.048					
Sordariomycetes	Xylariales	unidentified	unidentified	unidentified		0.075						
Sordariomycetes	Xylariales	unidentified	unidentified	unidentified		0.067						
Sordariomycetes	Xylariales	unidentified	unidentified	unidentified	0.014		0.014					
Sordariomycetes	Xylariales	unidentified	unidentified	unidentified				0.04				
Sordariomycetes	Xylariales	unidentified	unidentified	unidentified							0.091	
Sordariomycetes	Xylariales	unidentified	unidentified	unidentified						0.108		
Sordariomycetes	Xylariales	unidentified	unidentified	unidentified					0.043			
Sordariomycetes	Xylariales	Xylariaceae	*Daldinia*	unidentified	0.105		0.105					
Sordariomycetes	Xylariales	Xylariaceae	*Hypoxylon*	*Hypoxylon haematostroma*	0.062	0.06	0.062		0.027			
Sordariomycetes	Xylariales	Xylariaceae	*Hypoxylon*	*Hypoxylon monticulosum*				0.06				
Sordariomycetes	Xylariales	Xylariaceae	*Hypoxylon*	*Hypoxylon musceum*	0.019	0.067	0.019	0.069				

Sordariomycetes	Xylariales	Xylariaceae	*Hypoxylon*	unidentified	0.086		0.086					
Sordariomycetes	Xylariales	Xylariaceae	*Hypoxylon*	unidentified	0.052	0.045	0.052					
Sordariomycetes	Xylariales	Xylariaceae	*Hypoxylon*	unidentified	0.043		0.043					
Sordariomycetes	Xylariales	Xylariaceae	*Hypoxylon*	unidentified				0.159				
Sordariomycetes	Xylariales	Xylariaceae	*Hypoxylon*	unidentified	0.019		0.019					
Sordariomycetes	Xylariales	Xylariaceae	unidentified	unidentified	0.556	0.457	0.556	0.129	0.023	0.054		
Sordariomycetes	Xylariales	Xylariaceae	unidentified	unidentified	0.057		0.057					
Sordariomycetes	Xylariales	Xylariaceae	unidentified	unidentified				0.099				
Sordariomycetes	Xylariales	Xylariaceae	unidentified	unidentified		0.022						
Sordariomycetes	Xylariales	Xylariaceae	unidentified	unidentified					0.012	0.013	0.081	
Sordariomycetes	Xylariales	Xylariaceae	unidentified	unidentified					0.031			
Sordariomycetes	Xylariales	Xylariaceae	*Xylaria*	unidentified	0.052		0.052					
Sordariomycetes	Xylariales	Xylariaceae	*Xylaria*	unidentified		0.052						
Sordariomycetes	Xylariales	Xylariaceae	*Xylaria*	unidentified	0.014		0.014					
Sordariomycetes	Xylariales	Xylariaceae	*Xylaria*	unidentified				0.04				
Sordariomycetes	Xylariales	Xylariaceae	*Xylaria*	unidentified							0.258	
Sordariomycetes	Xylariales	Xylariaceae	*Xylaria*	*Xylaria apiculata*					1.14			
Sordariomycetes	Xylariales	Xylariaceae	*Xylaria*	*Xylaria longipes*	0.033		0.033	0.06				
Sordariomycetes	Xylariales	Xylariales *Inc. sed.*	*Castanediella*	*Castanediella ramosa*							0.124	
Sordariomycetes	Xylariales	Xylariales *Inc. sed.*	*Robillarda*	unidentified						0.094		
unidentified	unidentified	unidentified	unidentified	unidentified	1.041	0.112	1.041					
unidentified	unidentified	unidentified	unidentified	unidentified	0.456	0.794	0.456					
unidentified	unidentified	unidentified	unidentified	unidentified	0.528	0.322	0.528	0.228				
unidentified	unidentified	unidentified	unidentified	unidentified	0.499	0.292	0.499					
unidentified	unidentified	unidentified	unidentified	unidentified	0.566		0.566					
unidentified	unidentified	unidentified	unidentified	unidentified	0.285	0.135	0.285					
unidentified	unidentified	unidentified	unidentified	unidentified				1.211				

unidentified	unidentified	unidentified	unidentified	unidentified	0.086	0.12	0.086	0.109					
unidentified	unidentified	unidentified	unidentified	unidentified	0.138		0.138						
unidentified	unidentified	unidentified	unidentified	unidentified				0.496					
unidentified	unidentified	unidentified	unidentified	unidentified	0.09	0.045	0.09						
unidentified	unidentified	unidentified	unidentified	unidentified	0.1		0.1						
unidentified	unidentified	unidentified	unidentified	unidentified	0.095		0.095		0.058		0.252		
unidentified	unidentified	unidentified	unidentified	unidentified				0.377					
unidentified	unidentified	unidentified	unidentified	unidentified				0.288	0.201				
unidentified	unidentified	unidentified	unidentified	unidentified	0.029	0.097	0.029						
unidentified	unidentified	unidentified	unidentified	unidentified				0.189					
unidentified	unidentified	unidentified	unidentified	unidentified	0.043		0.043						
unidentified	unidentified	unidentified	unidentified	unidentified		0.112		0.02					
unidentified	unidentified	unidentified	unidentified	unidentified	0.038		0.038						
unidentified	unidentified	unidentified	unidentified	unidentified	0.038		0.038						
unidentified	unidentified	unidentified	unidentified	unidentified	0.038		0.038						
unidentified	unidentified	unidentified	unidentified	unidentified	0.038		0.038						
unidentified	unidentified	unidentified	unidentified	unidentified		0.112							
unidentified	unidentified	unidentified	unidentified	unidentified	0.033		0.033						
unidentified	unidentified	unidentified	unidentified	unidentified	0.033		0.033						
unidentified	unidentified	unidentified	unidentified	unidentified				0.129			0.038		
unidentified	unidentified	unidentified	unidentified	unidentified	0.029		0.029						
unidentified	unidentified	unidentified	unidentified	unidentified				0.089					
unidentified	unidentified	unidentified	unidentified	unidentified	0.019		0.019						
unidentified	unidentified	unidentified	unidentified	unidentified		0.06							
unidentified	unidentified	unidentified	unidentified	unidentified				0.04					
unidentified	unidentified	unidentified	unidentified	unidentified		0.022							
unidentified	unidentified	unidentified	unidentified	unidentified				0.03					
unidentified	unidentified	unidentified	unidentified	unidentified				0.03					

unidentified	unidentified	unidentified	unidentified	unidentified		0.015						
unidentified	unidentified	unidentified	unidentified	unidentified				0.02				
unidentified	unidentified	unidentified	unidentified	unidentified				0.02				
unidentified	unidentified	unidentified	unidentified	unidentified						0.337		
unidentified	unidentified	unidentified	unidentified	unidentified					0.046	0.142		
unidentified	unidentified	unidentified	unidentified	unidentified								0.808
unidentified	unidentified	unidentified	unidentified	unidentified							0.134	
unidentified	unidentified	unidentified	unidentified	unidentified						0.162		
unidentified	unidentified	unidentified	unidentified	unidentified					0.05	0.054	0.011	
unidentified	unidentified	unidentified	unidentified	unidentified						0.142		
unidentified	unidentified	unidentified	unidentified	unidentified							0.097	
unidentified	unidentified	unidentified	unidentified	unidentified					0.046			
unidentified	unidentified	unidentified	unidentified	unidentified					0.039			
unidentified	unidentified	unidentified	unidentified	unidentified						0.061		
unidentified	unidentified	unidentified	unidentified	unidentified						0.061		
unidentified	unidentified	unidentified	unidentified	unidentified					0.023			
unidentified	unidentified	unidentified	unidentified	unidentified						0.034		
unidentified	unidentified	unidentified	unidentified	unidentified						0.034		
unidentified	unidentified	unidentified	unidentified	unidentified							0.027	
unidentified	unidentified	unidentified	unidentified	unidentified						0.027		
unidentified	unidentified	unidentified	unidentified	unidentified					0.012			
unidentified	unidentified	unidentified	unidentified	unidentified						0.02		
unidentified	unidentified	unidentified	unidentified	unidentified							0.016	
unidentified	unidentified	unidentified	unidentified	unidentified							0.016	
unidentified	unidentified	unidentified	unidentified	unidentified					0.012			
**Basidiomycota**												
Agaricomycetes	Agaricales	Agaricaceae	*Agaricus*	*Agaricus subrufescens*				0.04				

Agaricomycetes	Agaricales	Agaricaceae	*Agaricus*	unidentified				0.129				
Agaricomycetes	Agaricales	Agaricaceae	*Agaricus*	unidentified						0.034		
Agaricomycetes	Agaricales	Agaricaceae	*Lepiota*	*Lepiota felina*					0.085			
Agaricomycetes	Agaricales	Agaricaceae	*Lepiota*	*Lepiota flammeotincta*					0.027			
Agaricomycetes	Agaricales	Agaricaceae	*Lepiota*	unidentified	0.01		0.01					
Agaricomycetes	Agaricales	Agaricaceae	*Lepiota*	unidentified						10.996		
Agaricomycetes	Agaricales	Agaricaceae	*Leucoagaricus*	*Leucoagaricus brunnescens*				0.119				
Agaricomycetes	Agaricales	Agaricaceae	*Leucoagaricus*	*Leucoagaricus rubrotinctus*							0.102	
Agaricomycetes	Agaricales	Agaricaceae	*Leucoagaricus*	*Leucoagaricus viriditinctus*							0.054	
Agaricomycetes	Agaricales	Agaricaceae	*Leucoagaricus*	unidentified						0.061		
Agaricomycetes	Agaricales	Agaricaceae	unidentified	unidentified							0.381	
Agaricomycetes	Agaricales	Agaricaceae	unidentified	unidentified							0.193	
Agaricomycetes	Agaricales	Agaricaceae	unidentified	unidentified							0.048	
Agaricomycetes	Agaricales	Agaricaceae	unidentified	unidentified							0.048	
Agaricomycetes	Agaricales	Agaricaceae	unidentified	unidentified					0.027			
Agaricomycetes	Agaricales	Agaricaceae	unidentified	unidentified						0.013		
Agaricomycetes	Agaricales	Bolbitiaceae	*Conocybe*	*Conocybe volvata*						0.027		
Agaricomycetes	Agaricales	Bolbitiaceae	*Panaeolus*	*Panaeolus papilionaceus*						0.013		
Agaricomycetes	Agaricales	Cortinariaceae	*Gymnopilus*	unidentified	0.014		0.014					
Agaricomycetes	Agaricales	Entolomataceae	*Clitopilus*	unidentified				0.139				
Agaricomycetes	Agaricales	Entolomataceae	*Clitopilus*	unidentified	0.01		0.01	0.03				
Agaricomycetes	Agaricales	Entolomataceae	*Clitopilus*	unidentified						0.236		
Agaricomycetes	Agaricales	Entolomataceae	*Clitopilus*	unidentified						0.135		
Agaricomycetes	Agaricales	Entolomataceae	*Clitopilus*	unidentified						0.054		
Agaricomycetes	Agaricales	Hygrophoraceae	*Hygrocybe*	unidentified						0.574		

Agaricomycetes	Agaricales	Lycoperdaceae	*Lycoperdon*	unidentified					0.035			
Agaricomycetes	Agaricales	Lyophyllaceae	*Gerhardtia*	unidentified						0.04		
Agaricomycetes	Agaricales	Marasmiaceae	*Campanella*	unidentified		0.037						
Agaricomycetes	Agaricales	Marasmiaceae	*Marasmius*	*Marasmius corrugatiformis*							0.032	
Agaricomycetes	Agaricales	Marasmiaceae	*Marasmius*	*Marasmius ferruginoides*					0.039			
Agaricomycetes	Agaricales	Marasmiaceae	*Marasmius*	unidentified	0.01		0.01					
Agaricomycetes	Agaricales	Marasmiaceae	*Marasmius*	unidentified				0.02				
Agaricomycetes	Agaricales	Marasmiaceae	*Marasmius*	unidentified						0.128		
Agaricomycetes	Agaricales	Marasmiaceae	*Marasmius*	unidentified						0.054		
Agaricomycetes	Agaricales	Marasmiaceae	*Marasmius*	unidentified						0.013		
Agaricomycetes	Agaricales	Marasmiaceae	*Moniliophthora*	*Moniliophthora canescens*							0.064	
Agaricomycetes	Agaricales	Marasmiaceae	*Moniliophthora*	*Moniliophthora perniciosa*					0.375	0.142		
Agaricomycetes	Agaricales	Marasmiaceae	*Tetrapyrgos*	*Tetrapyrgos nigripes*							0.747	
Agaricomycetes	Agaricales	Marasmiaceae	unidentified	unidentified						0.155		
Agaricomycetes	Agaricales	Marasmiaceae	unidentified	unidentified						0.101		
Agaricomycetes	Agaricales	Omphalotaceae	*Marasmiellus*	*Marasmiellus candidus*	0.01		0.01		0.012	0.634		1.364
Agaricomycetes	Agaricales	Omphalotaceae	*Marasmiellus*	unidentified							0.097	
Agaricomycetes	Agaricales	Omphalotaceae	*Marasmiellus*	unidentified							0.043	
Agaricomycetes	Agaricales	Pluteaceae	*Pluteus*	*Pluteus heteromarginatus*				0.06			0.07	
Agaricomycetes	Agaricales	Pluteaceae	*Pluteus*	unidentified					0.066			
Agaricomycetes	Agaricales	Pluteaceae	*Pluteus*	unidentified						0.054		
Agaricomycetes	Agaricales	Psathyrellaceae	*Coprinellus*	*Coprinellus radians*	0.033		0.033					
Agaricomycetes	Agaricales	Psathyrellaceae	*Coprinellus*	unidentified							0.011	
Agaricomycetes	Agaricales	Psathyrellaceae	*Coprinopsis*	*Coprinopsis clastophylla*				0.169				

Agaricomycetes	Agaricales	Psathyrellaceae	*Coprinopsis*	*Coprinopsis episcopalis*						0.297		
Agaricomycetes	Agaricales	Psathyrellaceae	*Coprinopsis*	unidentified	0.014		0.014					
Agaricomycetes	Agaricales	Psathyrellaceae	*Parasola*	*Parasola kuehneri*							0.011	
Agaricomycetes	Agaricales	Psathyrellaceae	*Psathyrella*	unidentified				0.109				
Agaricomycetes	Agaricales	Psathyrellaceae	*Psathyrella*	unidentified	0.019		0.019					
Agaricomycetes	Agaricales	Psathyrellaceae	*Psathyrella*	unidentified						0.196		
Agaricomycetes	Agaricales	Psathyrellaceae	unidentified	unidentified						0.337		
Agaricomycetes	Agaricales	Psathyrellaceae	unidentified	unidentified						0.256		
Agaricomycetes	Agaricales	Stephanosporaceae	*Cristinia*	unidentified							0.027	
Agaricomycetes	Agaricales	Stephanosporaceae	unidentified	unidentified							0.032	
Agaricomycetes	Agaricales	unidentified	unidentified	unidentified	0.299	0.09	0.299	0.238				
Agaricomycetes	Agaricales	unidentified	unidentified	unidentified	0.019		0.019					
Agaricomycetes	Agaricales	unidentified	unidentified	unidentified						11.32		
Agaricomycetes	Agaricales	unidentified	unidentified	unidentified					0.043	7.722		
Agaricomycetes	Agaricales	unidentified	unidentified	unidentified						1.89		
Agaricomycetes	Agaricales	unidentified	unidentified	unidentified						0.459		
Agaricomycetes	Agaricales	unidentified	unidentified	unidentified					0.263			
Agaricomycetes	Agaricales	unidentified	unidentified	unidentified					0.251			
Agaricomycetes	Agaricales	unidentified	unidentified	unidentified					0.22			
Agaricomycetes	Agaricales	unidentified	unidentified	unidentified					0.143			
Agaricomycetes	Agaricales	unidentified	unidentified	unidentified							0.199	
Agaricomycetes	Agaricales	unidentified	unidentified	unidentified						0.162		
Agaricomycetes	Agaricales	unidentified	unidentified	unidentified					0.073			
Agaricomycetes	Agaricales	unidentified	unidentified	unidentified						0.081		
Agaricomycetes	Agaricales	unidentified	unidentified	unidentified						0.074		
Agaricomycetes	Agaricales	unidentified	unidentified	unidentified							0.043	
Agaricomycetes	Agaricales	unidentified	unidentified	unidentified						0.034		

Agaricomycetes	Agaricales	unidentified	unidentified	unidentified					0.015			
Agaricomycetes	Agaricales	unidentified	unidentified	unidentified							0.021	
Agaricomycetes	Agaricales	unidentified	unidentified	unidentified							0.021	
Agaricomycetes	Auriculariales	Aporpiaceae	*Elmerina*	unidentified				0.02				
Agaricomycetes	Auriculariales	unidentified	unidentified	unidentified					0.108			
Agaricomycetes	Auriculariales	unidentified	unidentified	unidentified							0.124	
Agaricomycetes	Auriculariales	unidentified	unidentified	unidentified							0.043	
Agaricomycetes	Auriculariales	unidentified	unidentified	unidentified						0.013		
Agaricomycetes	Boletales	Boletaceae	unidentified	unidentified				0.06				
Agaricomycetes	Boletales	Sclerodermataceae	*Scleroderma*	unidentified				0.189				
Agaricomycetes	Boletales	unidentified	unidentified	unidentified				0.06				
Agaricomycetes	Cantharellales	Ceratobasidiaceae	*Ceratobasidium*	unidentified		0.24						
Agaricomycetes	Cantharellales	Ceratobasidiaceae	*Ceratobasidium*	unidentified	0.014		0.014					
Agaricomycetes	Cantharellales	Ceratobasidiaceae	*Ceratobasidium*	unidentified							1.423	
Agaricomycetes	Cantharellales	Ceratobasidiaceae	*Thanatephorus*	*Thanatephorus cucumeris*	0.067		0.067				0.016	
Agaricomycetes	Cantharellales	Ceratobasidiaceae	*Thanatephorus*	unidentified						0.58		0.808
Agaricomycetes	Cantharellales	Ceratobasidiaceae	unidentified	unidentified				0.109				
Agaricomycetes	Cantharellales	Ceratobasidiaceae	unidentified	unidentified							1.053	
Agaricomycetes	Cantharellales	Ceratobasidiaceae	unidentified	unidentified					0.541			
Agaricomycetes	Cantharellales	Ceratobasidiaceae	unidentified	unidentified							0.698	
Agaricomycetes	Cantharellales	Ceratobasidiaceae	unidentified	unidentified							0.333	
Agaricomycetes	Cantharellales	Ceratobasidiaceae	unidentified	unidentified						0.101	0.145	
Agaricomycetes	Cantharellales	Ceratobasidiaceae	unidentified	unidentified							0.167	
Agaricomycetes	Cantharellales	Ceratobasidiaceae	unidentified	unidentified						0.135		
Agaricomycetes	Cantharellales	Ceratobasidiaceae	unidentified	unidentified						0.034		0.278
Agaricomycetes	Cantharellales	Ceratobasidiaceae	unidentified	unidentified					0.05			
Agaricomycetes	Cantharellales	Ceratobasidiaceae	unidentified	unidentified					0.043			

Agaricomycetes	Cantharellales	Ceratobasidiaceae	unidentified	unidentified					0.027			
Agaricomycetes	Corticiales	Corticiaceae	*Candelabrochaete*	*Candelabrochaete cirrata*								2.299
Agaricomycetes	Corticiales	Corticiaceae	*Laetisaria*	*Laetisaria lichenicola*				0.208				
Agaricomycetes	Corticiales	Corticiaceae	*Phlebia*	*Phlebia acanthocystis*				0.02				
Agaricomycetes	Corticiales	Corticiaceae	*Phlebia*	unidentified		0.037						
Agaricomycetes	Corticiales	Corticiaceae	*Phlebia*	unidentified				0.04				2.753
Agaricomycetes	Corticiales	Corticiaceae	*Subulicystidium*	*Subulicystidium perlongisporum*					0.046			
Agaricomycetes	Corticiales	Corticiaceae	*Subulicystidium*	unidentified							0.102	
Agaricomycetes	Corticiales	Vuilleminiaceae	*Vuilleminia*	unidentified					0.031			
Agaricomycetes	Geastrales	Geastraceae	*Geastrum*	*Geastrum velutinum*				0.02				
Agaricomycetes	Geastrales	Geastraceae	*Geastrum*	unidentified				0.06				
Agaricomycetes	Geastrales	Geastraceae	*Geastrum*	unidentified						0.614		
Agaricomycetes	Geastrales	Geastraceae	*Geastrum*	unidentified								1.819
Agaricomycetes	Geastrales	Geastraceae	*Geastrum*	unidentified							0.204	
Agaricomycetes	Geastrales	Geastraceae	*Geastrum*	unidentified							0.172	
Agaricomycetes	Geastrales	Geastraceae	*Geastrum*	unidentified							0.043	
Agaricomycetes	Geastrales	Geastraceae	*Geastrum*	unidentified							0.016	
Agaricomycetes	Hymenochaetales	Hymenochaetaceae	*Fuscoporia*	*Fuscoporia torulosa*								0.909
Agaricomycetes	Hymenochaetales	Hymenochaetaceae	*Inonotus*	unidentified		0.052						
Agaricomycetes	Polyporales	Coriolaceae	*Daedalea*	*Daedalea microsticta*	0.024		0.024					
Agaricomycetes	Polyporales	Coriolaceae	unidentified	unidentified								3.284
Agaricomycetes	Polyporales	Coriolaceae	unidentified	unidentified								1.238
Agaricomycetes	Polyporales	Cystostereaceae	*Cystidiodontia*	*Cystidiodontia laminifera*				0.03				
Agaricomycetes	Polyporales	Fomitopsidaceae	*Fomitopsis*	*Fomitopsis feei*							0.032	0.606

Agaricomycetes	Polyporales	Fomitopsidaceae	*Fomitopsis*	unidentified								5.633
Agaricomycetes	Polyporales	Ganodermataceae	*Amauroderma*	unidentified					0.07			
Agaricomycetes	Polyporales	Ganodermataceae	*Ganoderma*	*Ganoderma chalceum*	0.228		0.228	0.149	0.062		0.22	
Agaricomycetes	Polyporales	Ganodermataceae	*Ganoderma*	*Ganoderma lucidum*						0.02		
Agaricomycetes	Polyporales	Ganodermataceae	unidentified	unidentified				0.05				
Agaricomycetes	Polyporales	Phanerochaetaceae	*Efibula*	*Efibula clarkii*								3.94
Agaricomycetes	Polyporales	Phanerochaetaceae	*Phanerochaete*	unidentified		0.03						
Agaricomycetes	Polyporales	Phanerochaetaceae	*Phlebiopsis*	*Phlebiopsis flavidoalba*								1.667
Agaricomycetes	Polyporales	Phanerochaetaceae	*Phlebiopsis*	unidentified								2.021
Agaricomycetes	Polyporales	Phanerochaetaceae	*Phlebiopsis*	unidentified							0.027	
Agaricomycetes	Polyporales	Polyporaceae	*Favolus*	*Favolus grammocephalus*					0.019			1.667
Agaricomycetes	Polyporales	Polyporaceae	*Funalia*	*Funalia caperata*					0.008			
Agaricomycetes	Polyporales	Polyporaceae	*Heliocybe*	*Heliocybe sulcata*					0.031			
Agaricomycetes	Polyporales	Pterulaceae	*Pterula*	*Pterula echo*	0.076	0.082	0.076	0.119				
Agaricomycetes	Polyporales	Schizophyllaceae	*Schizophyllum*	*Schizophyllum commune*								0.253
Agaricomycetes	Polyporales	unidentified	unidentified	unidentified	0.01		0.01					
Agaricomycetes	Polyporales	unidentified	unidentified	unidentified				0.03				
Agaricomycetes	Polyporales	unidentified	unidentified	unidentified				0.02				
Agaricomycetes	Polyporales	unidentified	unidentified	unidentified					0.008			8.74
Agaricomycetes	Polyporales	unidentified	unidentified	unidentified								1.44
Agaricomycetes	Polyporales	unidentified	unidentified	unidentified						0.027		
Agaricomycetes	Russulales	Gloeocystidiellaceae	*Laxitextum*	*Laxitextum bicolor*				0.02				
Agaricomycetes	Russulales	Peniophoraceae	*Peniophora*	unidentified				0.089				
Agaricomycetes	Russulales	Peniophoraceae	*Peniophora*	unidentified				0.04				
Agaricomycetes	Russulales	Peniophoraceae	*Peniophora*	unidentified					0.093		0.247	2.981

Agaricomycetes	Russulales	Peniophoraceae	*Peniophora*	unidentified								2.4
Agaricomycetes	Russulales	Peniophoraceae	*Peniophora*	unidentified								0.631
Agaricomycetes	Russulales	Peniophoraceae	*Peniophora*	unidentified					0.043			
Agaricomycetes	Russulales	Peniophoraceae	*Peniophora*	unidentified					0.039			
Agaricomycetes	Russulales	Peniophoraceae	*Peniophora*	unidentified							0.054	
Agaricomycetes	Russulales	unidentified	unidentified	unidentified				0.129				
Agaricomycetes	Sebacinales	Sebacinaceae	unidentified	unidentified					0.182			
Agaricomycetes	Sebacinales	Sebacinaceae	unidentified	unidentified					0.12			
Agaricomycetes	Sebacinales	Sebacinaceae	unidentified	unidentified						0.027		
Agaricomycetes	Thelephorales	Thelephoraceae	unidentified	unidentified	0.019		0.019					
Agaricomycetes	Thelephorales	Thelephoraceae	unidentified	unidentified		0.015						
Agaricomycetes	Thelephorales	Thelephoraceae	unidentified	unidentified						0.594		
Agaricomycetes	Thelephorales	Thelephoraceae	unidentified	unidentified						0.479		
Agaricomycetes	Thelephorales	Thelephoraceae	unidentified	unidentified						0.081	0.027	
Agaricomycetes	Thelephorales	Thelephoraceae	unidentified	unidentified							0.038	
Agaricomycetes	Thelephorales	Thelephoraceae	unidentified	unidentified					0.015			
Agaricomycetes	Trechisporales	Hydnodontaceae	*Trechispora*	unidentified		0.225						
Agaricomycetes	Trechisporales	Hydnodontaceae	*Trechispora*	unidentified	0.048		0.048					
Agaricomycetes	Trechisporales	Hydnodontaceae	*Trechispora*	unidentified		0.037						
Agaricomycetes	Trechisporales	Hydnodontaceae	*Trechispora*	unidentified	0.01		0.01					
Agaricomycetes	Trechisporales	Hydnodontaceae	*Trechispora*	unidentified						6.52		
Agaricomycetes	Trechisporales	Hydnodontaceae	*Trechispora*	unidentified						6.46		
Agaricomycetes	Trechisporales	unidentified	unidentified	unidentified		0.045						
Agaricomycetes	Trechisporales	unidentified	unidentified	unidentified				0.04				
Agaricomycetes	Trechisporales	unidentified	unidentified	unidentified						0.04		
Agaricomycetes	unidentified	unidentified	unidentified	unidentified	0.033		0.033					
Agaricomycetes	unidentified	unidentified	unidentified	unidentified		0.105						
Agaricomycetes	unidentified	unidentified	unidentified	unidentified				0.079				

Agaricomycetes	unidentified	unidentified	unidentified	unidentified		0.037						
Agaricomycetes	unidentified	unidentified	unidentified	unidentified	0.01		0.01					
Agaricomycetes	unidentified	unidentified	unidentified	unidentified				0.02				
Agaricomycetes	unidentified	unidentified	unidentified	unidentified					42.991		0.376	
Agaricomycetes	unidentified	unidentified	unidentified	unidentified					37.298		0.129	
Agaricomycetes	unidentified	unidentified	unidentified	unidentified							3.889	
Agaricomycetes	unidentified	unidentified	unidentified	unidentified					0.962	0.277	0.709	
Agaricomycetes	unidentified	unidentified	unidentified	unidentified					0.993			
Agaricomycetes	unidentified	unidentified	unidentified	unidentified								5.052
Agaricomycetes	unidentified	unidentified	unidentified	unidentified								3.536
Agaricomycetes	unidentified	unidentified	unidentified	unidentified					0.336			
Agaricomycetes	unidentified	unidentified	unidentified	unidentified							0.279	
Agaricomycetes	unidentified	unidentified	unidentified	unidentified						0.209		
Agaricomycetes	unidentified	unidentified	unidentified	unidentified						0.189		
Agaricomycetes	unidentified	unidentified	unidentified	unidentified							0.07	
Agaricomycetes	unidentified	unidentified	unidentified	unidentified						0.054		
Agaricomycetes	unidentified	unidentified	unidentified	unidentified					0.031			
Agaricomycetes	unidentified	unidentified	unidentified	unidentified					0.023			
Agaricomycetes	unidentified	unidentified	unidentified	unidentified							0.016	
Agaricomycetes	unidentified	unidentified	unidentified	unidentified						0.013		
Agaricostilbomycetes	Agaricostilbales	Agaricostilbaceae	*Sterigmatomyces*	*Sterigmatomyces halophilus*								3.789
Agaricostilbomycetes	Agaricostilbales	Agaricostilbaceae	*Sterigmatomyces*	*Sterigmatomyces halophilus*							0.032	
Geminibasidiomycetes	Geminibasidiales	Geminibasidiaceae	*Geminibasidium*	unidentified							0.07	
unidentified	unidentified	unidentified	unidentified	unidentified	1.445	32.415	1.445	2.887				
unidentified	unidentified	unidentified	unidentified	unidentified	1.217	1.775	1.217	0.05				
unidentified	unidentified	unidentified	unidentified	unidentified	1.369		1.369					

unidentified	unidentified	unidentified	unidentified	unidentified	0.765	0.831	0.765					
unidentified	unidentified	unidentified	unidentified	unidentified		2.726						
unidentified	unidentified	unidentified	unidentified	unidentified		2.674						
unidentified	unidentified	unidentified	unidentified	unidentified	0.29	0.202	0.29	0.595				
unidentified	unidentified	unidentified	unidentified	unidentified		0.839						
unidentified	unidentified	unidentified	unidentified	unidentified	0.048	0.075	0.048	0.258				
unidentified	unidentified	unidentified	unidentified	unidentified		0.322						
unidentified	unidentified	unidentified	unidentified	unidentified	0.09		0.09					
unidentified	unidentified	unidentified	unidentified	unidentified				0.327				
unidentified	unidentified	unidentified	unidentified	unidentified		0.022		0.258				
unidentified	unidentified	unidentified	unidentified	unidentified				0.278				
unidentified	unidentified	unidentified	unidentified	unidentified		0.082						
unidentified	unidentified	unidentified	unidentified	unidentified		0.06						
unidentified	unidentified	unidentified	unidentified	unidentified	0.014		0.014					
unidentified	unidentified	unidentified	unidentified	unidentified	0.014		0.014					
unidentified	unidentified	unidentified	unidentified	unidentified	0.014		0.014					
unidentified	unidentified	unidentified	unidentified	unidentified		0.022						
unidentified	unidentified	unidentified	unidentified	unidentified		0.022						
unidentified	unidentified	unidentified	unidentified	unidentified		0.015						
unidentified	unidentified	unidentified	unidentified	unidentified				0.02				
unidentified	unidentified	unidentified	unidentified	unidentified				0.02				
unidentified	unidentified	unidentified	unidentified	unidentified				0.02				
unidentified	unidentified	unidentified	unidentified	unidentified							27.076	
unidentified	unidentified	unidentified	unidentified	unidentified							21.861	0.278
unidentified	unidentified	unidentified	unidentified	unidentified						5.542		
unidentified	unidentified	unidentified	unidentified	unidentified						3.429		
unidentified	unidentified	unidentified	unidentified	unidentified							1.375	
unidentified	unidentified	unidentified	unidentified	unidentified								3.284

unidentified	unidentified	unidentified	unidentified	unidentified					0.1		0.505	
unidentified	unidentified	unidentified	unidentified	unidentified					0.359			
unidentified	unidentified	unidentified	unidentified	unidentified						0.216		
unidentified	unidentified	unidentified	unidentified	unidentified							0.167	
unidentified	unidentified	unidentified	unidentified	unidentified							0.156	
unidentified	unidentified	unidentified	unidentified	unidentified							0.14	
unidentified	unidentified	unidentified	unidentified	unidentified							0.091	
unidentified	unidentified	unidentified	unidentified	unidentified							0.086	
unidentified	unidentified	unidentified	unidentified	unidentified							0.059	
unidentified	unidentified	unidentified	unidentified	unidentified						0.061		
unidentified	unidentified	unidentified	unidentified	unidentified							0.048	
unidentified	unidentified	unidentified	unidentified	unidentified						0.047		
unidentified	unidentified	unidentified	unidentified	unidentified							0.032	
unidentified	unidentified	unidentified	unidentified	unidentified						0.034		
unidentified	unidentified	unidentified	unidentified	unidentified							0.027	
unidentified	unidentified	unidentified	unidentified	unidentified					0.015			
unidentified	unidentified	unidentified	unidentified	unidentified					0.015			
unidentified	unidentified	unidentified	unidentified	unidentified						0.027		
unidentified	unidentified	unidentified	unidentified	unidentified						0.027		
unidentified	unidentified	unidentified	unidentified	unidentified						0.02		
unidentified	unidentified	unidentified	unidentified	unidentified					0.012			
unidentified	unidentified	unidentified	unidentified	unidentified					0.008			
**Chytridiomycota**												
Rhizophlyctidomycetes	Rhizophlyctidales	Rhizophlyctidaceae	*Rhizophlyctis*	*Rhizophlyctis rosea*							0.032	
Rhizophlyctidomycetes	Rhizophlyctidales	Rhizophlyctidaceae	*Rhizophlyctis*	unidentified		0.015						
Rhizophlyctidomycetes	Rhizophlyctidales	Rhizophlyctidaceae	*Rhizophlyctis*	unidentified							0.081	

unidentified	unidentified	unidentified	unidentified	unidentified							0.032	
unidentified	unidentified	unidentified	unidentified	unidentified						0.013		
**Mortierellomycota**												
Mortierellomycetes	Mortierellales	Mortierellaceae	*Mortierella*	*Mortierella capitata*						0.054	0.145	
Mortierellomycetes	Mortierellales	Mortierellaceae	*Mortierella*	*Mortierella selenospora*					0.035			
Mortierellomycetes	Mortierellales	unidentified	unidentified	unidentified	0.314		0.314					
Mortierellomycetes	Mortierellales	unidentified	unidentified	unidentified							0.059	
Mortierellomycetes	Mortierellales	unidentified	unidentified	unidentified						0.04		
**unidentified**												
unidentified	unidentified	unidentified	unidentified	unidentified	52.063	2.135	52.063	0.109				
unidentified	unidentified	unidentified	unidentified	unidentified				3.126				
unidentified	unidentified	unidentified	unidentified	unidentified	0.038		0.038					
unidentified	unidentified	unidentified	unidentified	unidentified	0.038		0.038					
unidentified	unidentified	unidentified	unidentified	unidentified				0.159				
unidentified	unidentified	unidentified	unidentified	unidentified				0.159				
unidentified	unidentified	unidentified	unidentified	unidentified					0.062			
unidentified	unidentified	unidentified	unidentified	unidentified							0.086	
unidentified	unidentified	unidentified	unidentified	unidentified							0.086	
unidentified	unidentified	unidentified	unidentified	unidentified	0.024		0.024	0.05				
unidentified	unidentified	unidentified	unidentified	unidentified					0.058			
unidentified	unidentified	unidentified	unidentified	unidentified						0.101		
unidentified	unidentified	unidentified	unidentified	unidentified				2.987				
unidentified	unidentified	unidentified	unidentified	unidentified							0.081	
unidentified	unidentified	unidentified	unidentified	unidentified							0.081	
unidentified	unidentified	unidentified	unidentified	unidentified	0.033		0.033					

unidentified	unidentified	unidentified	unidentified	unidentified		0.105						
unidentified	unidentified	unidentified	unidentified	unidentified				0.139				
unidentified	unidentified	unidentified	unidentified	unidentified						0.094		
unidentified	unidentified	unidentified	unidentified	unidentified	0.014		0.014	0.069				
unidentified	unidentified	unidentified	unidentified	unidentified					0.05			
unidentified	unidentified	unidentified	unidentified	unidentified					0.023		0.038	
unidentified	unidentified	unidentified	unidentified	unidentified						0.088		
unidentified	unidentified	unidentified	unidentified	unidentified	0.556		0.556					
unidentified	unidentified	unidentified	unidentified	unidentified						0.088		
unidentified	unidentified	unidentified	unidentified	unidentified							0.07	
unidentified	unidentified	unidentified	unidentified	unidentified				0.119				
unidentified	unidentified	unidentified	unidentified	unidentified						0.081		
unidentified	unidentified	unidentified	unidentified	unidentified							0.064	
unidentified	unidentified	unidentified	unidentified	unidentified							0.064	
unidentified	unidentified	unidentified	unidentified	unidentified							0.064	
unidentified	unidentified	unidentified	unidentified	unidentified	0.014		0.014	0.05				
unidentified	unidentified	unidentified	unidentified	unidentified		0.082						
unidentified	unidentified	unidentified	unidentified	unidentified					0.043			
unidentified	unidentified	unidentified	unidentified	unidentified					0.178	1.181		
unidentified	unidentified	unidentified	unidentified	unidentified						0.074		
unidentified	unidentified	unidentified	unidentified	unidentified							0.059	
unidentified	unidentified	unidentified	unidentified	unidentified							0.059	
unidentified	unidentified	unidentified	unidentified	unidentified	0.024		0.024					
unidentified	unidentified	unidentified	unidentified	unidentified					0.039			
unidentified	unidentified	unidentified	unidentified	unidentified					0.039			
unidentified	unidentified	unidentified	unidentified	unidentified						0.067		
unidentified	unidentified	unidentified	unidentified	unidentified						0.067		
unidentified	unidentified	unidentified	unidentified	unidentified							0.054	

unidentified	unidentified	unidentified	unidentified	unidentified		0.067						
unidentified	unidentified	unidentified	unidentified	unidentified	0.485		0.485					
unidentified	unidentified	unidentified	unidentified	unidentified				0.089				
unidentified	unidentified	unidentified	unidentified	unidentified				0.089				
unidentified	unidentified	unidentified	unidentified	unidentified					0.035			
unidentified	unidentified	unidentified	unidentified	unidentified					0.035			
unidentified	unidentified	unidentified	unidentified	unidentified						0.061		
unidentified	unidentified	unidentified	unidentified	unidentified						0.061		
unidentified	unidentified	unidentified	unidentified	unidentified						0.061		
unidentified	unidentified	unidentified	unidentified	unidentified							0.048	
unidentified	unidentified	unidentified	unidentified	unidentified							0.048	
unidentified	unidentified	unidentified	unidentified	unidentified							0.048	
unidentified	unidentified	unidentified	unidentified	unidentified					0.22		0.661	
unidentified	unidentified	unidentified	unidentified	unidentified							0.048	
unidentified	unidentified	unidentified	unidentified	unidentified	0.019		0.019					
unidentified	unidentified	unidentified	unidentified	unidentified	0.019		0.019					
unidentified	unidentified	unidentified	unidentified	unidentified	0.019		0.019					
unidentified	unidentified	unidentified	unidentified	unidentified	0.019		0.019					
unidentified	unidentified	unidentified	unidentified	unidentified	0.019		0.019					
unidentified	unidentified	unidentified	unidentified	unidentified		0.06						
unidentified	unidentified	unidentified	unidentified	unidentified				0.079				
unidentified	unidentified	unidentified	unidentified	unidentified							0.043	
unidentified	unidentified	unidentified	unidentified	unidentified		0.052						
unidentified	unidentified	unidentified	unidentified	unidentified				1.776				
unidentified	unidentified	unidentified	unidentified	unidentified				0.069				
unidentified	unidentified	unidentified	unidentified	unidentified				0.069				
unidentified	unidentified	unidentified	unidentified	unidentified				0.069				
unidentified	unidentified	unidentified	unidentified	unidentified				0.069				

unidentified	unidentified	unidentified	unidentified	unidentified					0.027			
unidentified	unidentified	unidentified	unidentified	unidentified					0.027			
unidentified	unidentified	unidentified	unidentified	unidentified						0.047		
unidentified	unidentified	unidentified	unidentified	unidentified						0.047		
unidentified	unidentified	unidentified	unidentified	unidentified						0.047		
unidentified	unidentified	unidentified	unidentified	unidentified						0.047		
unidentified	unidentified	unidentified	unidentified	unidentified	0.376		0.376					
unidentified	unidentified	unidentified	unidentified	unidentified	0.014		0.014					
unidentified	unidentified	unidentified	unidentified	unidentified	0.014		0.014					
unidentified	unidentified	unidentified	unidentified	unidentified	0.014		0.014					
unidentified	unidentified	unidentified	unidentified	unidentified		0.045						
unidentified	unidentified	unidentified	unidentified	unidentified		0.045						
unidentified	unidentified	unidentified	unidentified	unidentified		0.045						
unidentified	unidentified	unidentified	unidentified	unidentified		0.045						
unidentified	unidentified	unidentified	unidentified	unidentified				0.06				
unidentified	unidentified	unidentified	unidentified	unidentified				0.06				
unidentified	unidentified	unidentified	unidentified	unidentified				0.06				
unidentified	unidentified	unidentified	unidentified	unidentified		1.131						
unidentified	unidentified	unidentified	unidentified	unidentified				0.06				
unidentified	unidentified	unidentified	unidentified	unidentified				0.06				
unidentified	unidentified	unidentified	unidentified	unidentified					0.023			
unidentified	unidentified	unidentified	unidentified	unidentified						0.04		
unidentified	unidentified	unidentified	unidentified	unidentified							0.032	
unidentified	unidentified	unidentified	unidentified	unidentified							0.032	
unidentified	unidentified	unidentified	unidentified	unidentified							0.032	
unidentified	unidentified	unidentified	unidentified	unidentified							0.032	
unidentified	unidentified	unidentified	unidentified	unidentified							0.032	
unidentified	unidentified	unidentified	unidentified	unidentified		0.037						

unidentified	unidentified	unidentified	unidentified	unidentified	0.314		0.314					
unidentified	unidentified	unidentified	unidentified	unidentified		0.037						
unidentified	unidentified	unidentified	unidentified	unidentified				0.05				
unidentified	unidentified	unidentified	unidentified	unidentified				0.05				
unidentified	unidentified	unidentified	unidentified	unidentified				0.05				
unidentified	unidentified	unidentified	unidentified	unidentified					0.019			
unidentified	unidentified	unidentified	unidentified	unidentified					0.019			
unidentified	unidentified	unidentified	unidentified	unidentified					0.019			
unidentified	unidentified	unidentified	unidentified	unidentified					0.019			
unidentified	unidentified	unidentified	unidentified	unidentified						0.034		
unidentified	unidentified	unidentified	unidentified	unidentified							0.027	
unidentified	unidentified	unidentified	unidentified	unidentified	3.883		3.883					
unidentified	unidentified	unidentified	unidentified	unidentified					0.039	0.803		
unidentified	unidentified	unidentified	unidentified	unidentified							0.027	
unidentified	unidentified	unidentified	unidentified	unidentified							0.027	
unidentified	unidentified	unidentified	unidentified	unidentified							0.027	
unidentified	unidentified	unidentified	unidentified	unidentified							0.027	
unidentified	unidentified	unidentified	unidentified	unidentified							0.027	
unidentified	unidentified	unidentified	unidentified	unidentified							0.027	
unidentified	unidentified	unidentified	unidentified	unidentified	0.01		0.01					
unidentified	unidentified	unidentified	unidentified	unidentified	0.01		0.01					
unidentified	unidentified	unidentified	unidentified	unidentified	0.01		0.01					
unidentified	unidentified	unidentified	unidentified	unidentified	0.01		0.01					
unidentified	unidentified	unidentified	unidentified	unidentified					0.023		0.661	
unidentified	unidentified	unidentified	unidentified	unidentified	0.01		0.01					
unidentified	unidentified	unidentified	unidentified	unidentified		0.03						
unidentified	unidentified	unidentified	unidentified	unidentified		0.03						
unidentified	unidentified	unidentified	unidentified	unidentified				0.04				

unidentified	unidentified	unidentified	unidentified	unidentified				0.04				
unidentified	unidentified	unidentified	unidentified	unidentified				0.04				
unidentified	unidentified	unidentified	unidentified	unidentified				0.04				
unidentified	unidentified	unidentified	unidentified	unidentified				0.04				
unidentified	unidentified	unidentified	unidentified	unidentified					0.015			
unidentified	unidentified	unidentified	unidentified	unidentified						0.027		
unidentified	unidentified	unidentified	unidentified	unidentified							0.677	
unidentified	unidentified	unidentified	unidentified	unidentified						0.027		
unidentified	unidentified	unidentified	unidentified	unidentified						0.027		
unidentified	unidentified	unidentified	unidentified	unidentified							0.021	
unidentified	unidentified	unidentified	unidentified	unidentified							0.021	
unidentified	unidentified	unidentified	unidentified	unidentified							0.021	
unidentified	unidentified	unidentified	unidentified	unidentified								0.101
unidentified	unidentified	unidentified	unidentified	unidentified		0.022						
unidentified	unidentified	unidentified	unidentified	unidentified		0.022						
unidentified	unidentified	unidentified	unidentified	unidentified		0.022						
unidentified	unidentified	unidentified	unidentified	unidentified		0.022						
unidentified	unidentified	unidentified	unidentified	unidentified					0.197			1.869
unidentified	unidentified	unidentified	unidentified	unidentified		0.022						
unidentified	unidentified	unidentified	unidentified	unidentified		0.022						
unidentified	unidentified	unidentified	unidentified	unidentified				0.03				
unidentified	unidentified	unidentified	unidentified	unidentified				0.03				
unidentified	unidentified	unidentified	unidentified	unidentified					0.012			
unidentified	unidentified	unidentified	unidentified	unidentified					0.012			
unidentified	unidentified	unidentified	unidentified	unidentified					0.012			
unidentified	unidentified	unidentified	unidentified	unidentified						0.02		
unidentified	unidentified	unidentified	unidentified	unidentified						0.02		
unidentified	unidentified	unidentified	unidentified	unidentified							0.016	

unidentified	unidentified	unidentified	unidentified	unidentified	0.271		0.271	0.06				
unidentified	unidentified	unidentified	unidentified	unidentified							0.016	
unidentified	unidentified	unidentified	unidentified	unidentified							0.016	
unidentified	unidentified	unidentified	unidentified	unidentified							0.016	
unidentified	unidentified	unidentified	unidentified	unidentified							0.016	
unidentified	unidentified	unidentified	unidentified	unidentified							0.016	
unidentified	unidentified	unidentified	unidentified	unidentified							0.016	
unidentified	unidentified	unidentified	unidentified	unidentified							0.016	
unidentified	unidentified	unidentified	unidentified	unidentified							0.016	
unidentified	unidentified	unidentified	unidentified	unidentified		0.015						
unidentified	unidentified	unidentified	unidentified	unidentified		0.015						
unidentified	unidentified	unidentified	unidentified	unidentified				1.181				
unidentified	unidentified	unidentified	unidentified	unidentified		0.015						
unidentified	unidentified	unidentified	unidentified	unidentified				0.02				
unidentified	unidentified	unidentified	unidentified	unidentified				0.02				
unidentified	unidentified	unidentified	unidentified	unidentified				0.02				
unidentified	unidentified	unidentified	unidentified	unidentified				0.02				
unidentified	unidentified	unidentified	unidentified	unidentified					0.008			
unidentified	unidentified	unidentified	unidentified	unidentified					0.008			
unidentified	unidentified	unidentified	unidentified	unidentified					0.008			
unidentified	unidentified	unidentified	unidentified	unidentified						0.013		
unidentified	unidentified	unidentified	unidentified	unidentified						0.013		
unidentified	unidentified	unidentified	unidentified	unidentified	0.114	0.509	0.114					
unidentified	unidentified	unidentified	unidentified	unidentified						0.013		
unidentified	unidentified	unidentified	unidentified	unidentified				1.121				
unidentified	unidentified	unidentified	unidentified	unidentified						0.749		
unidentified	unidentified	unidentified	unidentified	unidentified					0.414			
unidentified	unidentified	unidentified	unidentified	unidentified					0.885		3.846	

unidentified	unidentified	unidentified	unidentified	unidentified	0.228		0.228	0.079				
unidentified	unidentified	unidentified	unidentified	unidentified						0.648		
unidentified	unidentified	unidentified	unidentified	unidentified	0.223		0.223					
unidentified	unidentified	unidentified	unidentified	unidentified				0.873				
unidentified	unidentified	unidentified	unidentified	unidentified	0.176		0.176	0.089				
unidentified	unidentified	unidentified	unidentified	unidentified		0.614						
unidentified	unidentified	unidentified	unidentified	unidentified						0.533		
unidentified	unidentified	unidentified	unidentified	unidentified					0.089	0.337		
unidentified	unidentified	unidentified	unidentified	unidentified						0.391		
unidentified	unidentified	unidentified	unidentified	unidentified						0.385		
unidentified	unidentified	unidentified	unidentified	unidentified		6.875						
unidentified	unidentified	unidentified	unidentified	unidentified				0.546				
unidentified	unidentified	unidentified	unidentified	unidentified						0.371		
unidentified	unidentified	unidentified	unidentified	unidentified							0.29	
unidentified	unidentified	unidentified	unidentified	unidentified						0.344		
unidentified	unidentified	unidentified	unidentified	unidentified	0.119		0.119					
unidentified	unidentified	unidentified	unidentified	unidentified						0.324		
unidentified	unidentified	unidentified	unidentified	unidentified	0.086	0.075	0.086					
unidentified	unidentified	unidentified	unidentified	unidentified					0.178			
unidentified	unidentified	unidentified	unidentified	unidentified						0.304		
unidentified	unidentified	unidentified	unidentified	unidentified		0.045		0.377				
unidentified	unidentified	unidentified	unidentified	unidentified	1.878	0.232	1.878	0.873				
unidentified	unidentified	unidentified	unidentified	unidentified						0.297		
unidentified	unidentified	unidentified	unidentified	unidentified				0.427				
unidentified	unidentified	unidentified	unidentified	unidentified								1.086
unidentified	unidentified	unidentified	unidentified	unidentified				0.417				
unidentified	unidentified	unidentified	unidentified	unidentified		0.307						
unidentified	unidentified	unidentified	unidentified	unidentified		0.3						

unidentified	unidentified	unidentified	unidentified	unidentified					0.155			
unidentified	unidentified	unidentified	unidentified	unidentified	0.076		0.076	0.069				
unidentified	unidentified	unidentified	unidentified	unidentified	0.09		0.09					
unidentified	unidentified	unidentified	unidentified	unidentified	0.067	0.067	0.067					
unidentified	unidentified	unidentified	unidentified	unidentified	1.05		1.05					
unidentified	unidentified	unidentified	unidentified	unidentified	0.052		0.052	0.149				
unidentified	unidentified	unidentified	unidentified	unidentified					0.089	0.094		
unidentified	unidentified	unidentified	unidentified	unidentified		0.195				0.061		
unidentified	unidentified	unidentified	unidentified	unidentified								0.884
unidentified	unidentified	unidentified	unidentified	unidentified	0.076	0.015	0.076					
unidentified	unidentified	unidentified	unidentified	unidentified					0.128			
unidentified	unidentified	unidentified	unidentified	unidentified						0.209		
unidentified	unidentified	unidentified	unidentified	unidentified						0.202		
unidentified	unidentified	unidentified	unidentified	unidentified	0.067		0.067					
unidentified	unidentified	unidentified	unidentified	unidentified		0.112		0.089	0.015			
unidentified	unidentified	unidentified	unidentified	unidentified		2.786						
unidentified	unidentified	unidentified	unidentified	unidentified	0.062		0.062					
unidentified	unidentified	unidentified	unidentified	unidentified				0.258				
unidentified	unidentified	unidentified	unidentified	unidentified					0.1			
unidentified	unidentified	unidentified	unidentified	unidentified				0.248				
unidentified	unidentified	unidentified	unidentified	unidentified	0.057		0.057					
unidentified	unidentified	unidentified	unidentified	unidentified					0.093			
unidentified	unidentified	unidentified	unidentified	unidentified				0.228				
unidentified	unidentified	unidentified	unidentified	unidentified						0.155		
unidentified	unidentified	unidentified	unidentified	unidentified				0.218				
unidentified	unidentified	unidentified	unidentified	unidentified				0.218				
unidentified	unidentified	unidentified	unidentified	unidentified	0.247	1.693	0.247					
unidentified	unidentified	unidentified	unidentified	unidentified					0.085			

unidentified	unidentified	unidentified	unidentified	unidentified							0.118	
unidentified	unidentified	unidentified	unidentified	unidentified							0.118	
unidentified	unidentified	unidentified	unidentified	unidentified							0.118	
unidentified	unidentified	unidentified	unidentified	unidentified		0.157						
unidentified	unidentified	unidentified	unidentified	unidentified				0.208				
unidentified	unidentified	unidentified	unidentified	unidentified				0.198				
unidentified	unidentified	unidentified	unidentified	unidentified					0.077			
unidentified	unidentified	unidentified	unidentified	unidentified							0.107	
unidentified	unidentified	unidentified	unidentified	unidentified							0.107	
unidentified	unidentified	unidentified	unidentified	unidentified		2.464						
unidentified	unidentified	unidentified	unidentified	unidentified						0.128		
unidentified	unidentified	unidentified	unidentified	unidentified				0.179				
unidentified	unidentified	unidentified	unidentified	unidentified						0.121		
unidentified	unidentified	unidentified	unidentified	unidentified							0.097	
unidentified	unidentified	unidentified	unidentified	unidentified				0.169				
unidentified	unidentified	unidentified	unidentified	unidentified						0.115		
unidentified	unidentified	unidentified	unidentified	unidentified							0.091	
unidentified	unidentified	unidentified	unidentified	unidentified								0.429
unidentified	unidentified	unidentified	unidentified	unidentified	0.038		0.038					
unidentified	unidentified	unidentified	unidentified	unidentified	0.038		0.038					
