# Supplementary material for: Hidden Tenants: Microbiota of the Rhizosphere and Phyllosphere of Cordia dodecandra Trees in Mayan Forests and Homegardens
Source: Plants (Basel). 2022 Nov 15;11(22):3098. doi: 10.3390/plants11223098 (PMC9699097; doi:10.3390/plants11223098)
Supplement: Supplementary file 1 [file plants-11-03098-s001.zip › plants-1975968-Supplementary material-Figures.pdf]

Hidden Tenants: Microbiota of the Rhizosphere and Phyllosphere of *Cordia dodecandra* Trees in Mayan Forests and Homegardens

Carla G. May-Mutul, Miguel A. López-Garrido, Aileen O'Connor-Sánchez, Yuri J. Peña-Ramírez, Natalia Y. Labrín-Sotomayor, Héctor Estrada-Medina and Miriam M. Ferrer

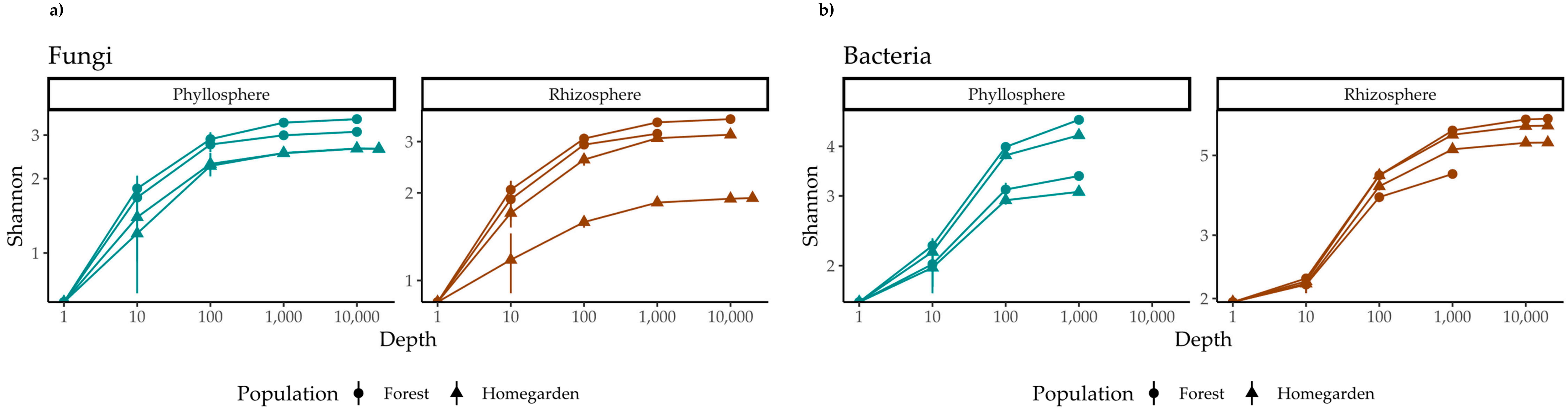

Figure S1: Rarefaction curves depicting the observed frequency of operating taxonomic units (OTUs) by the sequencing depth in bacteria and fungi in a) bacteria and b) fungi microbiota from phyllosphere and rhizosphere, in Yucatán´s forest and homegarden populations of *Cordia dodecandra*.

.

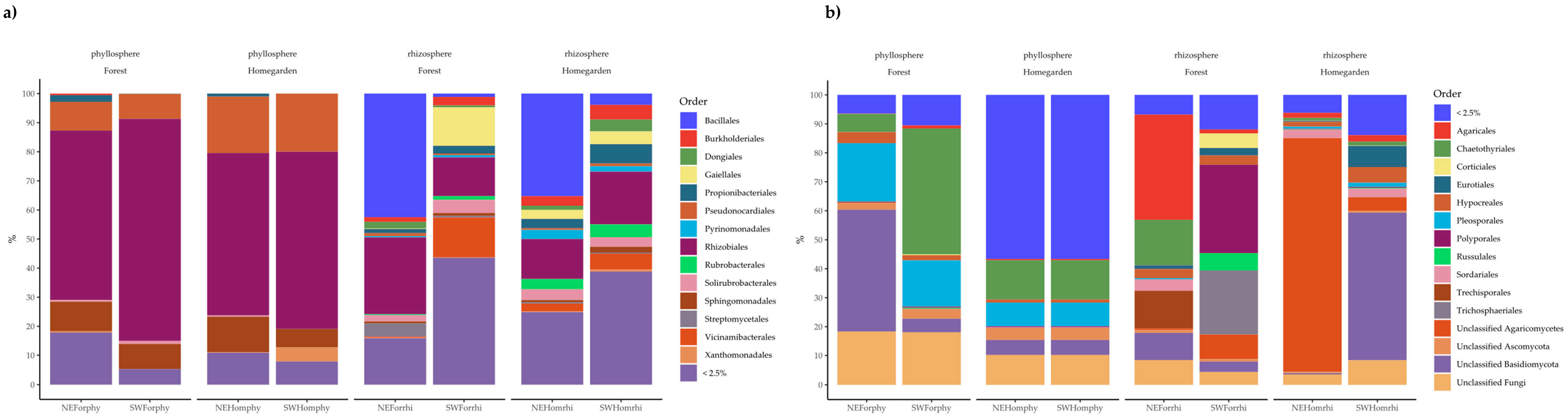

Figure S2: Relative frequency of orders assigned to a) 618 bacteria and b) 1096 fungi OTUs from phyllosphere and rhizosphere microbiota in Yucatan´s forest and homegarden populations of *Cordia dodecandra*.

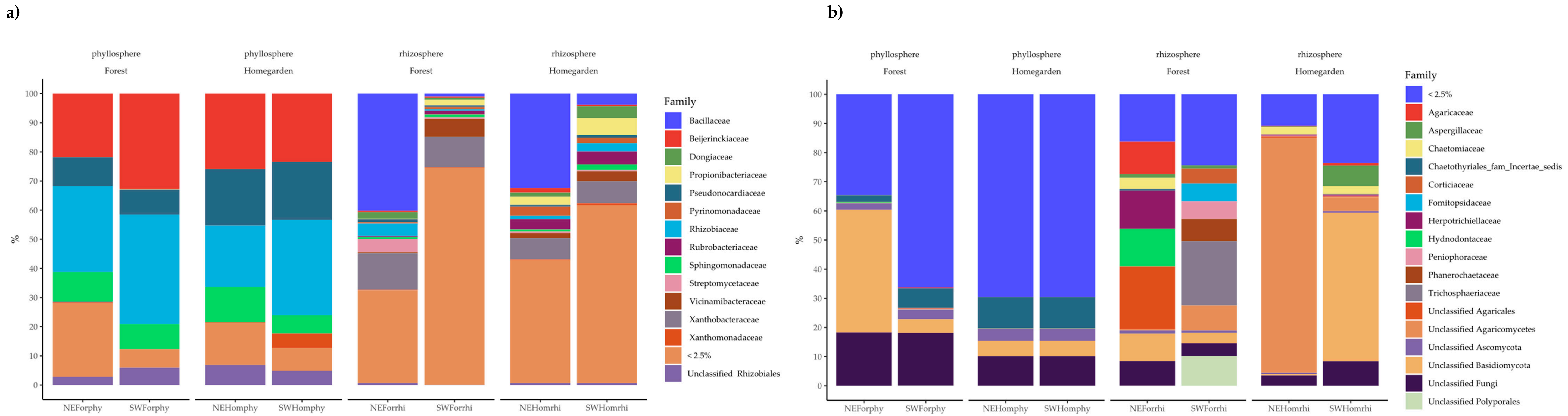

Figure S3: Relative frequency of families assigned to a) 618 bacteria and b) 1096 fungi OTUs from phyllosphere and rhizosphere microbiota in Yucatan´s forest and homegarden populations of *Cordia dodecandra*.

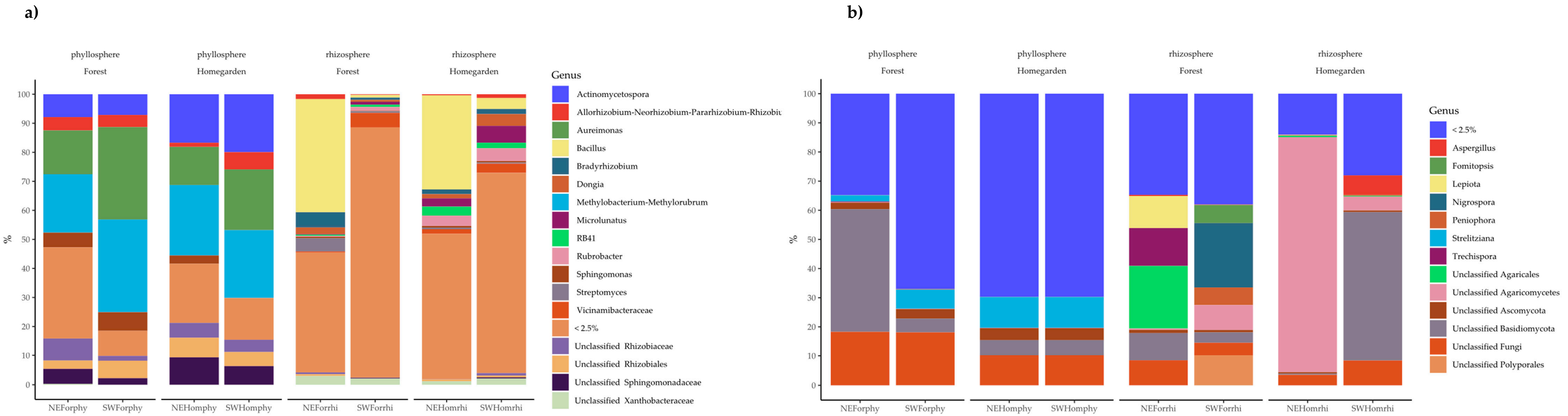

Figure S4: Relative frequency of genus assigned to a) 618 bacteria and b) 1096 fungi OTUs from phyllosphere and rhizosphere microbiota in Yucatan´s forest and homegarden populations of *Cordia dodecandra*.
